# Clouds and Convective Self‐Aggregation in a Multimodel Ensemble of Radiative‐Convective Equilibrium Simulations

**DOI:** 10.1029/2020MS002138

**Published:** 2020-09-18

**Authors:** Allison A. Wing, Catherine L. Stauffer, Tobias Becker, Kevin A. Reed, Min‐Seop Ahn, Nathan P. Arnold, Sandrine Bony, Mark Branson, George H. Bryan, Jean‐Pierre Chaboureau, Stephan R. De Roode, Kulkarni Gayatri, Cathy Hohenegger, I‐Kuan Hu, Fredrik Jansson, Todd R. Jones, Marat Khairoutdinov, Daehyun Kim, Zane K. Martin, Shuhei Matsugishi, Brian Medeiros, Hiroaki Miura, Yumin Moon, Sebastian K. Müller, Tomoki Ohno, Max Popp, Thara Prabhakaran, David Randall, Rosimar Rios‐Berrios, Nicolas Rochetin, Romain Roehrig, David M. Romps, James H. Ruppert, Masaki Satoh, Levi G. Silvers, Martin S. Singh, Bjorn Stevens, Lorenzo Tomassini, Chiel C. van Heerwaarden, Shuguang Wang, Ming Zhao

**Affiliations:** ^1^ Department of Earth, Ocean and Atmospheric Science Florida State University Tallahassee FL USA; ^2^ Max Planck Institute for Meteorology Hamburg Germany; ^3^ School of Marine and Atmospheric Sciences Stony Brook University Stony Brook NY USA; ^4^ Department of Atmospheric Sciences University of Washington Seattle WA USA; ^5^ Global Modeling and Assimilation Office NASA Goddard Space Flight Center Greenbelt MD USA; ^6^ Laboratoire de Météorologie Dynamique (LMD)/IPSL/Sorbonne Université/CNRS Paris France; ^7^ Department of Atmospheric Science Colorado State University Fort Collins CO USA; ^8^ National Center for Atmospheric Research Boulder CO USA; ^9^ Laboratoire d'Aérologie, Université de Toulouse, CNRS, UPS Toulouse France; ^10^ Faculty of Civil Engineering and Geosciences, Department of Geoscience and Remote Sensing Delft University of Technology Delft Netherlands; ^11^ Indian Institute of Tropical Meteorology Pune India; ^12^ Rosenstiel School of Marine and Atmospheric Science University of Miami Miami FL USA; ^13^ Centrum Wiskunde and Informatica Amsterdam Netherlands; ^14^ Department of Meteorology University of Reading Reading UK; ^15^ School of Marine and Atmospheric Sciences, and Institute for Advanced Computational Science, Stony Brook University State University of New York Stony Brook NY USA; ^16^ Department of Applied Physics and Applied Mathematics Columbia University New York NY USA; ^17^ Atmosphere and Ocean Research Institute The University of Tokyo Kashiwa Japan; ^18^ Department of Earth and Planetary Science, Graduate School of Science The University of Tokyo Tokyo Japan; ^19^ Japan Agency for Marine‐Earth Science and Technology Yokohama Japan; ^20^ Laboratoire de Météorologie Dynamique (LMD)/IPSL/Sorbonne Université/CNRS/École Polytechnique/École Normale Supérieure Paris France; ^21^ CNRM, Université de Toulouse, Météo‐France, CNRS Toulouse France; ^22^ Department of Earth and Planetary Science University of California Berkeley CA USA; ^23^ Climate and Ecosystem Sciences Division Lawrence Berkeley National Laboratory Berkeley CA USA; ^24^ Department of Meteorology and Atmospheric Science and Center for Advanced Data Assimilation and Predictability Techniques Pennsylvania State University University Park PA USA; ^25^ School of Earth, Atmosphere, and Environment Monash University Clayton Victoria Australia; ^26^ Met Office Exeter UK; ^27^ Meteorology and Air Quality Group Wageningen University Wageningen Netherlands; ^28^ NOAA/Geophysical Fluid Dynamics Laboratory Princeton NJ USA

**Keywords:** convection, clouds, climate sensitivity, self‐aggregation, radiative‐convective equilibrium, cloud feedbacks

## Abstract

The Radiative‐Convective Equilibrium Model Intercomparison Project (RCEMIP) is an intercomparison of multiple types of numerical models configured in radiative‐convective equilibrium (RCE). RCE is an idealization of the tropical atmosphere that has long been used to study basic questions in climate science. Here, we employ RCE to investigate the role that clouds and convective activity play in determining cloud feedbacks, climate sensitivity, the state of convective aggregation, and the equilibrium climate. RCEMIP is unique among intercomparisons in its inclusion of a wide range of model types, including atmospheric general circulation models (GCMs), single column models (SCMs), cloud‐resolving models (CRMs), large eddy simulations (LES), and global cloud‐resolving models (GCRMs). The first results are presented from the RCEMIP ensemble of more than 30 models. While there are large differences across the RCEMIP ensemble in the representation of mean profiles of temperature, humidity, and cloudiness, in a majority of models anvil clouds rise, warm, and decrease in area coverage in response to an increase in sea surface temperature (SST). Nearly all models exhibit self‐aggregation in large domains and agree that self‐aggregation acts to dry and warm the troposphere, reduce high cloudiness, and increase cooling to space. The degree of self‐aggregation exhibits no clear tendency with warming. There is a wide range of climate sensitivities, but models with parameterized convection tend to have lower climate sensitivities than models with explicit convection. In models with parameterized convection, aggregated simulations have lower climate sensitivities than unaggregated simulations.

## Key Points

- Temperature, humidity, and clouds in radiative‐convective equilibrium vary substantially across models
- Models agree that self‐aggregation dries the atmosphere and reduces high cloudiness
- There is no consistency in how self‐aggregation depends on warming

## 1 Introduction

For more than 20 years, coordinated model intercomparisons have been undertaken in which simulations with consistent configurations have been performed with different models to assess whether different models behave similarly and to aid in the understanding of relevant phenomenon. Many of these intercomparisons have been performed with global climate models, such as the Coupled Model Intercomparison Project (CMIP; Eyring et al., 2016; Meehl et al., 1997, 2000, 2007; Taylor et al., 2012), which was designed to assess the ability of global climate models to robustly simulate important features of the current climate and to evaluate potential future climate changes. The most recent phase (CMIP6; Eyring et al., 2016) includes 21 additional CMIP‐Endorsed Model Intercomparison Projects, which address more targeted scientific questions. Examples include the Cloud Feedback Model Intercomparison Project (CFMIP; Webb et al., 2017), which aims to reduce uncertainty in cloud feedbacks, and the High‐Resolution Model Intercomparison Project (HighResMIP; Haarsma et al., 2016), which investigates the impact of horizontal resolution on regional climate and smaller‐scale phenomena. There are also intercomparisons that exist outside the CMIP infrastructure, some of which employ versions of global models with idealized boundary conditions (e.g., Voigt et al., 2016) or compare specific components of global models, such as the dynamical core (Ullrich et al., 2017). All of the global models that have participated in the aforementioned intercomparisons make use of subgrid‐scale parameterizations, and so another class of intercomparisons that have been influential in the process‐based development of parameterizations is those comparing cloud‐resolving or large eddy simulations of an observational case to single column versions of global models (e.g., Browning et al., 1993; Moeng et al., 1996). Some examples include the CFMIP‐Global Atmospheric System Studies (GASS) Intercomparison of Large eddy models and Single column models (CGILS;  Blossey et al., 2013; Zhang et al., 2012, 2013), which compared for the first time low cloud feedbacks predicted by models with and without cloud and convective parameterizations, the Global Energy and Water Exchanges project (GEWEX) Atmospheric Boundary Layer Study (GABLS; Bazile et al., 2014; Bosveld et al., 2014; Cuxart et al., 2006; Svensson et al., 2011), which investigated the atmospheric boundary layer, and the European Union Cloud Intercomparison, Process Study, and Evaluation project (EUCLIPSE)‐GASS intercomparison on the stratocumulus to cumulus transition (de Roode et al., 2016; Neggers et al., 2017). More recently, the transition of global modeling to kilometer‐scale resolution has motivated the first intercomparison of global cloud‐resolving models (DYAMOND;  Stevens et al., 2019).

To date, all model intercomparisons, including those listed above, have been limited to usually one, or at most two, different types of models, rather than incorporating a model hierarchy. This is likely a result of the inherent difficulty in configuring different classes of models in a consistent manner, particularly in complex Earth‐like settings. The Radiative‐Convective Equilibrium Model Intercomparison Project (RCEMIP; Wing et al., 2018) overcomes this limitation by employing the idealized framework of radiative‐convective equilibrium (RCE), which is accessible to nearly every conceivable atmospheric model type. To our knowledge, there is no other model intercomparison project that has incorporated such a varied range of model types, including general circulation models (GCMs), single‐column models (SCMs), cloud‐resolving models (CRMs), large eddy simulations (LES), and global cloud‐resolving models (GCRMs). Thus, RCEMIP presents an unprecedented opportunity to compare models with and without convective parameterizations and in a variety of domain configurations on equal footing.

RCE is the simplest possible description of the climate system, in which radiative cooling of the atmosphere is on average balanced by convective heating. Despite, or perhaps because of its simplicity, there is a long history of modeling RCE in one‐dimensional models (e.g., Manabe & Strickler, 1964; Möller, 1963), two‐ and three‐dimensional CRMs with explicit convection (e.g., Bretherton et al., 2005; Held et al., 1993; Nakajima & Matsuno, 1988; Tompkins & Craig, 1998a), and GCMs with parameterized clouds and convection (e.g., Held et al., 2007; Popke et al., 2013; Reed et al., 2015). RCE remains a popular setting in which to phrase fundamental scientific questions about climate because it eliminates the complexity of imposed heterogeneities in boundary conditions and forcings (and the resulting dynamical instabilities) but retains the full complexity of moist convective processes and their interaction with radiation and circulation. An initial motivation for the renewed interest in RCE is that insights from more fundamental models might improve more coarse‐grained descriptions of the same phenomena and thus contribute to model development (Popke et al., 2013; Reed & Medeiros, 2016). The importance of using a hierachy of models to understand the climate system, in which understanding is built in simpler contexts that can be connected to more complex systems, has been emphasized by Held (2005, 2014). RCE's status as the simplest representation of the climate system makes it an essential inclusion in such hierarchies (Jeevanjee et al., 2017; Maher et al., 2019). In addition to these formal issues, an intriguing result emerging from cloud‐resolving simulations of RCE is that the interaction between clouds and circulations can give rise to self‐aggregation of convection, but its importance for climate and the relative role of different driving mechanisms remain unclear and seemingly model dependent (Wing, 2019; Wing et al., 2017).

As described in Wing et al. (2018), RCEMIP was motivated by the absence of a common baseline in past simulations of RCE, the accessibility of RCE to a wide range of model types, and the utility of RCE as a framework in which to address some of the biggest open questions in climate science (Bony et al., 2015). With this in mind, RCEMIP was designed to address the following three themes:

1. The robustness of the RCE state across the spectrum of models.
2. The response of clouds to warming and the resulting climate sensitivity in RCE.
3. The dependence of convective self‐aggregation on surface temperature.

While clouds, climate sensitivity, and convective self‐aggregation have all been investigated in RCE before, previous studies have differed in many ways, which has made it difficult to assess whether the diverse results are a real reflection of model uncertainty and lack of understanding or if they are artifacts of the experimental design and/or choice of diagnostics. For example, some modeling studies have found that the spatial extent of tropical anvil clouds decreases with warming (Bony et al., 2016; Cronin & Wing, 2017; Kuang & Hartmann, 2007; Tompkins & Craig, 1999) while others have found an increase (Chen et al., 2016; Ohno & Satoh, 2018; Ohno et al., 2019; Singh & O'Gorman, 2015; Tsushima et al., 2014). Convective self‐aggregation, which is the spontaneous organization of convection despite homogeneous boundary conditions and forcing, has been found to occur across many different models (as reviewed by Wing et al., 2017), but there are some models that do not exhibit spontaneous aggregation under some conditions (Jeevanjee & Romps, 2013), and there is disagreement on the details of the physical mechanisms (Arnold & Randall, 2015; Holloway & Woolnough, 2016; Muller & Bony, 2015; Wing & Emanuel, 2014; Wing et al., 2017). The manner and extent to which self‐aggregation is temperature dependent also remain unresolved, with some studies finding that self‐aggregation is favored by high temperatures and others finding no clear temperature dependence, as reviewed by Wing (2019). Several studies have suggested that self‐aggregation, through its effect on humidity and cloudiness, may modulate climate sensitivity (Becker et al., 2017; Coppin & Bony, 2018; Cronin & Wing, 2017; Hohenegger & Stevens, 2016; Mauritsen & Stevens, 2015) and recent estimates from GCMs configured in RCE suggest a climate sensitivity similar to but slightly lower than that of the tropics in comprehensive simulations (Becker & Stevens, 2014; Popke et al., 2013; Silvers et al., 2016). However, the variety of ways in which climate sensitivity in RCE was estimated in these studies, including the type of forcing, the climate perturbation, the background state, and whether the model is uncoupled or coupled to an ocean (usually an idealized slab ocean) at the lower boundary, impedes their interpretation. RCEMIP addresses many of these issues through the specification of a standard protocol.

The objective of this paper is to provide a first broad overview of the RCEMIP simulations, with a focus on documenting the RCEMIP ensemble and characterizing the RCE state and its robustness across the spectrum of models. We discuss each of the three above‐mentioned RCEMIP themes and point out notable patterns of behavior, such as if models with explicit convection behave differently than models with parameterized convection. However, it is beyond the scope of this paper to provide an explanation for the intermodel spread or the behavior of any one individual model or to investigate detailed physical mechanisms for changes in response to warming and rigorously test scaling hypotheses. Consistency with prior work is pointed out where appropriate, but detailed investigation of causality is left to future work that can thoroughly investigate an individual process or model. The results presented in the current paper are a small fraction of the topics that can be explored with the RCEMIP ensemble, so, in addition to serving as a reference for the RCEMIP simulations, we hope that this paper will also stimulate studies into more specific questions and process studies that may require additional experimentation.

While the RCEMIP protocol is described comprehensively in Wing et al. (2018), here we provide details of the configurations of each model participating in RCEMIP and adjustments to the RCEMIP protocol that emerged through the process of its execution (section 2). Section 3 provides a qualitative overview of the ensemble. Section 4 focuses on domain‐ and time‐averaged quantities that characterize the RCE state in the simulations with a surface temperature of 300 K. Self‐aggregation of convection is diagnosed in section  5, including its impact on the mean state. Section 6 describes the response of clouds, self‐aggregation, and the radiative budget to warming by leveraging the suite of simulations performed across three sea surface temperatures (SSTs). Conclusions are presented in section 7.

## 2 RCEMIP Simulations

The RCEMIP protocol is described in Wing et al. (2018); here we briefly review the configuration that is summarized in Table 1. A list of the models participating in RCEMIP is provided in Table 2. Text S1 in the supporting information provides additional details of the configuration of each model.

**Table 1:** Simulation Configuration

Simulation type	Model type	Convection	Domain size	Grid spacing	Vertical levels
RCE_small	CRM	Explicit	∼100 × ∼100 km^2^	1 km	∼74
RCE_small	SCM	Parameterized	Single column	N/A	as in CMIP6
RCE_large	CRM	Explicit	∼6,000 × ∼400 km^2^	3 km	∼74
RCE_large	GCRM	Explicit	Reduced sphere global	∼3‐4 km	∼74
RCE_large	GCM	Parameterized	Global	∼1°	as in CMIP6
RCE_large	WRF‐GCM	Parameterized	∼6000 × ∼400 km^2^	50 km	48
RCE_small_vert	CRM	Explicit	∼100 × ∼100 km^2^	1 km	∼146
RCE_small_les	LES	Explicit	∼100 × ∼100 km^2^	200 m	∼146

RCEMIP consists of simulations at three different SSTs (SST = 295, 300, and 305 K) in two different domain configurations (RCE_small and RCE_large) for a total of six simulations for each model (Table 1). Models are configured as aquaplanets, with no land or sea ice and a fixed, uniform SST; these are atmosphere‐only simulations with no planetary rotation. The solar insolation is made spatially uniform by fixing the solar zenith angle and solar constant; there is no diurnal or seasonal cycle, and the insolation is everywhere equal to the tropical annual mean (409.6 W m^−2^). All trace‐gas concentrations other than water vapor are fixed (Wing et al., 2018) and spatially uniform, and there are no aerosol radiative effects, but shortwave and longwave radiative heating rates are calculated interactively from the modeled state using the individual model's radiation scheme. Surface fluxes are calculated interactively from the resolved surface wind speed and air‐sea enthalpy disequilibrium. The RCE_small simulations are initialized from an analytic approximation to the moist tropical sounding of Dunion (2011). In most cases, the RCE_large simulations are initialized from a domain and time average of the equilibrium state in the corresponding RCE_smallsimulation, though there are a few exceptions in which GCMs that do not have a corresponding RCE_small configuration use a different initialization procedure. The initial temperature and moisture sounding is identical at every grid point with zero initial wind; symmetry is broken and convection is generated by applying random, thermal noise in the lowest model layers.

**Table 2:** Participating Models

Model abbreviation	Model name/version	Model type
CM1	Cloud Model 1, cm1r19.6	CRM/LES
DALES	Dutch Atmospheric Large‐Eddy Simulation model v4.2	CRM/LES
DALES‐damping	Dutch Atmospheric Large‐Eddy Simulation model v4.2	CRM
DAM	Das Atmosphaerische Modell	CRM
FV3	GFDL‐FV3CRM	CRM
ICON‐LEM	ICOsahedral Nonhydrostatic‐2.3.00, LEM config.	CRM/LES
ICON‐NWP	ICOsahedral Nonhydrostatic‐2.3.00, NWP config.	CRM
MESONH	Meso‐NH v5.4.1	CRM/LES
MicroHH	MicroHH v2.0	CRM/LES
SAM‐CRM	System for Atmospheric Modeling 6.11.2	CRM/LES
SCALE	SCALE v5.2.5	CRM
UCLA‐CRM	UCLA Large‐Eddy Simulation model	CRM
UKMO‐CASIM	UK Met Office Idealized Model v11.0 ‐ CASIM	CRM
UKMO‐RA1‐T	UK Met Office Idealized Model v11.0 ‐ RA1‐T	CRM
UKMO‐RA1‐T‐hrad	UK Met Office Idealized Model v11.0 ‐ RA1‐T	CRM
UKMO‐RA1‐T‐nocloud	UK Met Office Idealized Model v11.0 ‐ RA1‐T	CRM
WRF‐COL‐CRM	Weather Research and Forecasting model v3.5.1	CRM
WRF‐CRM	Weather Research and Forecasting model v3.9.1	CRM
MPAS	Model for Prediction Across Scales v6.1	GCRM
NICAM	Non‐hydrostatic Icosahedral Atmospheric Model v16.3	GCRM
SAM‐GCRM	System for Atmospheric Modeling v7.3	GCRM
CAM5‐GCM	Community Atmosphere Model v5	GCM/SCM
CAM6‐GCM	Community Atmosphere Model v6	GCM/SCM
CNRM‐CM6‐1	Atmospheric component of the CNRM Climate Model 6.1	GCM/SCM
ECHAM6‐GCM	MPI‐M Earth System Model‐Atmosphere component v6.3.04p1	GCM
GEOS‐GCM	Goddard Earth Observing System model v5.21	GCM/SCM
ICON‐GCM	ICOsahedral Nonhydrostatic Earth System Model‐Atmosphere component	GCM
IPSL‐CM6	IPSL‐CM6A‐LR	GCM
SAM0‐UNICON	Seoul National University Atmosphere Model v0	GCM
SP‐CAM	Super‐Parameterized Community Atmosphere Model	GCM
SPX‐CAM	Multi‐instance Super‐Parameterized CAM	GCM
UKMO‐GA7.1	UK Met Office Unified Model Global Atmosphere v7.1	GCM/SCM
WRF‐GCM‐cps0	Weather Research and Forecasting model v3.5.1 ‐ no conv. param.	GCM
WRF‐GCM‐cps1	Weather Research and Forecasting model v3.5.1 ‐ KF	GCM
WRF‐GCM‐cps2	Weather Research and Forecasting model v3.5.1 ‐ BMJ	GCM
WRF‐GCM‐cps3	Weather Research and Forecasting model v3.5.1 ‐ GF	GCM
WRF‐GCM‐cps4	Weather Research and Forecasting model v3.5.1 ‐ SAS	GCM
WRF‐GCM‐cps6	Weather Research and Forecasting model v3.5.1 ‐ Tiedtke	GCM

The first class of models that participate in RCEMIP are those with explicit convection, which includes CRMs, GCRMs, and LES (Table 2). CRMs employ a three‐dimensional planar domain with doubly periodic lateral boundary conditions; for RCE_small, the domain is a square of ∼100 × ∼100 km^2^ with a horizontal grid spacing of 1 km, while for RCE_large, the domain is an elongated channel of ∼6,000 × ∼400 km^2^ with a horizontal grid spacing of 3 km (Table 1). We expect that self‐aggregation will be suppressed in RCE_small and more easily triggered in RCE_large due to the latter's larger domain and coarser resolution (Muller & Bony, 2015; Muller & Held, 2012), though it is unknown if all models exhibit these dependencies a priori. The model top is at ∼33 km with ∼74 vertical levels (the vertical levels are specified in Wing et al., 2018). Due to numerical and model configuration constraints, each model does not have precisely the same domain size or number of grid points but instead uses values as close as possible to those given above (Text S1). The simulations are run for 100 days. Three models perform GCRM simulations: MPAS, NICAM, and SAM‐GCRM. To reduce the computational expense of the simulations, MPAS performed global simulations with a reduced Earth radius of *R*_*E*_/8 while NICAM and SAM‐GCRM employ a reduced Earth radius of *R*_*E*_/4. As with the CRMs, the GCRM simulations have grid spacings of ∼3–4 km and ∼74 vertical levels and are run for 100 days (Table 1; Text S1).

Six models perform LES in addition to the CRM simulations (Table 2). These experiments use the ∼100 × ∼100 km^2^ square domain of the RCE_small setup, but with more vertical levels, smaller horizontal grid spacing, and, in some cases, different parameterizations for subgrid‐scale turbulence. The first set of experiments, RCE_small_vert, performed at each of the three SSTs, are identical to RCE_small but have approximately double the number of vertical levels (Table 1). The specified 146 levels are in Table 3 and feature a stretched grid with 26 levels in the lowest 3 km, 200 m vertical grid spacing from 3 to 22 km, stretched to 500 m at 25 km, and then 500 m between there and the model top of 33 km (note that some models may use slightly different levels due to their unique configurations). The second set of simulations, RCE_small_les, performed at each of the three SSTs, has the same vertical levels as RCE_small_vert but use 200 m horizontal grid spacing (Table 1). These simulations are initialized from the mean profiles of the equilibrated RCE_small_vert simulation at the corresponding SST and are run for 50 days.

**Table 3:** Vertical Levels for RCE_small_vert and RCE_small_les

Level (m)	Height (m)	Level	Height (m)	Level	Height (m)	Level	Height
1	20	47	7,200	93	16,400	139	29,500
2	60	48	7,400	94	16,600	140	30,000
3	107	49	7,600	95	16,800	141	30,500
4	160	50	7,800	96	17,000	142	31,000
5	220	51	8,000	97	17,200	143	31,500
6	286	52	8,200	98	17,400	144	32,000
7	359	53	8,400	99	17,600	145	32,500
8	439	54	8,600	100	17,800	146	33,000
9	525	55	8,800	101	18,000
10	618	56	9,000	102	18,200
11	717	57	9,200	103	18,400
12	823	58	9,400	104	18,600
13	936	59	9,600	105	18,800
14	1,055	60	9,800	106	19,000
15	1,181	61	10,000	107	19,200
16	1,314	62	10,200	108	19,400
17	1,453	63	10,400	109	19,600
18	1,599	64	10,600	110	19,800
19	1,751	65	10,800	111	20,000
20	1,910	66	11,000	112	20,200
21	2,076	67	11,200	113	20,400
22	2,248	68	11,400	114	20,600
23	2,427	69	11,600	115	20,800
24	2,612	70	11,800	116	21,000
25	2,804	71	12,000	117	21,200
26	3,000	72	12,200	118	21,400
27	3,200	73	12,400	119	21,600
28	3,400	74	12,600	120	21,800
29	3,600	75	12,800	121	22,000
30	3,800	76	13,000	122	22,220
31	4,000	77	13,200	123	22,463
32	4,200	78	13,400	124	22,730
33	4,400	79	13,600	125	23,023
34	4,600	80	13,800	126	23,347
35	4,800	81	14,000	127	23,703
36	5,000	82	14,200	128	24,096
37	5,200	83	14,400	129	24,527
38	5,400	84	14,600	130	25,000
39	5,600	85	14,800	131	25,500
40	5,800	86	15,000	132	26,000
41	6,000	87	15,200	133	26,500
42	6,200	88	15,400	134	27,000
43	6,400	89	15,600	135	27,500
44	6,600	90	15,800	136	28,000
45	6,800	91	16,000	137	28,500
46	7,000	92	16,200	138	29,000

The second class of models are those that employ parameterized convection, which includes GCMs and SCMs (Table 2). For RCE_large simulations, GCMs employ a global spherical domain with horizontal and vertical grids similar to their CMIP6 configuration (Table 1; Text S1). If GCMs perform RCE_small, they do so with the SCM version of the parent GCM (see CAM5‐GCM, CAM6‐GCM, CNRM‐CM6‐1, GEOS‐GCM, and UKMO‐GA7.1). The SCM simulations are comparable to the CRM RCE_small simulations because ∼100 km is a typical GCM grid size. The simulations are performed for at least 1,000 days, except for IPSL‐CM6, which was limited to 630 days.

One exception to the GCM configuration is WRF‐GCM, which employs 50 km horizontal grid spacing and GCM column physics (including a convective parameterization) but on the Cartesian geometry used for CRM simulations, rather than the global sphere. This configuration is intended to bridge the gap between the limited area CRM setup and the global GCM setup. Six sets of WRF‐GCM simulations are performed, one with no convective parameterization and five each with a different convective parameterization (see Text S1). The same version of WRF is also used to perform CRM simulations with 3 km grid spacing and explicit convection (WRF‐COL‐CRM).

With the exception of the RCE_small configuration for models with parameterized convection, which uses one‐dimensional SCMs, all the RCEMIP simulations are three dimensional. Two‐dimensional simulations are much more computationally efficient and thus have been used in many past RCE studies of tropical convection (e.g., Islam et al., 1993; Grabowski et al., 1996; Nakajima & Matsuno, 1988; Sui et al., 1994; Randall et al., 1994). Two‐dimensional simulations of RCE have been found to feature self‐aggregation (Brenowitz et al., 2018; Held et al., 1993; Grabowski & Moncrieff, 2001, 2002; Jeevanjee & Romps, 2013; Seidel & Yang, 2020; Stephens et al., 2008; van den Heever et al., 2011; Yang, 2018a, 2018b). However, RCEMIP focuses on three‐dimensional simulations in order to compare CRMs and global models and to be inclusive in the models that may participate (many models cannot be easily configured in two dimensions). The dimensionality of the simulation is another factor that could affect the simulated RCE state, which could be considered in a future extension of RCEMIP using a subset of the models.

The hierarchy of models included in RCEMIP offers an opportunity to assess the robustness of the simulated RCE state. In addition to examining the entire ensemble and subsetting by model type (i.e., explicit vs. parameterized) and domain configuration (RCE_small vs. RCE_large), there is the opportunity for more targeted comparisons. For example, the set of WRF‐GCM simulations allows the impact of different deep convective parameterizations to be analyzed. Comparing CAM5‐GCM, CAM6‐GCM, SAM0‐UNICON, SP‐CAM, and SPX‐CAM can reveal how the representation of convection affects the RCE state across the same parent model. CAM5‐GCM and CAM6‐GCM differ in most of their physics packages but have the same deep convective parameterization with similar parameter settings. SAM is used to perform GCRM, CRM, and LES experiments, which allows for the same modeling system to be examined across different geometries and resolutions. Similarly, ICON is used to perform global simulations in its GCM configuration, CRM configurations using two different sets of physics packages, and an LES configuration. UKMO‐CASIM and UKMO‐RA1‐T differ in the microphysics scheme used, while UKMO‐RA1‐T and UKMO‐RA1‐T‐nocloud differ in that the cloud scheme is disabled in the latter. DALES‐damping is identical to DALES except weak damping of the horizontal‐mean horizontal winds to zero is applied (to compensate for the generation of horizontal layers of high winds in the stratosphere due to weak turbulence production). SP‐CAM and SPX‐CAM differ only in how surface enthalpy fluxes are calculated (i.e., on the parent GCM grid or superparameterized by the embedded CRMs). These types of comparisons are useful for determining possible causes of intermodel spread and will be the focus of future studies with the RCEMIP simulations.

## 3 Overview of Simulations

**Figure 1:**
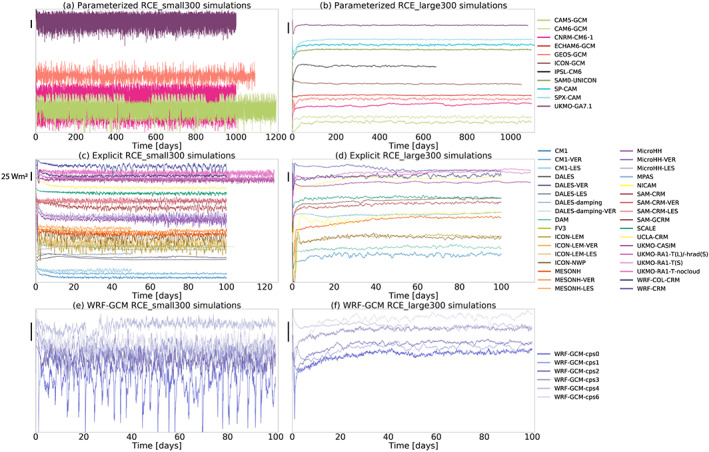
Time series of domain‐mean outgoing longwave radiation (OLR; W m^−2^) in the RCE_small300 (left column) and RCE_large300 simulations (right column). The top row shows GCM simulations with parameterized convection, with the single‐column version of the model in (a) and the global model in (b). The middle row shows simulations with explicit convection (c, d), including CRM, LES, and GCRM simulations. The bottom row shows the WRF‐GCM simulations with 50 km resolution and parameterized convection. The black vertical line to the left of each plot is a 25 W m^−2^ scale bar, but the absolute values of OLR and distance between the curves has no meaning here, as the curves are offset for visual clarity according to OLR + ⟨OLR⟩ + i*x x, where ⟨ ⟩ is the ensemble mean, *i* is the model index (according to alphabetical order), and *xx* = 2 for RCE_large and *xx* = 10 for RCE_small.

First, we provide a general overview of the simulations by examining the hourly averaged outgoing longwave radiation (OLR) in the RCE_small300, RCE_large300, RCE_small_vert300, and RCE_small_les300 simulations. The evolution to RCE takes many tens of days (Tompkins & Craig, 1998b; Cronin & Emanuel, 2013), as shown by the domain‐mean OLR time series in Figure 1. The temporal evolution is similar in all models and emphasizes that RCE is a state of stationarity; that is, there is temporal variability but the statistics are invariant to time. The RCE_small simulations (Figures 1a, 1c, and 1e), especially the single‐column simulations in Figure 1a, have more temporal variability than their RCE_large counterparts (Figures 1b, 1d, and 1f). Based on Figure 1, we compute temporal averages to represent the RCE state (section 4) by averaging over time and neglecting the first 75 days of simulation (except for RCE_small_les, for which the average is taken over Days 25–50). To examine the distribution of convection after equilibrium is reached, we examine the spatial structure of OLR at Day 80 (Day 50 for RCE_small_les). Equivalent figures for precipitable water (PW) and animations of OLR and PW are included in the supporting information (Figures S1–S5).

**Figure 2:**
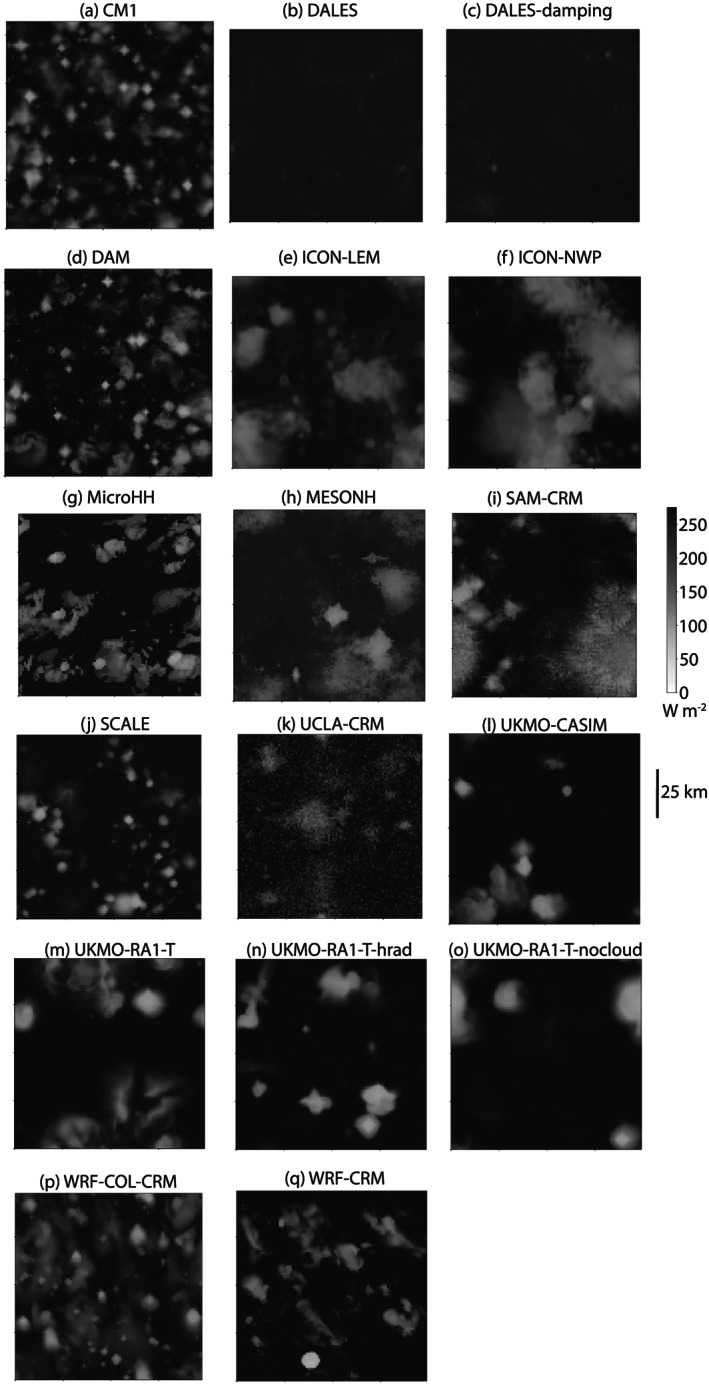
Hourly‐averaged outgoing longwave radiation (W m^−2^) at Day 80 of the RCE_small300 simulation for all cloud‐resolving models. Each panel displays a different model and the size of each panel represents the domain size, which varies slightly across models.

**Figure 3:**
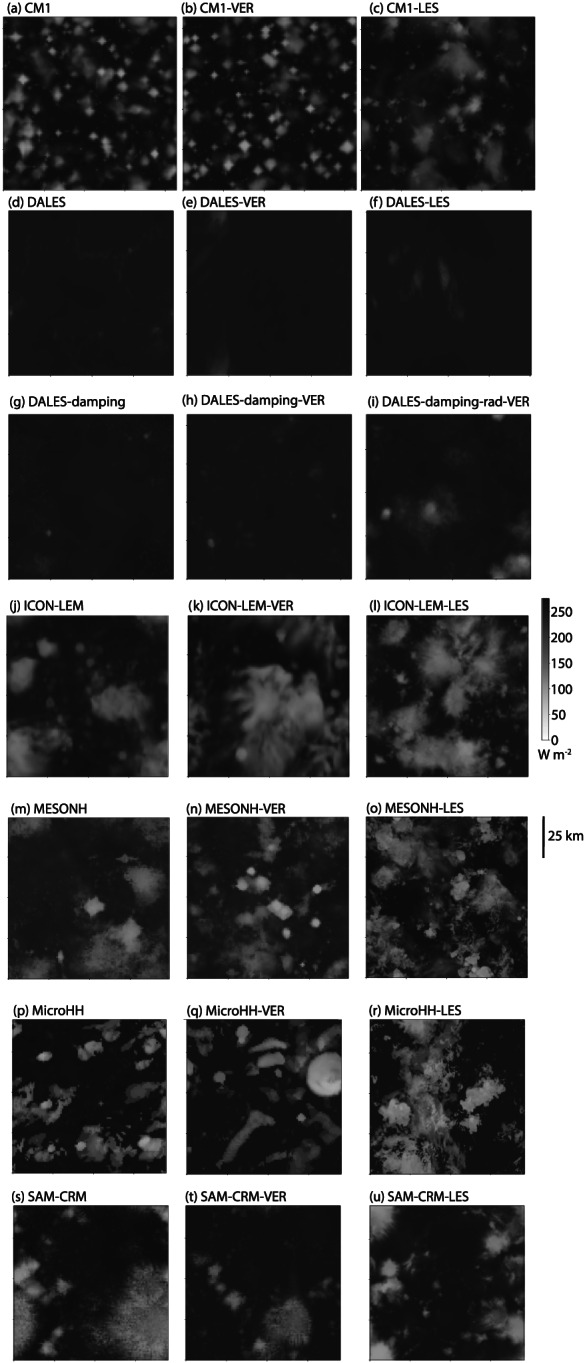
Hourly averaged outgoing longwave radiation (W m^−2^) at Day 80 of the RCE_small300 (a, d, g, j, m, p, s) and RCE_small_vert300 (b, e, h, k, n, q, t) simulations and Day 50 of the RCE_small_les300 (c, f, l, o, r, u) simulations for CM1, DALES, DALES‐damping, ICON_LEM, MESONH, MicroHH, and SAM. The size of each panel represents the domain size, which varies slightly across models. DALES and DALES‐damping have larger values of OLR because the radiative properties of ice clouds were erroneously configured. DALES‐damping‐rad is a corrected version, shown for reference (note that it is the RCE_small_vert300 simulation that is shown in panel (i) despite it being in the column with LES simulations).

Figure 2 shows OLR at Day 80 in the RCE_small300 simulation for all the CRMs. The cloud distribution, as indicated by the white color shading representing cold cloud tops, varies across models. We note that DALES and DALES‐damping have larger values of OLR because the radiative properties of ice clouds were erroneously configured; the grid‐box cloud fraction in the radiation scheme was set to one in the presence of liquid clouds but not ice clouds. If this is corrected, the OLR distribution looks more realistic (see the RCE_small_vert300 simulation for DALES‐damping‐rad in Figure 3). While the RCE state is affected by this error, the sensitivity to SST is similar. The OLR and PW snapshots (Figure S1) and animations indicate that, in all models except UKMO‐RA1‐T, convection is quasi‐randomly distributed in space and time with nearly spatially uniform PW, reflecting unaggregated convection in the small domain. While ICON‐NWP appears slightly aggregated toward the beginning of the simulations at 300 and 305 K, UKMO‐RA1‐T is the only model that exhibits consistent convective aggregation in RCE_small. This suggests that the minimum domain size required for self‐aggregation (Muller & Held, 2012) is model dependent, since self‐aggregation was not expected in the RCE_small ∼100 × ∼100 km^2^ domain. Since part of the objective of the RCE_small simulations is to provide an unaggregated control to compare to, an additional set of RCE_small simulations was performed with UKMO‐RA1‐T in which the radiative heating rates were spatially homogenized (UKMO‐RA1‐T‐hrad). This prevents aggregation, and it is these simulations from which the UKMO‐RA1‐T RCE_large simulations are initialized. Figure 3 shows OLR in the RCE_small300, RCE_small_vert300, and RCE_small_les300 simulations for the six models that performed them (CM1, DALES, ICON‐LEM, MESONH, MicroHH, and SAM). All simulations are unaggregated, and the LES simulations have finer spatial structures, but otherwise, there are no obvious differences compared to the RCE_smallsimulations (see also Figure S2 and animations).

**Figure 4:**
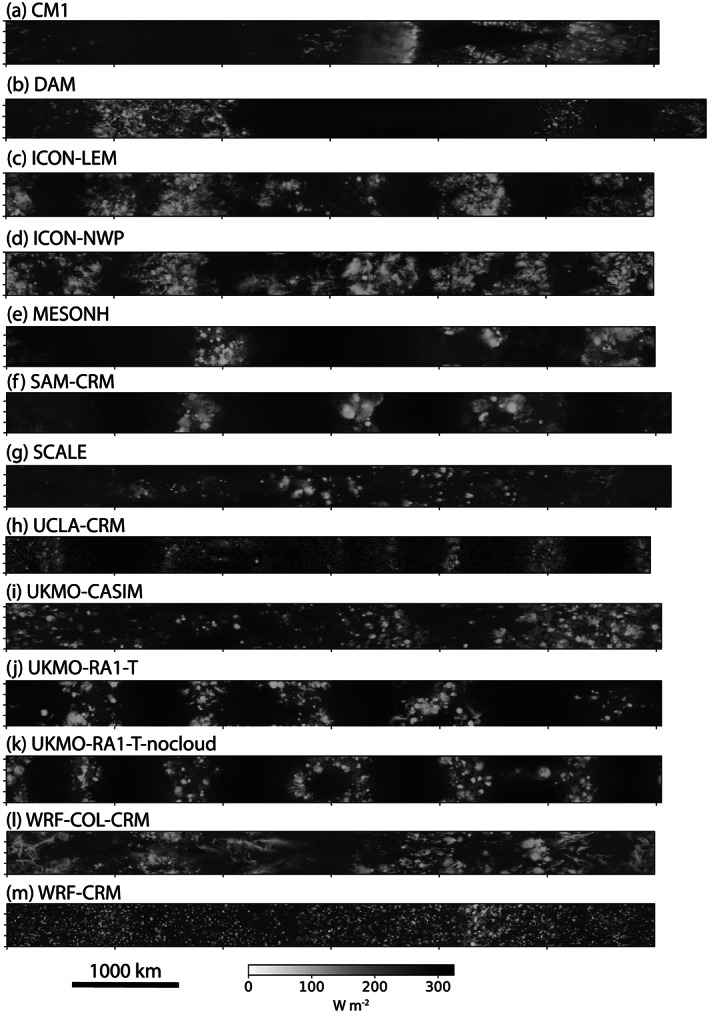
Hourly averaged outgoing longwave radiation (W m^−2^) at Day 80 of the RCE_large300 simulation for all cloud‐resolving models. Each panel displays a different model and the size of each panel represents the domain size, which varies slightly across models. Note that FV3 is missing from the figure because outgoing longwave radiation was only reported as daily averages.

Figure 4 shows OLR at Day 80 in the RCE_large300 simulation for all the CRMs. Except for WRF‐CRM, the condensate field simulated by all of the models shows evidence of large‐scale clustering or aggregation. The way in which the condensate is clustered, however, differs. Differences are evident in the number of aggregated regions, their spatial scale, and their orientation. This clustering is also evident in the distribution of PW, which varies in association with the condensate field (Figure S3), in contrast to the RCE_small simulations (Figures S1 and S2). Animations and *y*‐averaged Hovmöller diagrams reveal a rich spectrum of variability, including the growth and decay of individual convective cells within the aggregated envelope, propagation of gravity waves, sloshing of the convection along the *x* direction, mergers and splitting of convective bands, and expansion and contraction of dry and clear air regions (Figure S6). Some models appear visually to be more aggregated than others (e.g., UKMO‐RA‐1‐T appears more aggregated than UKMO‐CASIM); the degree of aggregation will be quantified in section 5.

**Figure 5:**
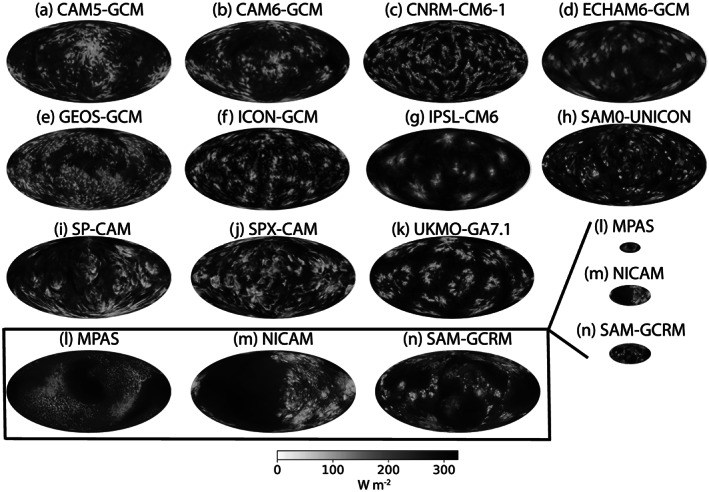
Hourly averaged outgoing longwave radiation (W m^−2^) at Day 80 of the RCE_large300 simulation for all global models (except for IPSL‐CM6, which reported daily averaged output). All models shown are GCMs with parameterized convection (panels a–k) except MPAS, NICAM, and SAM (panels l–n), which are global cloud‐resolving models that employ reduced Earth radius of *R*_*E*_/8, *R*_*E*_/4, and *R*_*E*_/4, respectively, and are shown to scale and, in the box, zoomed in.

Figure 5 shows OLR in the RCE_large300 simulation at Day 80 for all the global model (GCM and GCRM) simulations. Each GCRM is shown to scale based on its reduced Earth radius, along with an additional zoomed‐in view. The supporting information contains a figure that shows OLR in a subset of the global domains that is approximately the size of the CRM domain, for comparison (Figure S9). The global simulations all appear aggregated to some extent, but there is diversity in the spatial structure of convection; some simulations have one or two hemisphere‐scale aggregated regions (e.g., NICAM and CAM5), some have quasi‐regularly spaced aggregated clusters (e.g., UKMO‐GA7.1, ECHAM, ICON‐GCM, and IPSL‐CM6), some have convection organized along irregular lines (e.g., CNRM‐CM6‐1), and others seem only partially aggregated, with only a few dry, clear patches amidst more uniform convection (e.g., CAM6‐GCM, GEOS‐GCM, and MPAS). These differences are also reflected in the distribution of PW (Figure S4). Animations indicate that the convective patterns are approximately stationary in most of the models, with the cloud field as represented by OLR varying more rapidly than the PW field (see also Figure S7). SPX‐CAM, SAM0‐UNICON, and CAM6‐GCM have more temporal variability in the convective regions than other models, and the convective clusters in MPAS seem to propagate around a large central dry patch. The degree of aggregation is quantified in section 5, and discussion of the qualitative sensitivity of the patterns of aggregation and their temporal variability to SST is provided in section 6.2.

**Figure 6:**
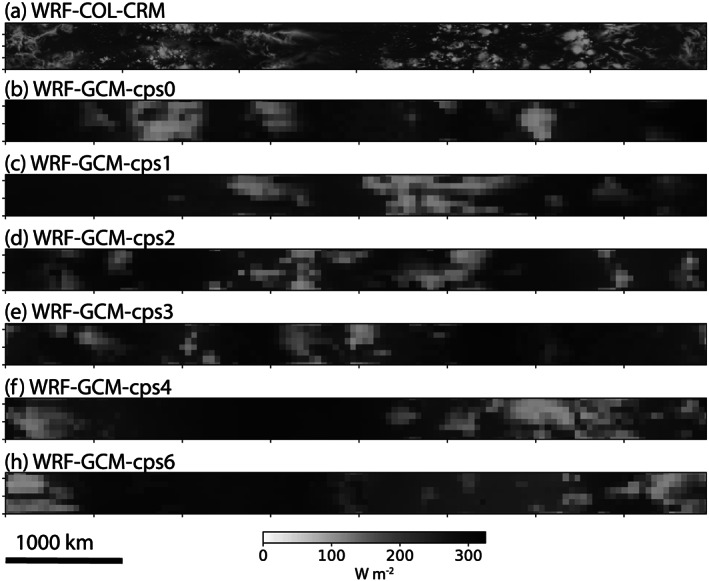
Hourly averaged outgoing longwave radiation (W m^−2^) at Day 80 of the RCE_large300 simulation for all versions of WRF 3.5.1. Panel (a) displays the same WRF‐COL‐CRM configuration as in Figure 4l. The other panels (b–h) show WRF‐GCM in the Cartesian RCE_large300 configuration but with 50 km grid spacing and convective parameterizations.

Figure 6 shows OLR at Day 80 in the RCE_large300 simulation in the set of WRF simulations that include a CRM configuration (WRF‐COL‐CRM) and a GCM‐like configuration in Cartesian geometry (WRF‐GCM). In addition to the obvious difference in fine‐scale structures between the 3 and 50 km resolutions, for the same parent model, the GCM versions seem more aggregated than the CRM version (WRF‐COL‐CRM). Among the different WRF‐GCM versions, there are no obvious differences in the scale or degree of aggregation (see also PW in Figure S5). We note that WRF‐COL‐CRM becomes slightly more aggregated later in the simulation with more persistent dry regions (Movie S27). The only model in the overall RCEMIP ensemble that does not aggregate, WRF‐CRM, is a newer version of WRF than WRF‐COL‐CRM that employs different radiation and microphysics schemes.

In summary, while the spatial patterns of convection are diverse, all but one (UKMO‐RA1‐T) of the RCE_small simulations appear unaggregated, and all but one (WRF‐CRM) of the RCE_large simulations appear aggregated, to varying extents. The emergence of aggregation is independent of the representation of convection (i.e., explicit vs. parameterized). The next section will analyze whether the diverse spatial patterns lead to similar or different domain‐mean characteristics of the RCE state.

## 4 The RCE State

In this section, we characterize the RCE state across the RCEMIP ensemble by considering domain‐ and time‐average quantities, neglecting the first 75 days. We focus on temperature, humidity, clouds, and quantities related to the mean top‐of‐atmosphere energy budget. We discuss the results in RCE_small and RCE_large separately; the difference between pairs of RCE_small and RCE_large simulations is discussed in section 5. We focus on the simulations at 300 K, as representative of the current tropical climate. Results for the simulations at 295 and 305 K can be found in the supporting information (Figures S11–S18).

### 4.1 Temperature

**Figure 7:**
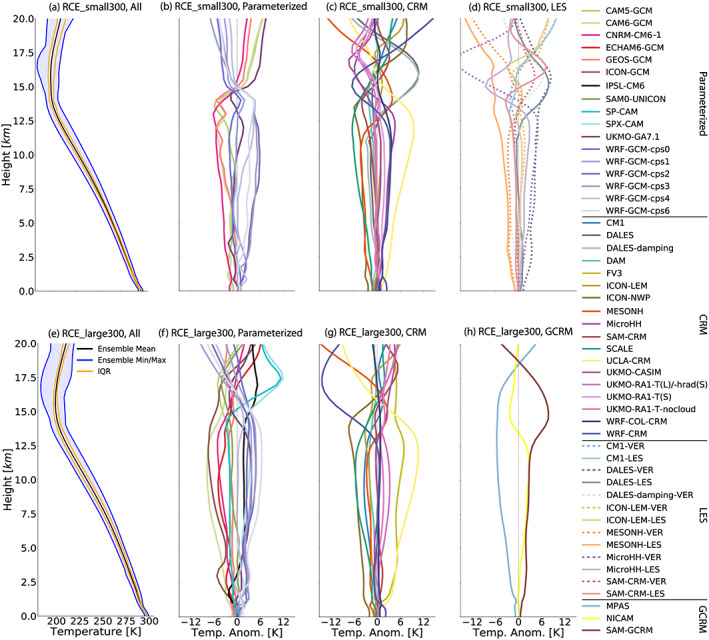
Horizontal‐mean temperature profile, averaged in time excluding the first 75 days of simulation of the RCE_small (top row: a–d) and RCE_large (bottom row: e–h) simulations at 300 K. The first column (a, e) includes all models that performed each type of simulation, where the black line is the ensemble mean, the blue shading shows the range across all models, and the orange lines indicate the interquartile range (IQR). The other columns display the temperature anomaly in each subgroup of models as an anomaly from the ensemble mean of that subgroup, for models with parameterized convection (second column: b, f), CRMs (third column: c, g), models that performed RCE_small_vert (dashed) and RCE_small_les (solid) simulations (panel d; RCE_small_les simulations are averaged over Days 25–50), and GCRMs (panel h).

The domain‐ and time‐averaged temperature profiles are shown in Figure 7, in which the first column shows the ensemble mean and spread across all models while the other columns display the temperature anomaly in each subgroup of models as an anomaly from the ensemble mean of that subgroup. The intermodel spread across all models is similar in the RCE_small and RCE_large simulations, considering the ensemble range and the interquartile range (Figures 7a and 7e). We expect that the troposphere in RCE should evolve toward a roughly moist adiabatic temperature profile. While the average tropospheric lapse rate (averaged between the surface and the radiative tropopause) in the RCE_small simulations is 7.5 K km^−1^ (Figure 11), the temperature profiles are systematically several degrees cooler than a moist adiabatic profile (not shown). This is consistent with theory that indicates that tropical temperature profiles are set by *dilute* moist adiabats in which entrainment systematically reduces cloud updraft moist static energy (Seeley & Romps, 2015; Singh & O'Gorman, 2013). The amount of dilution depends on entrainment rate and precipitation efficiency (Romps, 2016), which may explain the spread in temperature profiles across the RCEMIP simulations. In fact, preliminary analysis suggests that there is a larger deviation from a moist adiabat (more instability) in simulations that are on average moister in the midtroposphere (not shown). An initial calculation indicates that this is qualitatively consistent with expectations from the simple plume model of Romps (2016) in which both instability and relative humidity depend on entrainment (see also Romps, 2014; Singh et al., 2019), though this relationship could be complicated by changes in precipitation efficiency across models. The cold point occurs at different heights and at different temperatures across the models but, in the RCE_small300 simulations, is on average 193 K and occurs at an average height of 15.7 km (the first and third quartiles are 190.4 and 195.3 K for the temperature and 14.8 and 16.2 km for the height). The radiative tropopause, defined as the first level at which the radiative cooling rate intersects zero, is on average below the cold point, with an average temperature of 205 K (interquartile range of 9.5 K) and an average height of 12.7 km (interquartile range of 1.3 km). The average cold point in RCE_large300 is 197 K at an average height of 16.2 km, and the average radiative tropopause is 205 K at an average height of 15.3 km. While the temperature profile has the same general shape in all models, when considering the temperature anomalies from the ensemble mean, the range in temperatures at a given height can be up to 10 K (Figure 7). UCLA_CRM is notably warmer in the troposphere than the other simulations in both RCE_small and RCE_large (see yellow line to the right of the ensemble of profiles in Figures 7c and 7g). There are also large temperature differences in the lower stratosphere (∼17–20 km), which is somewhat surprising given that the RCEMIP protocol enforces the same trace gas profiles and insolation.

Across all simulations, including all SSTs, the average difference between the temperature at the lowest model level and the SST is −2.4 K, though some of the spread (the interquartile range is 1.0 K) is due to the lowest model level being placed at different heights in different models. This air‐sea temperature difference is influenced by the surface sensible heat flux, which is on average 9.4 W m^−2^ in RCE_small300 (Table  A1) and 11.2 W m^−2^ in RCE_large300 (Table A2). For those models that report 2 m air temperature, the difference between that and SST is on average −1.7 K, with an interquartile range across models of 0.8 K. The differences reflect differences in boundary layer and surface schemes, including how 2 m air temperatures are estimated.

### 4.2 Relative Humidity

**Figure 8:**
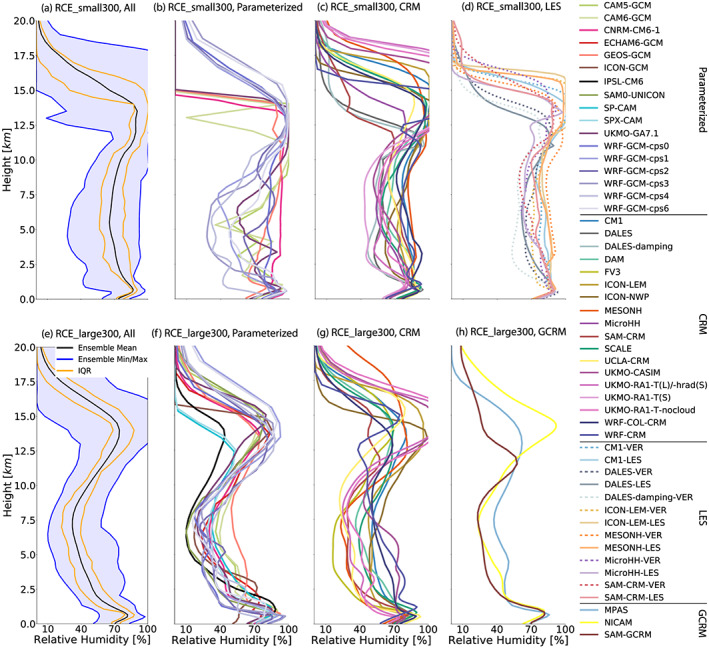
Horizontal‐mean relative humidity profile, averaged in time excluding the first 75 days of simulation of the RCE_small (top row a–d) and RCE_large (bottom row: e–h) simulations at 300 K. The first column (a, e) includes all models that performed each type of simulation, where the black line is the ensemble mean, the blue shading shows the range across all models, and the orange lines indicate the interquartile range (IQR). The other columns display each subgroup of models: models with parameterized convection (second column: b, f), CRMs (third column: c, g), models that performed RCE_small_vert (dashed) and RCE_small_les (solid) simulations (panel d; RCE_small_les simulations are averaged over Days 25–50), and GCRMs (panel h).

Relative humidity is calculated based on each model's formulation for saturation over water and ice. Thus, different formulations may be used in different models, but each formulation is consistent with how that model's clouds respond to and regulate relative humidity. Several models inadvertently reported relative humidity with respect to saturation over water at all temperatures. To ensure a representative comparison, we corrected these calculations to relative humidity with respect to saturation over ice at temperatures below freezing using the Wagner and Pruß (2002) and Wagner et al. (2011) formulations. There is a large spread in simulated relative humidity across the RCEMIP ensemble. In the RCE_small simulations, relative humidities in the midtroposphere vary between ∼25% and ∼90% (Figure 8a), with an average minimum relative humidity between 2 and 10 km of ∼61%. Relative humidity near the surface varies from ∼60% to more than ∼80% with an average of ∼73%, but this does not explain the spread in the free troposphere (i.e., if the relative humidity profiles are shifted such that all models start from the same relative humidity value at the lowest model level, the intermodel spread in the free troposphere is actually increased). The large spread in simulated relative humidity may be a result of its control by detrainment (Romps, 2014; Singh et al., 2019), and/or precipitation efficiency and downdrafts (Emanuel, 2019), processes that are likely represented differently across the RCEMIP ensemble. Many of the models are near saturation or supersaturated with respect to ice near the tropopause; this behavior is expected. The LES models also exhibit a large spread in relative humidity in the free troposphere (Figure 8d), but it is smaller than the spread across the same six models in RCE_small300. Half of the LES models have a moister midtroposphere than their RCE_small300 counterparts while half have a drier midtroposphere; the average magnitude of the difference is 4.4.

While there is still a large spread across the models in the RCE_large simulations, there is a more consistent shape to the relative humidity profile with a pronounced mid‐level minimum in all models and overall better model agreement (smaller standard deviation and interquartile range, relative to the mean values) than in RCE_small (Figure 8e). The models with parameterized convection are in closer agreement than the CRM and GCRM simulations (cf. Figures 8f, and 8g and 8h). ICON_GCM (Figure 8f) is an outlier that is unusually dry in the boundary layer compared to the other models; this is also found in realistic AMIP‐style simulations with ICON and is suspected to be a bug. IPSL‐CM6 (Figure 8f) is also an outlier with generally lower relative humidity. The spread across models in RCE_large is not correlated with the spread across those same models in RCE_small, suggesting that the spread in RCE_large may reflect differences in aggregation moreso than differences in the baseline RCE state.

### 4.3 Clouds

**Figure 9:**
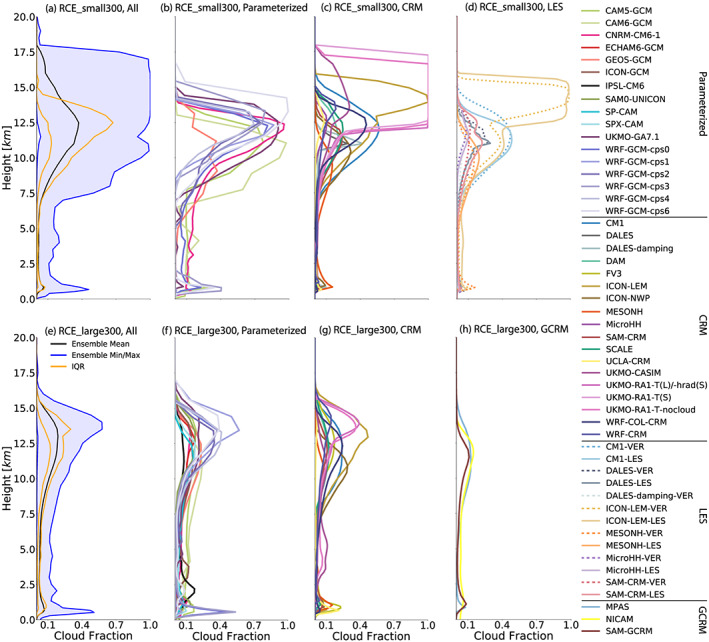
Domain‐wide cloud fraction profile, averaged in time excluding the first 75 days of simulation of the RCE_small (top row: a–d) and RCE_large (bottom row: e–h) simulations at 300 K. The first column (a, e) includes all models that performed each type of simulation, where the black line is the ensemble mean, the blue shading shows the range across all models, and the orange lines indicate the interquartile range (IQR). The other columns display each subgroup of models: models with parameterized convection (second column: b, f), CRMs (third column: c, g), models that performed RCE_small_vert (dashed) and RCE_small_les (solid) simulations (panel d; RCE_small_les simulations are averaged over Days 25–50), and GCRMs (panel h).

As with relative humidity, the RCEMIP ensemble exhibits large variability in cloud fraction profiles in the RCE_small simulations (Figures 9a–9d; Table S2), with the peak high (“anvil”) cloud fraction varying an order of magnitude from a low value of ∼0.08 in UCLA‐CRM to 1.0 in several of versions of the UKMO cloud‐resolving model. A cloud fraction of 1.0 at a particular model level indicates that the entire domain is covered in cloud at that level. In this study, a cloud is defined according to a threshold value of cloud condensate (10^−5^ kg kg^−1^ or 1% of the saturation mixing ratio over water, whichever is smaller) or the output of a cloud scheme (if utilized by a given model). The ICON_LEM simulations also exhibit high cloud fractions very close to 1.0, which stems from clouds that are very optically thin (due to the settings in the microphysics scheme, in which the threshold value for self‐collection is set at 10^−6^ for ice and there is a small sedimentation velocity of ice particles). If an alternate threshold for identifying a cloud is used (e.g., cloud condensate must be larger than 5 × 10^−7^ kg kg^−1^), the ICON_LEM cloud fractions are more in line with the other models. In addition to simulating very different amounts of high cloud, there is also a large spread in the height at which the anvil cloud peak occurs (∼9–17 km; Table S4). The intermodel spread in cloud fraction does not collapse when plotted against temperature as a vertical axis, indicating that the models have anvil cloud peaks at different heights because they form them at different temperatures (the interquartile range of anvil cloud temperature is 11.9 K; Table S3). With the exception of CNRM‐CM6‐1, CAM5, and CAM6 (the single‐column versions of GCMs with parameterized convection), all models have very small amounts of mid‐level cloud (∼3–8 km). There is also variability in the amount of low cloud, though generally less variability in the models with explicit convection (Figures 9c and 9d). However, it should be noted that because of the absence of strong subsiding motions, which in nature are generally forced by horizontal heterogeneities in surface boundary conditions, the RCE setup is not favorable to certain tropical low‐cloud regimes such as stratocumulus. MESONH is an outlier among the CRMs, with approximately twice the amount of low cloud than the other models (Figure 9c). The low cloud amount exhibits some sensitivity to resolution, though not always consistently across models (compare curves in Figures 9c and 9d). For the six models that performed RCE_small, RCE_small_vert, and RCE_small_les simulations, low cloud amount increases with an increasing number of vertical levels in ICON_LEM and DALES, decreases in DALES‐damping, and has no change in the other models. Low cloud amount decreases with decreasing horizontal grid spacing in all models except CM1 and MicroHH, for which there is no change.

There is also substantial spread in cloud fraction profiles in the RCE_large simulations (Figures 9e–9h; Table S5), but the high cloud fraction spans a narrower range than in the RCE_small simulations. UCLA‐CRM has notably fewer high clouds than the other CRMs (Figure 9g), and IPSL‐CM6 stands out as an outlier with few high clouds compared to the other models with parameterized convection (Figure 9f). If WRF‐GCM is excluded, there is a suggestion that models with parameterized convection (Figure 9f) have better agreement of anvil cloud amount and exhibit cloudiness that is more uniform throughout the column than the CRMs (Figure 9g). CRMs have more notable anvil and low cloud peaks, consistent with CRMs having fewer mid‐level clouds. WRF‐GCM‐cps0 and WRF‐GCM‐cps3 are outliers that have much higher low cloud fraction than other models with parameterized convection (Figure 9f). In general, there are fewer high clouds and more mid‐level clouds in the RCE_large simulations than in the RCE_small simulations (Figure 9e cf. Figure 9a).

**Figure 10:**
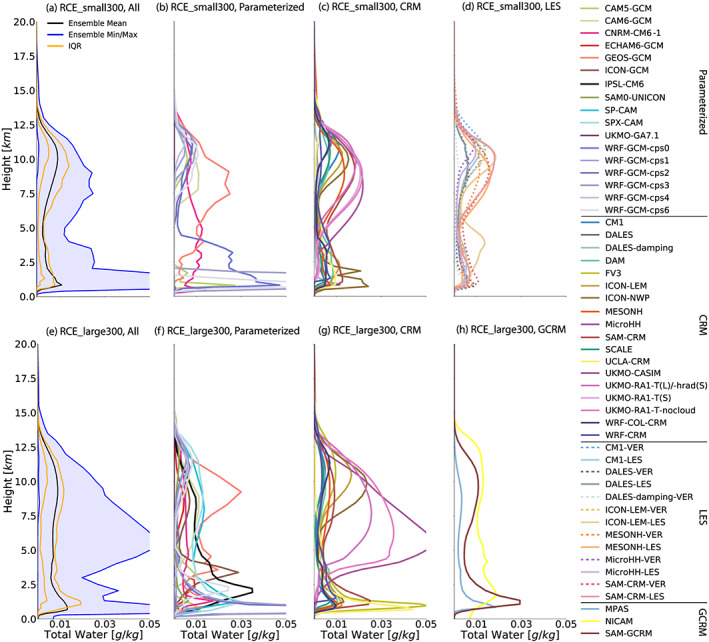
Horizontal‐mean total cloud water condensate profile, averaged in time excluding the first 75 days of simulation of the RCE_small (top row: a–d) and RCE_large (bottom row: e–h) simulations at 300 K. The first column (a, e) includes all models that performed each type of simulation, where the black line is the ensemble mean, the blue shading shows the range across all models, and the orange lines indicate the interquartile range (IQR). The other columns display each subgroup of models: models with parameterized convection (second column: b, f), CRMs (third column: c, g), models that performed RCE_small_vert (dashed) and RCE_small_les (solid) simulations (panel d; RCE_small_les simulations are averaged over Days 25–50), and GCRMs (panel h).

Cloud fraction depends sensitively on the semiarbitrary threshold used to identify clouds. Therefore, we also examine the horizontal‐mean profiles of total cloud water condensate, which is primarily liquid at low levels and ice at high levels (Figure 10). Consistent with the spread in cloud fraction profiles (Figure 9a), the total cloud water profiles in the RCE_small simulations (Figure 10a) exhibit widely varying amounts of cloud water (by an order of magnitude or more) and differ both in the amount of upper level cloud condensate and the height at which the anvil cloud condensate peak occurs (Figures 10b–10d). The total cloud water profiles vary smoothly with height in the CRM RCE_small simulations (Figure 10c). Among the models with explicit convection (Figures 10c and 10d), the models differ in whether the maximum of the cloud water profile occurs at low altitudes or at high altitudes. For example, the maximum of the cloud water profile of the UKMO family of models is in the upper troposphere whereas the maximum of the cloud water profile for UCLA‐CRM, MPAS, and DAM is in the lower troposphere. Some models, like ICON‐LEM, SAM‐CRM, and WRF‐COL‐CRM, have similar peaks of cloud water in the upper and lower troposphere (Figure 10c). Finer grid spacing does not reduce the spread; among models that performed both CRM and LES simulations, the LES simulations differ as much as their counterparts at coarser resolution (Figure 10d).

The total cloud water profiles appear to have a wider spread in the RCE_large simulations (Figure 10e), with more disagreement in the amount of cloud water than in the RCE_small simulations, but this impression may be a result of the behavior of individual models (i.e., UKMO family in Figure 10g). Models with parameterized convection place their cloud water peaks at very different heights and many models lack a distinct upper level peak (Figure 10f).

The differences in simulated cloudiness in the RCE_small simulations reflect fundamental differences in how the different models handle clouds and in the equilibrium state that each model converges to. The differences in simulated cloudiness in the RCE_large simulations reflect these differences as well as differences in the degree of aggregation simulated by each model and differences in how each model represents the response of cloudiness to self‐aggregation. This will be explored further in later sections.

**Figure 11:**
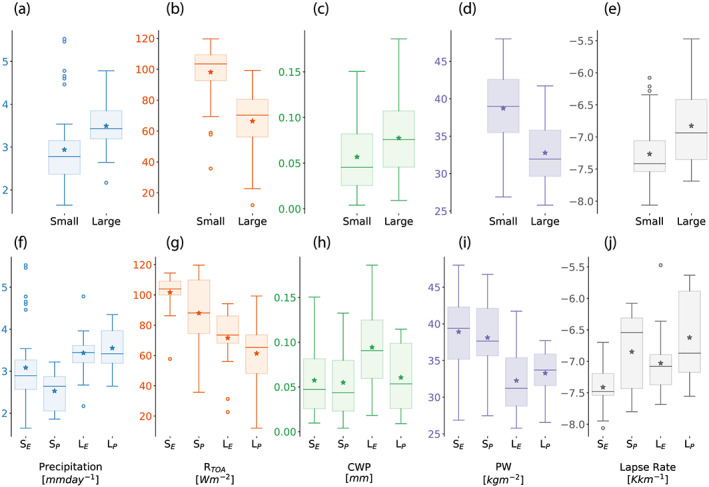
Box and whiskers plots of domain‐average quantities, averaged in time excluding the first 75 days of the RCE_small300 and RCE_large300 simulations. RCE_small_vert300 and RCE_small_les300 simulations are included in the “Small” statistics. The top row (panels a–e) includes all models in the statistics while the bottom row (panels f–j) splits the models into those with explicit (“S_*E*_” and “L_*E*_”) and parameterized convection (“S_*P*_” and “L_*P*_”), where the “S” and “L” indicate small and large simulations, respectively. The variables shown are precipitation rate (mm day^−1^; panels a and f), net radiation at the top of atmosphere (*R*_*TOA*_; W m^−2^, downward defined as positive; panels b and g), condensed water path (CWP; mm; panels c and h), precipitable water (PW; kg m^−2^; panels d and i), and the tropospheric lapse rate (K km^−1^; panels e and j). The asterisk indicates the multimodel mean, the horizontal line the median, the shaded region the interquartile range, and the circles the outliers. The whiskers are defined as 1.5 times the interquartile range; this does not extend beyond the range of the data.

### 4.4 Energy Budget and Hydrological Cycle

Appendix A1 provides a summary of domain‐average statistics in each of the simulations at 300 K, including variables related to the energy budget and hydrological cycle. The intermodel spread in a subset of those variables is summarized in Figure 11, which includes domain‐average precipitation rate, net radiation at the top of atmosphere (R_*TOA*_), condensed water path, PW, and tropospheric lapse rate. R_*TOA*_ is calculated as the difference between the net absorbed solar radiation at the top of the atmosphere and the OLR (*ASR*_*TOA*_−*OLR*). R_*TOA*_ is positive, representing a net downward radiative flux, or a net energy gain for the climate system. The condensed water path is calculated as the sum of the cloud ice water path and the cloud liquid water path. The tropospheric lapse rate is calculated as *dT*/*dz* averaged over the troposphere, where the top of the troposphere is defined as the height at which the radiative cooling rate first intersects zero.

There are several outliers indicated in Figure 11, defined as points beyond 1.5 times the interquartile range. For precipitation, DALES (4.8 mm day^−1^) and DALES‐damping (4.6 mm day^−1^) are outliers from RCE_small300; DALES (5.5 mm day^−1^), DALES‐damping (4.7 mm day^−1^), and MicroHH (4.5 mm day^−1^) are outliers from RCE_small_vert300; and DALES (5.5 mm day^−1^) is an outlier from RCE_small_les300 (Figure 11a). ICON‐NWP‐CRM (2.2 mm day^−1^) is the outlier for precipitation in the large simulation. For R_*TOA*_, CNRM‐CM6‐1 (58.88 W m^−2^), WRF‐CRM (57.70 W m^−2^), and WRF‐GCM‐cps3 (35.68 W m^−2^) are the outliers in the small simulation in Figure 11b; WRF‐GCM‐cps3 (12.00 W m^−2^) is the outlier in the large domain simulation. For lapse rate, WRF‐GCM‐cps2 (−6.08 K km^−1^), WRF‐GCM‐cps4 (−6.28 K km^−1^), and WRF‐GCM‐cps6 (−6.21 K km^−1^) are the outliers in the small simulation (Figure 11e). Overall, there are more outliers in Figures 11a–11e in the RCE_small simulations, contributed by the single‐column versions of models with parameterized convection, the very small WRF‐GCM domain, and configurations of DALES and MicroHH. It is notable that all lapse rate outliers are contributed from WRF‐GCM.

There is substantial intermodel spread in all quantities, but tropospheric lapse rate exhibits the best agreement, as measured by the interquartile range relative to the mean value (Figure 11e). For some variables (i.e., net radiation and lapse rate), the spread in the RCE_large simulations is larger than that in the RCE_small simulations, but for other variables the spread is larger in RCE_small or similar between the two configurations. It is therefore difficult to determine whether the intermodel spread in the representation of the RCE state is due to differing degrees of aggregation or to more fundamental differences in model physics and numerics. Furthermore, there is no consistency in whether the intermodel spread is larger in models with explicit or parameterized convection; it is larger in models with explicit convection for precipitation, condensed water path, and PW, but larger in models with parameterized convection for net radiation and lapse rate (Figures 11f–11j).

## 5 Self‐Aggregation

### 5.1 Degree of Self‐Aggregation

There is no single agreed upon quantitative measure of the degree of aggregation (Wing, 2019); therefore, here we quantify the degree of aggregation using three different metrics: the organization index (*I*_*org*_; Tompkins & Semie, 2017), subsidence fraction (*f*_*sub*_; Coppin & Bony, 2015), and the spatial variance of column relative humidity ( $\sigma_{CRH}^{2}$ Wing & Cronin, 2016). An alternate metric, the spatial variance of PW scaled by its mean value, is presented in the supporting information (Figures S24 and S25). *I*_*org*_ is a clustering metric that compares the nearest neighbor distribution of deep convective entities to that of a random distribution. *f*_*sub*_ is the area fraction of the domain where the daily‐average large‐scale vertical velocity at 500 hPa is directed downward. Vertical velocity is first averaged in time over a day and in space over ∼100 × ∼100 km^2^ blocks. Column relative humidity (*CRH*) is defined as the ratio of PW to saturated PW (mass weighted vertical integrals of specific humidity and saturation specific humidity, respectively). Its spatial variance ( $\sigma_{CRH}^{2}$) is calculated as the domain mean of the squared anomalies of *CRH* (anomalies taken from the domain mean). $\sigma_{CRH}^{2}$ is not calculated for SCMs. More details about the calculation of each metric can be found in Appendix  B. A simulation that is aggregated is indicated by *f*_*sub*_ greater than 0.5 and *I*_*org*_ greater than 0.5. There is no specific value of $\sigma_{CRH}^{2}$ that indicates aggregated as opposed to unaggregated conditions; this metric should instead be interpreted to indicate relative differences in the degree of aggregation. For all three metrics, larger values indicate more aggregated convection.

**Figure 12:**
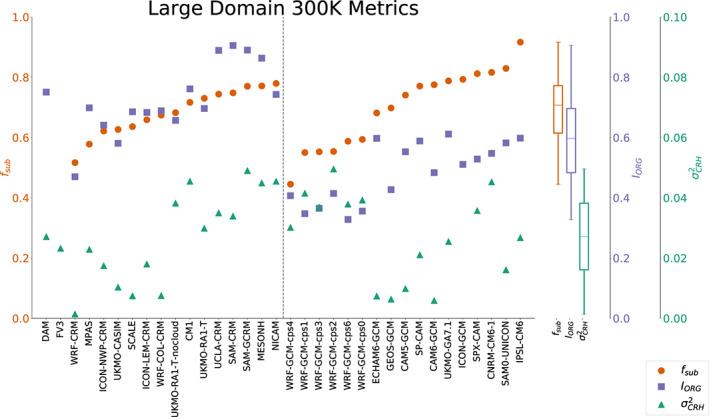
Degree of aggregation in RCE_large300 based on subsidence fraction (red circles), *I*_*org*_ (blue squares), and spatial variance of column relative humidity (green triangles) in all models, averaged in time excluding the first 75 days of simulation. The models are ordered such that the models with explicit convection are to the left of the dashed line and models with parameterized convection are to the right of the dashed line. Within each group of models, they are ordered according to their values of subsidence fraction. The two models for which subsidence fraction could not be computed are listed first. Box plots indicate the spread of each metric across all models.

The degree of aggregation can also be qualitatively estimated by examining the distribution of PW, which has a much wider spread in the RCE_large simulations compared to the RCE_small simulations, indicating that nearly all the RCE_large simulations are aggregated while the RCE_small simulations are generally not (Figure S10). Quantitative estimates of the degree of aggregation in RCE_large300 are shown in Figure 12; similar figures for RCE_large295 and RCE_large305 may be found in the supporting information (Figures S19 and S20). All RCE_large300 simulations have subsidence fractions greater than 0.5 except WRF‐GCM‐cps4 (Figure 12). All RCE_large300 simulations have *I*_*org*_ values greater than 0.5 except WRF‐CRM, CAM6‐GCM, GEOS‐GCM, and the WRF‐GCM simulations. The low *I*_*org*_ values in the WRF‐GCM simulations are an artifact of the coarse grid that does not allow for short distances between clusters of convection; these simulations are aggregated based on the other metrics and visual inspection. CAM6‐GCM and GEOS‐GCM have high *f*_*sub*_ values so are considered to be aggregated, though less so than other models (as mentioned in section 3). WRF‐CRM has a value of *I*_*org*_ that is less than 0.5, a *f*_*sub*_ value that is marginally higher than 0.5 (0.517), and the lowest value of $\sigma_{CRH}^{2}$ among the RCE_large simulations (0.001). This, combined with the visual appearance of convection throughout the domain noted in section 3 and the narrow PW distribution (Figure S10), indicates that WRF‐CRM is not aggregated. The value of $\sigma_{CRH}^{2}$ excluding the unaggregated WRF‐CRM varies between 0.006 and 0.050, all of which are much larger than the values in the unaggregated RCE_small300 simulations of, on average, 0.001.

Despite nearly all simulations being aggregated, the degree of aggregation varies from barely aggregated (*I*_*org*_ values just above 0.5 in CNRM‐CM6‐1, ICON‐GCM, and SPX‐CAM, for example) to strongly aggregated (*I*_*org*_ values near 0.9 in MESONH, SAM‐CRM, SAM‐GCRM, and UCLA‐CRM). The relative spread of $\sigma_{CRH}^{2}$ (measured by its interquartile range divided by its mean) is greater than for *I*_*org*_ or *f*_*sub*_. *I*_*org*_ values are generally higher in the models with explicit convection than in those with parameterized convection (compare the left side of Figure 12 to the right side), but *f*_*sub*_ and $\sigma_{CRH}^{2}$ do not vary consistently in this manner, so it is more likely that *I*_*org*_ is ill‐suited for models with coarse resolution than that models with parameterized convection are actually less aggregated. Within the group of models with explicit convection, the three metrics are correlated, such that models with higher values of *f*_*sub*_ also have higher values of *I*_*org*_ (*r* = 0.84) and $\sigma_{CRH}^{2}$(*r* = 0.83). *I*_*org*_ and $\sigma_{CRH}^{2}$ are also correlated (*r* = 0.74). Within the group of models with parameterized convection, *f*_*sub*_ and *I*_*org*_ are correlated (*r* = 0.81), but $\sigma_{CRH}^{2}$ does not vary similarly. The lack of correlation with $\sigma_{CRH}^{2}$ may be skewed by the set of WRF‐GCM simulations.

### 5.2 Impact on Mean State

**Figure 13:**
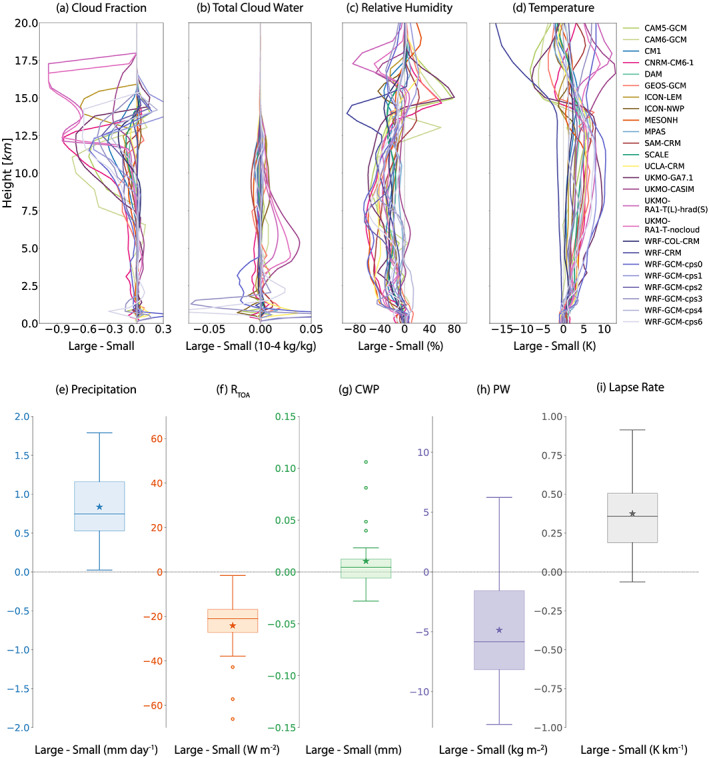
Horizontal and time mean (average excluding the first 75 days) of the difference between pairs of RCE_small300 and RCE_large300 simulations, for cloud fraction (a), total cloud water (b), relative humidity (c), temperature (d), precipitation rate (e), net radiation at the top of the atmosphere (f), condensed water path (g), precipitable water (h), and the tropospheric lapse rate (i). The difference is taken as RCE_large300‐RCE_small300. In the box and whiskers plots (e–i), the asterisk indicates the multimodel mean, the horizontal line the median, the shaded region the interquartile range, and the open circles the outliers. The whiskers are defined as 1.5 times the interquartile range; this does not extend beyond the range of the data.

The occurrence of self‐aggregation has a dramatic impact on the modeled mean state, as could be inferred by comparing the small and large simulations in section 4. An advantage of the RCEMIP protocol is that an explicit comparison between unaggregated and aggregated RCE mean states can be made by taking the difference between pairs of small and large‐domain simulations, for each model and SST. Here we discuss the 300 K simulations as a representative example. While the exact differences between small and large simulations vary across the models, due to differences between the degree of aggregation (cf. section 5.1) and how a given amount of self‐aggregation imprints on the mean state, there are robust qualitative responses to self‐aggregation across the RCEMIP ensemble. The RCE_large300 simulations, which are generally aggregated, have a larger precipitation rate than their RCE_small300 counterparts (Figure 13e). This is energetically consistent with greater net atmospheric radiative cooling and larger surface enthalpy fluxes (Table A3). The existence of self‐aggregation results in a reduction in high cloud cover, as indicated by changes in cloud fraction and total cloud water (Figures 13a and 13b). Figures 13a and 13b also indicate that, while less robust, most models have an increase in low‐level and mid‐level cloudiness with aggregation. We note, however, that the difference in horizontal grid spacing between the RCE_small and RCE_large CRM configurations may also contribute to the difference in low cloudiness (Blossey et al., 2009; Vial et al., 2019). The opposing changes in low, middle, and high clouds result in a difference in total cloud water path that may be positive or negative, depending on the model, but is on average near zero (Figure 13g; Table A3). These changes in clouds with aggregation are consistent with the conclusions from past studies of self‐aggregation, as presented by Wing (2019).

Comparing the aggregated state in RCE_large300 to the disaggregated state in RCE_small300, most models experience a substantial reduction in PW (an average reduction of 4.9 mm; Table A3; Figure 13h), in relative humidity at most individual levels in the free troposphere, with the largest reductions in the midtroposphere (Figure 13c), and in column relative humidity (not shown), consistent with prior studies of self‐aggregation (Wing, 2019, and references therein). All models except WRF‐CRM (which is not aggregated) also have an increase in atmospheric temperature by several degrees (Figure 13d), and an associated decrease in the tropospheric lapse rate (Figure 13i). Regarding the radiative budget, the net radiation at the top of atmosphere is reduced in the RCE_large300 simulations by ∼24 W m^−2^, indicating that less radiative energy is entering the top of atmosphere, reducing the radiative energy source to the climate system (Figure 13f; Table A3). This is largely explained by greater OLR in the RCE_large300 simulations due to, on average, a drier and more transparent atmosphere with fewer high clouds. The average difference in OLR between pairs of RCE_large300 and RCE_small300 simulations is ∼25 W m^−2^ (Table A3), while the average difference in clear‐sky OLR is ∼14 W m^−2^ (not shown), indicating that clear‐sky processes contribute a bit more than half of the difference between aggregated and unaggregated simulations. There is a minimal change in the top of atmosphere absorbed solar radiation due to opposing changes in low, middle, and high clouds (Table  A3). The cancellation in the changes in the shortwave fluxes and resultant longwave‐driven decrease in net top of atmosphere radiation are consistent with other RCE simulations (Wing & Cronin, 2016) and some observations (Bony et al., 2020), but in opposition to other observations of more/less aggregated (by any mechanism) convection (Tobin et al., 2012).

## 6 Sensitivity to SST

Having assessed the robustness of the RCE state across the spectrum of models (section 4) and the impact of self‐aggregation on mean climate (section 5), we now turn to the response of clouds, convective aggregation, and the radiative budget to warming. In all cases, we assess the response to warming using the change between simulations at 295 and 305 K (the end points of our SST range). Note that with three simulations, this is equivalent to the slope of the line of best fit (using linear regression) across the three simulations. We also examine changes between individual pairs of simulations (295–300 K and 300–305 K), to check for nonmonotonic behavior and variations in the rate of change in simulation properties with temperature.

### 6.1 Clouds

The anvil cloud peak was determined by identifying the maximum domain‐wide cloud fraction above a height of 8 km, using each model's own levels. A more restricted height range was used in several models based on subjective interpretation of their cloud fraction profiles to ensure the correct cloud peak was identified: CAM6‐GCM, 8–12.5 km; WRF‐GCM, greater than 10 km; SAM‐GCRM, 8–20 km; DALES, 8–15 km; and SAM‐CRM RCE_small_vert and RCE_small_les, 8–15 km. After the anvil cloud peak was identified, the height and temperature at that location were taken to represent the anvil cloud height and anvil cloud temperature. In the UKMO‐RA1‐T, UKMO‐RA1‐T‐hrad, and MPAS RCE_small simulations, there is a thick layer of 100% high cloud coverage. Therefore, in these simulations, the anvil cloud height and temperature were taken to be the average of the values between the bottom and top of the anvil cloud layer. In all cases, temporal averages neglecting the first 75 days of simulation are considered.

#### 6.1.1 Anvil Cloud Height

**Figure 14:**
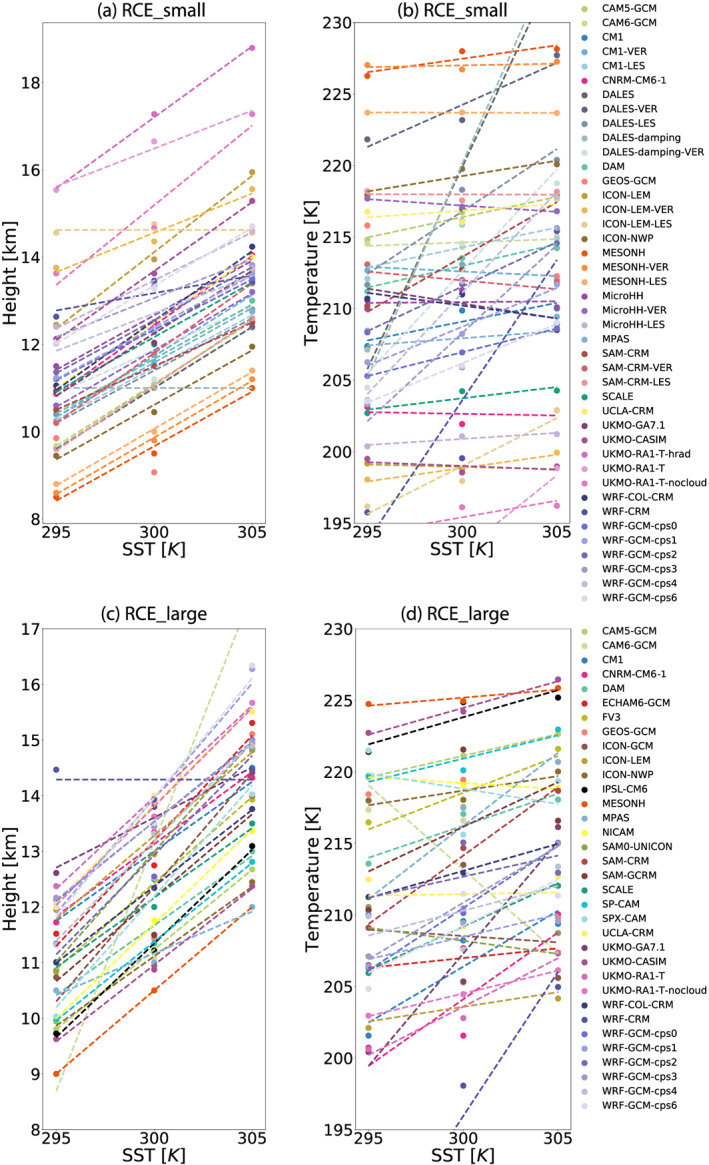
Horizontal‐ and time‐mean height (a, c) and temperature (b, d) at the location of the domain‐wide anvil cloud peak as a function of SST in the RCE_small simulations (a, b) and RCE_large simulations (c, d). The dashed lines are linear regression lines of best fit.

Across both the RCE_small and RCE_large sets of simulations at 295, 300, and 305 K, the anvil cloud peak shifts upward with warming (Figures 14a and 14c; Tables S4 and S7), as expected from previous work (Hartmann & Larson, 2002). The average height increase is 0.2 km K^−1^ in the RCE_small simulations, with 57% of models giving values within 0.2–0.3 km K^−1^ and 24% exhibiting increases in anvil height smaller than 0.2 km K^−1^ (Figure 14a; Table S4). In the RCE_large simulations, the average height increase is 0.3 km K^−1^, with 79% of models within 0.2–0.4 km K^−1^ and 9% below 0.2 km K^−1^ (Figure 14c; Table S7). The cloud heights themselves in an individual simulation have less spread in RCE_large than in RCE_small (Figures 14a and 14c; Tables S4 and S7). There are several outliers with notably smaller or larger height increases across the three simulations. In the RCE_smallsimulations, WRF_CRM has a rate of height increase that is an order of magnitude less, due to nonmonotonic behavior (the height of the anvil cloud increases from 295 to 300 K but decreases from 300 to 305 K), whereas DALES and DALES‐damping have no change in anvil height across the SSTs and ICON‐LEM‐LES has no change due to nonmonotonic behavior. In the RCE_large simulations, five of the GCMs have a much larger increase in anvil cloud height than the other models, whereas WRF_CRM has no overall trend from 295 to 305 K because of nonmonotonic behavior. Overall, 70% of the models have an increase in anvil cloud height that is larger from 300 to 305 K than it is from 295 to 300 K, indicating that the anvil cloud height increases with warming at an increasingly faster rate.

#### 6.1.2 Anvil Cloud Temperature

Prior work has shown that the location of anvil clouds is determined by the vertical gradient in radiative cooling, which is in turn controlled by water vapor concentrations and thus occurs at a fixed temperature (FAT; Hartmann & Larson, 2002) independent of surface temperature or is proportionally higher at higher surface temperature since the anvil rises into an environment with greater static stability (PHAT; Zelinka & Hartmann, 2010). While the FAT/PHAT hypothesis is generally found to hold (Harrop & Hartmann, 2012; Khairoutdinov & Emanuel, 2013; Kluft et al., 2019; Kuang & Hartmann, 2007), its validity has recently been questioned (Seeley, Jeevanjee, Langhans, & Romps, 2019; Seeley, Jeevanjee, & Romps, 2019). A detailed investigation of the validity of FAT/PHAT in the RCEMIP ensemble is deferred to future work, but we present a brief analysis here. Across the RCEMIP simulations, the anvil cloud temperature generally increases with warming, by an average of 4.4 K across the 10 K increase in SST, in line with Zelinka and Hartmann (2010) and Kluft et al. (2019) (Figures 14b and 14d; Tables S3 and S6). However, 25% of the RCE_small simulations actually have a decrease in anvil cloud temperature with warming; five of these 11 simulations were those with higher vertical (RCE_small_vert) and horizontal resolution (RCE_small_les; Figure 14b; Table S3). The coarse‐resolution counterparts of these five RCE_small_vert and RCE_small_les models do not have a decrease in anvil cloud temperature with warming, which is suspicious and requires further investigation. The result that some simulations have a decrease in anvil cloud temperature is surprising and may indicate that our method for diagnosing the anvil cloud peak may need to be modified in future work, though we note that the anvil cloud height changes in those models were comparable to the others. In the RCE_large simulations, less than 20% of the models have a decrease in anvil cloud temperature with warming (Figure 14d; Table S6). All of the models with a decrease are global models, some of which have anomalously large changes in anvil cloud height. It is possible that in these models, the diagnosed high cloud peak does not correspond to actual anvil cloud detrainment and thus would not be constrained by radiative processes (i.e., PHAT) in the same way. Of those models that have an overall increase in anvil temperature with warming, which is the majority of models, in about half the rate of increase of anvil temperature with SST is larger at higher SSTs. To the extent that there is nonmonotonic behavior in the change in anvil cloud temperature, in the RCE_small simulations it mostly occurs in CRMs, while in the RCE_large simulations it mostly occurs in global models.

In summary, in the majority of models, anvil cloud temperatures increase with warming (Figures 14b and 14d) in line with PHAT (Kluft et al., 2019; Zelinka & Hartmann, 2010). Of those that instead have a decrease in anvil cloud temperatures, most are LES or global models (Figure S27).

#### 6.1.3 Anvil Cloud Fraction

**Figure 15:**
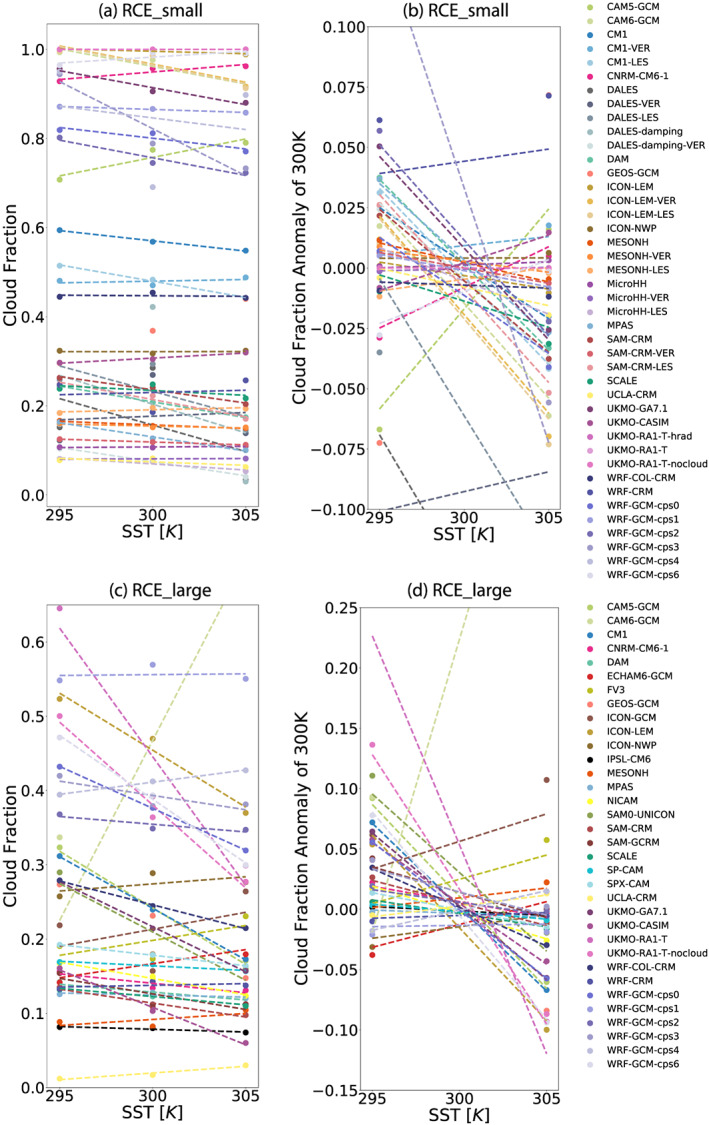
Domain‐wide anvil cloud fraction as a function of SST in the RCE_small simulations (a, b) and RCE_large simulations (c, d). The left panels (a, c) show the actual value of the anvil cloud fraction while the right panels (b, d) show the value of the anvil cloud fraction as an anomaly from its value in the simulation at 300 K. The dashed lines are linear regression lines of best fit.

In the RCE_small simulations, 27 models exhibit an overall decrease in anvil cloud fraction with warming consistent with the stability‐iris hypothesis of Bony et al. (2016), while 12 models instead have an overall increase in anvil cloud fraction with warming (Figures 15a and 15b; Table S2). Three models have domains filled with cloud at every SST, according to the threshold used here to define cloud, and therefore do not have a changing anvil cloud fraction (Figure 15a; Table S2). Nine of the models have a trend in anvil cloud fraction from 300–305 K that is 1 or 2 orders of magnitude below that of 295–300 K. A third of the models are nonmonotonic, consisting mostly of those whose rate of change of anvil cloud fraction is roughly equal in magnitude and opposite in sign from 295–300 K and 300–305 K. Of the 12 models that have an increase in cloud fraction with warming in the RCE_small simulations, seven have a rate of change less than 0.0015 K^−1^, which is substantially less than those models that have a decrease (they have a mean rate of change of −0.006 K^−1^).

In the RCE_large simulations (Figures 15c and 15d; Table S5), most models have a decrease in cloud fraction with warming, but there is an increase in 30% of the models, which includes the majority of those that have nonmonotonic changes. However, of those with nonmonotonic changes, only half have substantial nonmonotonic behavior, characterized by a large jump in cloud fraction from an otherwise negative trend (e.g., CAM6‐GCM whose upper atmosphere completely fills with clouds at 305 K). The other half have a decrease in cloud fraction from 295 to 300 K and only a slight increase (near zero change) from 300 to 305 K (e.g., CNRM‐CM6‐1).

The rate of change of anvil cloud fraction in the RCE_large simulations is either constant with SST or decreases as SST increases, which suggests a potential limit to how much anvil cloud fraction can decrease with warming SST (Bony et al., 2016). In RCE_small there is no trend in the rate of change of anvil cloud fraction with SST.

In summary, in the majority of models, anvil cloud fraction decreases with warming (Figure 15), with no obvious differences in the responses between models with parameterized and explicit convection (Figure S28).

#### 6.1.4 Low‐Level and Mid‐Level Clouds

While our focus is on anvil cloud changes, here we briefly discuss changes in low‐level and mid‐level clouds. As described in section 4.3, most models have an identifiable low‐level peak in cloudiness near the top of the boundary layer (∼0–3 km). Mid‐level clouds are defined subjectively as the part of the cloud fraction profile between the low‐level and anvil peaks (roughly ∼3–8 km). The low‐level clouds, if a notable peak exists, tend to not change with warming. In the RCE_small simulations, 63% of the models do not have an obvious change in low‐level cloudiness while 11% have no discernible low‐level cloud peak. In the RCE_large simulations, 47% of the models do not have an obvious change in low‐level cloudiness while 16% have no discernible low‐level cloud peak at any SST.

The mid‐level cloud fraction in the RCE_small simulations also seems to remain constant with warming whereas half of the RCE_large simulations exhibit a reduction in mid‐level cloud fraction with warming, and the other half have no change. Of those models whose mid‐level cloud fraction does decrease with warming, about half have a cloud fraction that changes more from 295–300 K than from 300–305 K.

#### 6.1.5 Summary

The response of cloud properties to warming varies across models, but some common behavior is found. Anvil cloud height and temperature, on average, increase with increasing SST at rates of ∼0.3 km K^−1^ and ∼0.44 K K^−1^, respectively. While there are some outliers, the rising and warming of anvil clouds are robust across types of models and domain configurations and occur regardless of the occurrence of self‐aggregation. Anvil cloud fraction decreases with warming across a majority of models, at a rate that decreases with warming, but approximately 30% of the models exhibit an increase in anvil cloud fraction with warming instead. There is no obvious difference in behavior between models with parameterized and explicit convection (Figures S26–S28). We note that these results should be interpreted cautiously, as some of the anvil clouds identified by the current definition may be thin and radiatively unimportant. Future work on the changes in cloud properties may consider an alternate metric for determining the spatial coverage of ice clouds.

### 6.2 Self‐Aggregation

**Figure 16:**
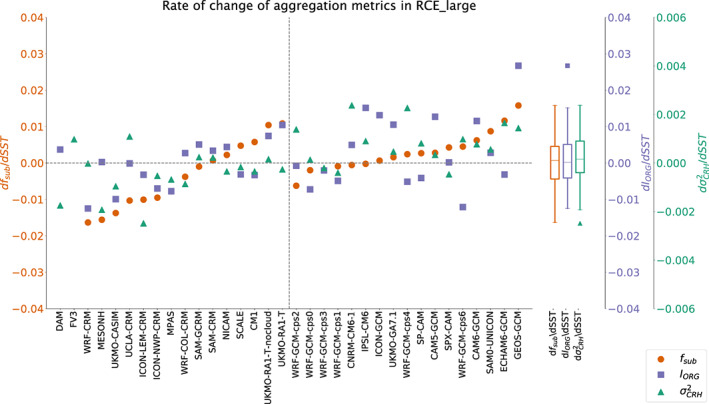
The rate of change of the aggregation metrics per degree K in the RCE_large simulations based on *I*_*org*_ (blue squares), subsidence fraction (red circles), and spatial variance of column relative humidity (green triangles) in all models, based on the difference between simulations at 295 and 305 K. The models are ordered such that the models with explicit convection are to the left of the dashed line and models with parameterized convection are to the right of the dashed line. Within each group of models, they are ordered according to their values of *df*_*sub*_/*dSST*. The two models for which subsidence fraction could not be computed (due to missing output) are listed first. Box plots indicate the spread of each metric's rate of change across models, with outlier indicated with symbols.

There is no consistent response of the degree of aggregation to warming; across the set of three RCE_large simulations from 295 to 305 K, about half of the models have a net increase in aggregation with warming while the other half have a net decrease in aggregation with warming (Figure 16). This is true regardless of whether *I*_*org*_, *f*_*sub*_, or $\sigma_{CRH}^{2}$ is used to quantify the degree of aggregation. Averaged across all models, the average values of *I*_*org*_, *f*_*sub*_, and $\sigma_{CRH}^{2}$ at 295, 300, and 305 K are not statistically distinguishable fromeach other.

Even within a given model, different metrics of aggregation may indicate different behavior regarding the response of aggregation to warming, as was also noted by Cronin and Wing (2017). For those models in which all three metrics are available (all but three), ∼53% of models have disagreement on the sign of the response of aggregation to warming between the metrics (Figure 16). It is roughly evenly split among which of three metrics disagrees. Of those models in which all three metrics agree on the sign of change, two thirds have an increase in aggregation with warming. As for the magnitude of the change in aggregation with warming, most models are within the same order of magnitude of change, while ∼30% of models have rates of changes of the metrics that are an order of magnitude larger. Across the entire ensemble of models, there is no obvious sensitivity of the rate of change of aggregation to temperature; roughly half of the models have a larger magnitude of a change in aggregation for 295 to 300 K, while the other half have a larger magnitude of change for 300 to 305 K (cf. Figures 16 and S21). Several individual models, however, exhibit nonmonotonic behavior; for example, WRF‐CRM is not aggregated in RCE_large300 (section 5), but it appears to be weakly aggregated in RCE_large295 and RCE_large305 with several dry patches, a somewhat wider distribution of PW (Figure S10; Movie S28), and opposing signs in the rate of change of aggregation metrics between 295–300 K and 300–305 K (Figure S21).

As noted in section 3, UKMO‐RA1‐T is the only RCE_small300 simulation that is aggregated. Compared to other models, UKMO‐RA1‐T, DALES, and DALES‐damping have broader PW distributions in RCE_small305, indicating higher SSTs may increase the likelihood of RCE_small simulations aggregating (Figure S10). The temporal variability of aggregation is also temperature dependent in some models; for example, the convective clusters move less in ECHAM at the simulations at higher SSTs (Movie S6). In many of the GCMs, the spatial scale of the aggregation qualitatively appears to increase with warming (see animations in the supporting information). Several of the CRMs indicate a decrease in the scale of aggregation with warming (i.e., FV3, ICON‐NWP, and UKMO‐RA1‐T‐nocloud), while others show no obvious change. A detailed quantification of the spatial scale of aggregation is deferred to future work.

### 6.3 Radiative Budget

**Figure 17:**
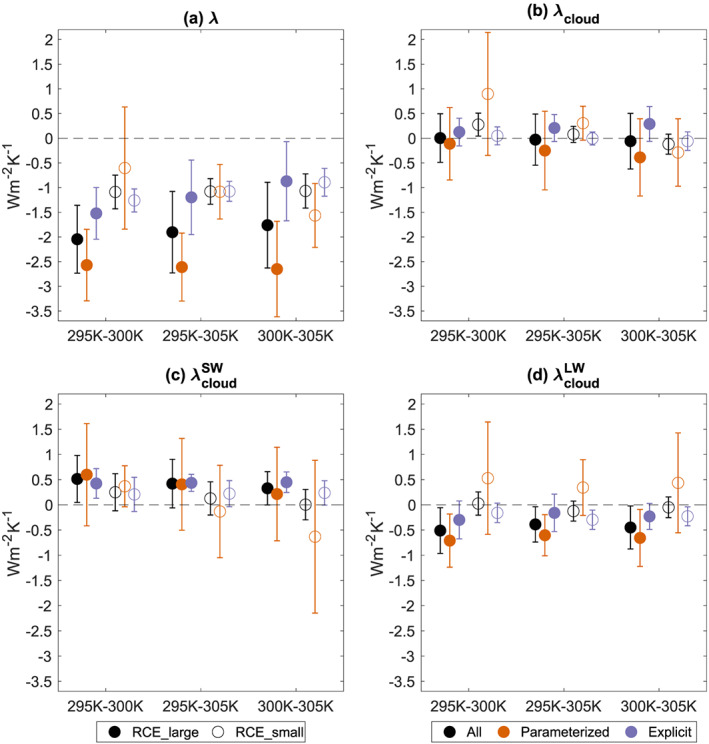
Net climate feedback parameter *λ* = *dR*_TOA_/*dT* (a), change in cloud radiative effect *λ*_cloud_ = *dCRE*/*dT* (b), change in shortwave cloud radiative effect $\lambda_{\text{cloud}}^{\text{SW}}=dCRE_{SW}/dT$ (c), and change in longwave cloud radiative effect $\lambda_{\text{cloud}}^{\text{LW}}=dCRE_{LW}/dT$ (d). RCE_large simulations are shown in filled symbols; RCE_small simulations are shown in open symbols. Averages over all models are shown in black, averages over models with parameterized convection are shown in red, and averages over models with explicit convection are shown in blue. The feedbacks are calculated over 295–300, 295–305, and 300–305 K, as indicated on the *x* axis. The error bars indicate the interquartile range.

The changes in clouds and convective aggregation described above have implications for the atmospheric radiative budget and climate sensitivity. We compute the Cess‐type net climate feedback parameter, *λ*=*dR*_TOA_/*dT*, which is the rate of change of net top‐of‐atmosphere energy gain *R*_TOA_ with increasing surface temperature (Cess & Potter, 1988) (Figure 17a; Tables S8 and S11). The average net climate feedback parameter in the RCE_small simulations is *λ*=−1.08 W m^−2^ K^−1^ (Table S8) and, in the RCE_large simulations, *λ*=−1.90 W m^−2^ K^−1^ (Table S11). This indicates that, averaged across all models, aggregated RCE_large simulations have lower climate sensitivity than the unaggregated RCE_small simulations (consistent with Cronin & Wing, 2017). However, there is a wide spread in *λ* (Tables S8 and S11), and the difference in average *λ* between RCE_small and RCE_large is primarily contributed by models with parameterized convection and is less apparent in those with explicit convection. Of those models that completed both RCE_large and RCE_small simulations, ∼70% have a more negative *λ* in RCE_large, which breaks down to ∼90% of models with parameterized convection and ∼50% of models with explicit convection.

Averaged across all models with explicit convection, *λ* becomes less negative with increasing SST, indicating an increase in climate sensitivity with warming (Figure 17a). The models with parameterized convection do not have this dependency. The average net climate feedback parameter in the RCE_small simulations is the same in models with explicit and parameterized convection. However, in the RCE_large simulations, the average net climate feedback parameter is more negative in the models with parameterized convection (*λ*=−2.61 W m^−2^ K^−1^) than in the models with explicit convection (*λ* = −1.20 W m^−2^ K^−1^), indicating that the models with parameterized convection have lower climate sensitivity (Table S11). Across all models, the longwave component of *λ* is on average negative while the shortwave component is on average positive (Tables S9 and S12).

We estimate the cloud radiative feedback (*λ*_cloud_ = *dCRE*/*dT*) as the change in cloud radiative effect (CRE) with increasing surface temperature, where CRE is computed by subtracting the clear‐sky from the all‐sky top‐of‐atmosphere radiative fluxes (*R*_TOA_−*R*_TOA,clear−sky_). We find that the cloud feedback is on average *λ*_cloud_ = 0.08 W m^−2^ K^−1^ in the RCE_smallsimulations and *λ*_cloud_ = −0.03 W m^−2^ K^−1^ in the RCE_large simulations (Figure 17b; Tables S8 and S11), but this small difference is within the error bars and thus does not explain the difference in the net climate feedback between RCE_small and RCE_large simulations. The cloud feedback in the RCE_small simulations is positive from 295 to 300 K and negative from 300 to 305 K, resulting in a small residual near zero when considering the net feedback across all simulations. Considering only the models with explicit convection, *λ*_cloud_ is near zero across all temperature ranges. In the RCE_large simulations, the cloud feedback is negative in the models with parameterized convection (*λ*_cloud_ = −0.25 W m^−2^ K^−1^) but positive in the models with explicit convection (*λ*_cloud_ = 0.21 W m^−2^ K^−1^). This results from differences in the magnitude of the longwave cloud feedback (Figure 17d; Table S13). However, as the difference in the average *λ*_cloud_ is within the error bars, the difference in cloud feedback between models with parameterized and explicit convection is not sufficient to fully explain the difference the net climate feedback noted above. We also note that the uncertainty in the *λ*_cloud_ estimate is substantially smaller, in both RCE_small and RCE_large, in models with explicit convection, as indicated by the smaller interquartile range compared to those with parameterized convection (Figure 17b; Tables S8 and S11).

When splitting the cloud feedback into its longwave and shortwave components, we find that the shortwave cloud feedback is in general positive ( $\lambda_{\text{cloud}}^{\text{SW}}=0.42$ W m^−2^ K^−1^ when averaged over all models across all RCE_large simulations; Figure 17c; Table S13). In the RCE_small simulations, the shortwave cloud feedback is positive in models with explicit convection ( $\lambda_{\text{cloud}}^{\text{SW}}=0.44$ W m^−2^ K^−1^) but on average negative ( $\lambda_{\text{cloud}}^{\text{SW}}=-0.13$ W m^−2^ K^−1^) in those with parameterized convection (Figure 17c; Table S10). $\lambda_{\text{cloud}}^{\text{SW}}$ is even more negative ( $\lambda_{\text{cloud}}^{\text{SW}}=-1.26$ W m^−2^ K^−1^) when considering only single‐column versions of GCMs (excluding WRF‐GCM). For comparison, in comprehensive global climate model simulations, decreasing tropical low cloud amount leads to a positive shortwave feedback (Ceppi et al., 2017). Deviations from this in some of the RCEMIP simulations may reflect a lack in RCE of low clouds that in nature occur in regions of strong subsidence and high lower tropospheric stability and/or cloud masking effects. Notably, there is a large temperature dependence of the shortwave cloud feedback in the RCE_small simulations in models with parameterized convection that is diminished in the RCE_large simulations and absent in models with explicit convection. The longwave cloud feedback is in general negative, except in the RCE_small simulations in models with parameterized convection and is of similar magnitude to the shortwave cloud feedback (Figure 17d). The negative longwave cloud feedback is a combination of anvil cloud altitude and amount feedbacks but is consistent with the decrease in anvil cloud fraction with warming across most models. Of those models that completed both RCE_large and RCE_small simulations, ∼60–70% have a more positive $\lambda_{\text{cloud}}^{\text{SW}}$ in RCE_large across both model types. Approximately 55% have a more negative $\lambda_{\text{cloud}}^{\text{LW}}$ in RCE_large, but this is dominated by the response in models with parameterized convection, in which ∼80% have a more negative $\lambda_{\text{cloud}}^{\text{LW}}$ in RCE_large compared to only ∼30% of models with explicit convection. These combined results indicate that aggregation may play a role in modulating cloud feedbacks.

We caution that these estimates of cloud feedbacks are crude and neglect cloud masking effects, which generally are on the order of ∼0.3–0.4 Wm^−2^K^−1^ and can be enough to change the sign of the cloud feedback (Soden et al., 2004). Indeed, Cronin and Wing (2017) found that corrections to changes in cloud radiative forcing with warming based on radiative kernel calculations (Soden et al., 2008) were important for obtaining an overall positive cloud feedback in RCE simulations. We intend to perform these more accurate calculations of cloud feedbacks using the approximate kernel method developed for RCE (Cronin & Wing, 2017) in future work.

In summary, there is a wide range of net climate feedbacks in the RCEMIP simulations, but in the RCE_large simulations, models with parameterized convection robustly have more negative climate feedbacks (corresponding to lower climate sensitivity) than models with explicit convection. There is also evidence that, particularly in the models with parameterized convection, RCE_large simulations have more negative climate feedbacks than the RCE_small simulations. In both cases, the differences cannot be completely explained by our rough estimate of cloud feedbacks and are thus more likely to be due to clear‐sky processes or cloud masking effects. The intermodel spread in the estimate of cloud feedbacks is notably smaller in models with explicit convection.

## 7 Conclusions

This paper has presented the first results of RCEMIP, the first model intercomparison to include CRMs, GCRMs, LES, GCMs, and SCMs, with a focus on providing a broad overview with respect to each of the three themes of RCEMIP.

The first theme of RCEMIP is the robustness of the radiative‐convective equilibrium (RCE) state across the spectrum of models. RCE is a simple concept, in which models achieve a balance between convective heating and radiative cooling of the atmosphere. However, because moist convection is transient and involves complex interactions with radiation and circulation, this balance is only achieved in a statistical sense and is sensitive to how convection is simulated. Indeed, when confronted with the same boundary conditions and forcing, the models in the RCEMIP ensemble exhibit a diversity of responses. As described in section 4, there are substantial differences in the representation of temperature, humidity, and cloudiness. Temperature profiles are robustly several degrees cooler than a moist adiabatic profile, but the range of temperatures at a given height in the troposphere across models can be up to 10 K, the tropopause height and tropopause temperature vary, and 2 m air temperature varies by more than 1 K. Relative humidity varies by more than a factor of 2 in the free troposphere and by one third near the surface. In both simulations with and without aggregated convection, there is a wide spread in the amount of upper level cloudiness and the height at which the anvil cloud peak occurs (in addition to disagreements for low‐level and mid‐level clouds). The large spread across models is also apparent in variables related to the top‐of‐atmosphere energy budget and hydrological cycle. These disagreements occur both in models with explicit convection and those with parameterized convection.

RCEMIP also aims to determine the response of clouds to warming and the climate sensitivity in RCE. Across simulations at 295, 300, and 305 K, high clouds shift upward and warm in response to increasing SST. This response is found in the majority of models across different types of models and domain configurations. In ∼70% of models, high clouds reduce in area fraction with warming at a rate that decreases with warming. There is no clear response of low‐level and mid‐level clouds to warming, though low cloud fraction is low to begin with (RCE is unfavorable for certain tropical low clouds, such as stratocumulus), which may contribute to a smaller change in cloud fraction and weaker low cloud feedback (Brient & Bony, 2012). The average net climate feedback parameter is generally more negative in the aggregated RCE_large simulations compared to the unaggregated RCE_small simulations, mostly due to clear‐sky effects. However, this is only robustly true in models with parameterized convection, in which RCE_small is performed with SCMs or a four‐column configuration of WRF‐GCM, which may reflect issues other than the absence of aggregation. In the models with explicit convection, the net climate feedback parameter becomes less negative with warming and both the shortwave and net cloud radiative effect generally increase with warming. This response differs in models with parameterized convection. Models with explicit convection in RCE_large have a less negative climate feedback parameter than those with parameterized convection, corresponding to higher climate sensitivity, which is mostly attributed to clear‐sky effects. The intermodel spread in the estimate of cloud feedbacks is also notably smaller in models with explicit convection.

The final theme of RCEMIP is the dependence of convective self‐aggregation on temperature. With a few exceptions, self‐aggregation is absent in RCE_small simulations and present in RCE_large simulations. However, the spatial patterns of convection are diverse and quantitative metrics of the degree of aggregation vary widely. Models agree that self‐aggregated simulations produce atmospheres that are warmer, drier, have reduced high cloudiness, and cool more to space than their unaggregated counterparts. However, there is no consensus on whether self‐aggregation increases or decreases with warming; about half of the models have a net increase in aggregation with warming while the other half have a net decrease in aggregation with warming.

In summary, despite some robust behaviors, there is substantial disagreement across the RCEMIP ensemble in representations of cloudiness, self‐aggregation, and climate sensitivity. Some readers may find this discouraging or surprising (perhaps hoping that models with explicit convection might have agreed better), while some readers may have anticipated that the many degrees of freedom in how models may achieve RCE would result in divergent behavior. Indeed, because RCE is relatively unconstrained, with convection left free to evolve as long as energy balance is still met, it is a tough test for models. We argue that this is a benefit of RCE, rather than a weakness. The divergent behavior in RCEMIP reveals the true sensitivities to representations of convection, microphysics, turbulence, and dynamical cores, sensitivities that might be masked in other comparisons by constraints imposed by large‐scale circulations. Furthermore, the RCEMIP results show that the wide range of equilibrated states is not due to differences in the basic configuration such as SST, CRM grid spacing, insolation, or initialization, as there is a large spread despite constraining these factors to be the same. Instead, the different responses must be due to differences in model physics and/or numerics.

The results presented here introduce many further questions, some of which will be addressed in separate papers planned on (i) climate sensitivity and self‐aggregation, (ii) explanation of cloud changes with warming, and (iii) behavior of specific families of models. Other questions, such as further details of self‐aggregation, including its spatial scale, should also be addressed in future work. One of the biggest open questions is *why* these models disagree so much, even in such a simplified setting. While much of the simulation setup was designed to be consistent across models, the models differ enormously in their choice of subgrid‐scale parameterizations, grids, and numerics. This was a conscious choice in the design of RCEMIP, as Wing et al. (2018) sought to make it as easy as possible for models to participate with something close to their “out of the box” configuration and sought to know how different the resulting RCE states actually would be. Is there one particular aspect of the model configuration that has a dominant impact on the spread? Answering this question will likely require targeted parameter perturbation experiments with particular models that represent the range of the RCEMIP ensemble, which could motivate a second phase of RCEMIP in which the model configurations are constrained to be more similar, such as by imposing simplified, or at least consistent, physics packages.

The RCEMIP ensemble presents a large collection of simulations in an idealized framework spanning a wide range of model types. The results presented here are just the beginning of what we can learn from this ensemble, and we hope that they motivate further analyses of this now public data set.

## Supporting information



Supporting Information S1

## Data Availability

We thank the German Climate Computing Center (DKRZ) for hosting the standardized RCEMIP data, which is publicly available online (at http://hdl.handle.net/21.14101/d4beee8e-6996-453e-bbd1-ff53b6874c0e).

## Appendix A Domain‐Average Statistics

### A.1

This appendix presents tables of domain‐average statistics for the small and large domain simulations at 300 K (Tables A1 and A2) and, for models who performed both simulations, the difference between the two (Table A3). Domain and time averages (neglecting the first 75 days of simulation, except for RCE_small_les300 for which an average over Days 25–50 is used) are provided for each model for the following variables: the atmospheric energy imbalance (F_Net_, the magnitude of difference between the energy imbalance at the surface and top of atmosphere), the net radiation at the top of atmosphere (R_TOA_ = ASR − OLR, where positive values indicate net radiation into the atmosphere), the implied ocean heat uptake at the surface (Q_OCN_ = R_SFC_ − LHF − SHF, where positive values indicate a flux into the ocean), the column net radiative flux convergence (R_Net_ = R_TOA_ − R_SFC_, where negative values indicate net atmospheric radiative cooling), the OLR, the top of atmosphere absorbed solar radiation (ASR), the surface latent heat flux (LHF), the surface sensible heat flux (SHF), PW, surface precipitation rate (Precip.), liquid water path (LWP), ice water path (IWP), and tropospheric lapse rate. The last rows of the tables indicate the multimodel mean and measures of the intermodel spread (standard deviation and interquartile range); an entry is listed as NaN if that variable was not provided.

**Table A1:** RCE_small300

	*F*_*NET*_	*R*_*TOA*_	*Q*_*OCN*_	*R*_*Net*_	OLR	ASR	LHF	SHF	PW	Precip.	LWP	IWP	Lapse rate
Model	W m^−2^	W m^−2^	W m^−2^	W m^−2^	W m^−2^	W m^−2^	W m^−2^	W m^−2^	mm	mm day^−1^	mm	mm	K km^−1^
CAM5‐GCM	0.03	82.99	82.96	−72.38	229.04	312.03	63.50	8.91	35.5	2.2	0.028	0.012	−7.50
CAM6‐GCM	0.10	97.51	97.42	−65.03	233.35	330.87	54.15	10.98	34.4	1.9	0.017	0.026	−7.36
CM1	5.51	105.91	111.42	−88.50	243.03	348.94	75.07	7.92	42.6	2.8	0.051	0.094	−7.54
CM1‐VER	8.68	103.97	112.65	−90.51	245.37	349.34	74.58	7.24	41.7	2.7	0.045	0.095	−7.50
CM1‐LES	7.53	103.21	110.74	−91.42	245.02	348.23	76.78	7.11	43.1	2.8	0.052	0.093	−7.61
CNRM‐CM6‐1	1.83	58.88	57.05	−62.06	204.61	263.48	53.78	10.11	39.0	1.9	0.048	0.025	−7.80
DALES	5.99	101.73	107.71	−92.72	258.99	360.72	76.14	10.59	34.1	4.8	0.007	0.004	−7.44
DALES‐VER	11.76	98.96	110.72	−102.06	256.39	355.34	83.98	6.33	46.2	5.5	0.013	0.013	−7.03
DALES‐LES	7.23	103.05	95.82	−96.38	255.81	358.87	93.28	10.33	42.0	5.5	0.014	0.012	−7.32
DALES‐damping	5.28	101.91	107.19	−90.89	259.71	361.62	74.76	10.85	31.9	4.6	0.006	0.003	−7.54
DALES‐damping‐VER	5.24	105.04	110.27	−88.81	258.20	363.24	73.86	9.71	33.2	4.7	0.007	0.004	−7.48
DAM	5.47	101.34	95.88	−83.76	250.22	351.56	77.64	11.58	40.4	2.6	0.029	0.012	−7.54
GEOS‐GCM	1.78	88.10	89.88	−61.59	215.29	303.40	55.66	4.16	36.2	1.9	0.053	0.033	−7.30
ICON‐LEM	6.66	111.18	104.52	−65.29	226.00	337.18	62.29	9.66	39.3	2.1	0.034	0.021	−7.22
ICON‐LEM‐VER	6.57	110.15	103.58	−70.20	230.31	340.46	67.63	9.14	38.7	2.3	0.006	0.021	−7.16
ICON‐LEM‐LES	8.93	113.17	104.24	−64.01	220.08	333.25	64.43	8.51	44.0	2.2	0.018	0.026	−7.22
ICON‐NWP	3.89	108.56	104.66	−52.01	225.08	333.63	47.35	8.56	29.2	1.6	0.022	0.025	−7.51
MESONH	0.59	106.03	106.62	−72.56	226.59	332.61	63.01	8.97	38.5	1.7	−0.022	0.036	−8.06
MESONH‐VER	2.10	105.92	108.02	−73.25	226.78	332.70	62.13	9.02	39.4	1.9	−0.015	0.029	−7.68
MESONH‐LES	1.39	105.16	103.76	−69.14	224.53	329.69	62.16	8.37	38.3	1.6	−0.027	0.038	−7.95
MicroHH	4.21	110.89	115.10	−85.66	239.79	350.68	71.12	10.33	40.2	3.4	0.002	0.008	−7.02
MicroHH‐VER	1.61	112.91	111.30	−84.72	234.78	347.69	75.99	10.34	44.3	4.5	0.015	0.010	−6.97
MicroHH‐LES	7.62	114.49	122.12	−82.26	231.85	346.34	66.27	8.37	43.5	2.5	0.015	0.012	−7.17
MPAS	16.31	86.27	69.95	−82.44	267.67	353.93	86.07	12.68	26.9	3.0	0.035	0.095	−7.91
SAM‐CRM	3.62	100.97	97.35	−85.42	238.05	339.02	79.12	9.92	41.6	2.7	0.038	0.044	−7.56
SAM‐CRM‐VER	2.75	103.73	100.98	−94.05	249.69	353.42	86.80	10.00	41.3	3.0	0.028	0.036	−7.43
SAM‐CRM‐LES	12.66	109.69	97.03	−82.23	237.90	347.59	85.00	9.90	41.1	2.9	0.019	0.039	−7.53
SCALE	4.42	104.74	100.32	−87.94	252.57	357.31	79.60	12.75	34.5	2.8	0.035	0.057	−7.83
UCLA‐CRM	7.72	88.79	96.51	−121.81	259.55	348.34	103.29	10.80	38.3	3.5	0.024	0.066	−6.70
UKMO‐GA7.1	0.34	115.89	115.55	−75.98	230.91	346.80	70.74	5.58	NaN	2.4	0.021	0.011	−7.55
UKMO‐CASIM	0.67	92.30	92.97	−100.05	263.39	355.69	92.18	7.20	37.9	3.2	0.006	0.074	−7.40
UKMO‐RA1‐T‐hrad	2.85	93.68	96.54	−93.30	258.02	351.70	85.64	4.81	34.3	3.0	0.006	0.043	−7.48
UKMO‐RA1‐T	3.05	87.30	90.35	−96.84	263.22	350.52	89.25	4.53	31.6	3.1	0.007	0.056	−7.38
UKMO‐RA1‐T‐nocloud	1.49	95.90	97.40	−91.41	255.06	350.96	85.16	4.75	35.8	3.0	0.005	0.060	−7.50
WRF‐COL‐CRM	3.23	109.65	106.42	−89.28	237.25	346.90	84.34	8.16	48.0	2.9	0.018	0.008	−7.13
WRF‐CRM	6.91	57.69	50.78	−108.66	254.53	312.23	80.09	35.49	44.6	2.7	0.069	0.081	−6.97
WRF‐GCM‐cps0	1.62	69.38	67.76	−83.34	223.21	292.59	78.17	6.79	43.5	2.8	0.095	0.015	−6.54
WRF‐GCM‐cps1	4.31	113.48	109.17	−97.21	253.12	366.60	91.07	10.45	37.7	3.2	0.000	0.004	−6.34
WRF‐GCM‐cps2	3.15	119.66	116.50	−82.30	228.95	348.61	78.20	7.25	46.7	2.6	0.000	0.015	−6.08
WRF‐GCM‐cps3	2.14	35.68	37.82	−97.96	232.79	268.46	84.63	11.19	37.6	3.0	0.121	0.011	−6.35
WRF‐GCM‐cps4	4.01	106.25	102.24	−94.55	262.13	368.38	93.02	5.54	27.5	3.2	0.000	0.004	−6.28
WRF‐GCM‐cps6	4.77	79.71	74.93	−85.77	223.23	302.93	82.38	8.17	43.2	2.8	0.048	0.017	−6.21
STD	3.53	17.18	18.13	13.92	15.67	23.98	12.33	4.66	5.1	1.0	0.028	0.028	0.48
IQR	4.74	16.73	13.05	19.22	26.65	20.16	18.30	3.09	7.1	0.8	0.029	0.032	0.48
Mean	4.67	98.14	97.48	−84.66	241.24	339.38	75.96	9.36	38.7	2.9	0.024	0.033	−7.26

**Table A2:** RCE_large300

	**F**_*NET*_	*R*_*TOA*_	*Q*_*OCN*_	*R*_*Net*_	OLR	ASR	LHF	SHF	PW	Precip.	LWP	IWP	Lapse rate
Model	W m^−2^	W m^−2^	W m^−2^	W m^−2^	W m^−2^	W m^−2^	W m^−2^	W m^−2^	mm	mm day^−1^	mm	mm	K km^−1^
CAM5‐GCM	0.09	56.40	56.32	−107.94	258.01	314.41	97.10	10.93	36.7	3.4	0.038	0.018	−7.12
CAM6‐GCM	0.05	66.23	66.28	−96.96	260.40	326.62	84.31	12.59	31.6	2.9	0.033	0.020	−7.43
CM1	7.58	86.09	93.67	−107.77	266.40	352.49	91.22	8.97	35.4	3.4	0.046	0.091	−7.40
CNRM‐CM6‐1	0.46	44.87	44.41	−115.80	270.49	315.36	105.58	10.68	30.5	3.6	0.014	0.031	−7.56
DAM	3.87	81.41	77.54	−104.80	268.33	349.74	97.30	11.37	36.3	3.3	0.032	0.010	−7.35
ECHAM6‐GCM	0.09	76.28	76.19	−107.83	273.64	349.91	90.33	17.60	32.0	3.1	0.017	0.009	−7.16
FV3	2.57	31.27	33.84	−121.70	274.82	306.09	109.05	10.09	29.4	3.7	0.060	0.020	NaN
GEOS‐GCM	2.02	65.35	67.37	−87.41	244.22	309.57	75.42	9.97	37.7	2.6	0.076	0.034	−6.43
ICON‐GCM	0.32	48.83	48.51	−105.28	271.22	320.04	90.76	14.84	26.6	3.1	0.034	0.019	−7.50
ICON‐LEM	6.80	90.20	83.40	−82.69	249.03	339.23	78.61	10.88	31.3	2.7	0.029	0.023	−7.13
ICON‐NWP	9.73	94.28	84.55	−63.99	235.05	329.33	63.62	10.11	28.2	2.2	0.026	0.034	−7.36
IPSL‐CM6	0.73	41.60	40.87	−136.58	284.34	325.93	126.97	10.34	33.2	4.3	−0.014	0.113	NaN
MESONH	1.23	68.13	69.35	−109.03	272.75	340.88	98.26	9.54	25.8	3.2	−0.004	0.022	−7.69
MPAS	6.80	73.50	66.70	−101.02	273.92	347.43	97.51	10.31	28.8	3.4	0.047	0.078	−7.50
NICAM	26.42	77.50	51.08	−110.28	265.93	343.43	129.15	7.55	31.0	3.6	0.060	0.050	−7.03
SAM0‐UNICON	0.00	48.14	48.14	−123.72	284.61	332.75	112.89	10.83	27.9	3.9	0.009	0.005	−6.87
SAM‐CRM	3.87	72.47	68.60	−118.05	274.66	347.12	113.15	8.77	31.2	3.9	0.048	0.025	−7.20
SAM‐GCRM	4.10	67.83	63.73	−121.32	276.43	344.26	116.68	8.74	31.9	4.0	0.082	0.026	−5.47
SCALE	3.18	87.40	84.22	−110.36	268.36	355.76	101.56	11.97	32.8	3.6	0.041	0.046	−7.47
SP‐CAM	2.10	73.62	71.53	−99.28	256.41	330.04	92.31	9.07	35.6	3.2	0.066	0.036	−7.23
SPX‐CAM	2.35	68.54	66.18	−105.06	259.07	327.60	98.30	9.11	36.1	3.4	0.072	0.034	−6.88
UCLA‐CRM	5.29	22.61	27.90	−157.66	294.71	317.33	135.60	16.77	26.4	4.8	0.039	0.052	−6.36
UKMO‐GA7.1	0.16	58.67	58.83	−125.92	273.84	332.51	120.22	5.53	34.5	4.2	0.047	0.024	−6.88
UKMO‐CASIM	3.90	74.28	70.38	−102.53	262.20	336.48	95.69	10.75	39.5	3.2	0.026	0.160	−6.90
UKMO‐RA1‐T	0.65	72.78	73.42	−110.86	281.21	353.99	102.35	7.87	28.6	3.6	0.009	0.089	−6.89
UKMO‐RA1‐T‐nocloud	1.54	68.71	67.17	−107.12	277.89	346.60	100.33	8.33	29.2	3.5	0.016	0.130	−6.84
WRF‐COL‐CRM	2.15	92.74	90.58	−103.21	251.77	344.51	97.10	8.27	41.7	3.4	0.019	0.013	−6.97
WRF‐CRM	21.73	56.07	34.34	−106.54	255.46	311.53	90.37	37.90	41.2	3.1	0.065	0.097	−6.91
WRF‐GCM‐cps0	4.46	26.57	22.11	−126.33	267.46	294.03	114.44	16.36	31.2	4.0	0.088	0.008	−5.63
WRF‐GCM‐cps1	6.70	88.27	81.58	−115.06	264.79	353.06	112.85	8.91	35.9	4.0	0.006	0.009	−5.88
WRF‐GCM‐cps2	3.72	96.24	92.51	−105.63	259.89	356.13	101.37	7.98	34.0	3.4	0.001	0.011	−5.89
WRF‐GCM‐cps3	0.91	12.00	12.90	−130.05	268.21	280.21	111.62	17.52	31.6	3.8	0.107	0.007	−5.64
WRF‐GCM‐cps4	1.81	99.31	97.50	−102.37	260.97	360.28	98.16	6.02	33.7	3.4	0.001	0.008	−6.06
WRF‐GCM‐cps6	2.85	71.86	69.01	−115.63	263.49	335.36	115.58	2.91	37.1	4.0	0.034	0.014	−5.82
STD	5.66	21.84	21.17	16.08	12.02	19.00	15.29	5.74	4.1	0.5	0.028	0.039	0.65
IQR	3.59	24.28	28.05	13.88	13.88	25.84	19.73	2.51	6.2	0.7	0.041	0.036	0.93
Mean	4.12	66.47	63.55	−110.17	266.76	333.24	101.93	11.16	32.8	3.5	0.037	0.040	−6.83

**Table A3:** RCE_large300‐RCE_small300

	Δ*R*_*TOA*_	Δ*R*_*Net*_	ΔOLR	ΔASR	ΔLHF	ΔSHF	ΔPW	ΔPrecip.	ΔLWP	ΔIWP	ΔLapse Rate
Model	W m^−2^	W m^−2^	W m^−2^	W m^−2^	W m^−2^	W m^−2^	mm	mm day^−1^	mm	mm	K km^−1^
CAM5‐GCM	−26.59	−35.55	28.96	2.38	33.60	2.02	1.2	1.2	0.010	0.006	0.38
CAM6‐GCM	−31.28	−31.93	27.04	−4.24	30.17	1.62	−2.8	1.0	0.016	−0.006	−0.06
CM1	−19.82	−19.26	23.37	3.55	16.14	1.06	−7.2	0.6	−0.005	−0.003	0.14
CNRM‐CM6‐1	−14.01	−53.74	65.89	51.87	51.80	0.57	−8.5	1.8	−0.034	0.005	0.24
DAM	−19.93	−21.04	18.11	−1.82	19.66	−0.21	−4.1	0.6	0.003	−0.002	0.19
GEOS‐GCM	−22.75	−25.82	28.93	6.18	19.76	5.82	1.5	0.7	0.023	0.000	0.86
ICON‐LEM	−20.98	−17.40	23.03	2.05	16.32	1.22	−8.1	0.5	−0.004	0.002	0.09
ICON‐NWP	−14.28	−11.99	9.98	−4.30	16.27	1.55	−1.0	0.5	0.004	0.009	0.15
MESONH	−37.90	−36.46	46.17	8.26	35.26	0.57	−12.7	1.5	0.018	−0.013	0.37
MPAS	−12.76	−18.58	6.26	−6.51	11.44	−2.37	1.9	0.5	0.012	−0.018	0.41
SAM‐CRM	−28.50	−32.63	36.60	8.10	34.02	−1.14	−10.4	1.1	0.010	−0.020	0.36
SCALE	−17.34	−22.42	15.79	−1.55	21.96	−0.78	−1.8	0.8	0.006	−0.011	0.35
UCLA‐CRM	−66.18	−35.85	35.17	−31.01	32.31	5.97	−11.9	1.2	0.015	−0.015	0.34
UKMO‐GA7.1	−57.22	−49.93	42.93	−14.29	49.48	−0.05	NaN	1.7	0.026	0.014	0.67
UKMO‐CASIM	−18.02	−2.48	−1.19	−19.21	3.51	3.55	1.7	0.0	0.020	0.086	0.51
UKMO‐RA1‐T(L)‐hrad(S)	−20.91	−17.57	23.20	2.29	16.71	3.06	−5.7	0.6	0.003	0.046	0.59
UKMO‐RA1‐T‐nocloud	−27.19	−15.72	22.83	−4.36	15.17	3.58	−6.6	0.6	0.011	0.070	0.66
WRF‐COL‐CRM	−16.91	−13.93	14.52	−2.39	12.76	0.10	−6.3	0.5	0.000	0.005	0.16
WRF‐CRM	−1.62	2.12	0.92	−0.70	10.29	2.41	−3.4	0.4	−0.004	0.016	0.07
WRF‐GCM‐cps0	−42.81	−43.00	44.25	1.43	36.27	9.57	−12.2	1.2	−0.007	−0.007	0.91
WRF‐GCM‐cps1	−25.21	−17.85	11.66	−13.54	21.78	−1.54	−1.8	0.7	0.006	0.005	0.46
WRF‐GCM‐cps2	−23.42	−23.33	30.94	7.52	23.17	0.73	−12.7	0.8	0.001	−0.003	0.19
WRF‐GCM‐cps3	−23.68	−32.09	35.43	11.75	26.99	6.34	−6.0	0.9	−0.014	−0.004	0.72
WRF‐GCM‐cps4	−6.95	−7.82	−1.16	−8.10	5.14	0.48	6.2	0.2	0.001	0.004	0.23
WRF‐GCM‐cps6	−7.85	−29.86	40.27	32.42	33.20	−5.26	−6.1	1.2	−0.014	−0.003	0.40
STD	14.55	13.67	16.36	15.82	12.41	3.11	5.2	0.4	0.013	0.025	0.25
IQR	10.28	15.23	20.91	10.54	17.06	3.11	6.6	0.6	0.016	0.012	0.32
Mean	−24.16	−24.57	25.20	1.03	23.73	1.55	−4.9	0.8	0.004	0.006	0.38

The average magnitude of atmospheric energy imbalance across RCE_small300 is F_Net_ = 4.67 W m^−2^ (Table A1), but the models with explicit convection are more out of balance (F_Net_ = 5.55 W m^−2^) than those with parameterized convection (F_Net_ = 2.19 W m^−2^). This relationship is also true in RCE_large300 (Table  A2). Note that F_Net_ should be zero if energy is conserved and the simulation is equilibrated. The actual value of F_Net_ is positive in some models and negative in others; here we consider the magnitude only. The implied ocean heat update is on average Q_OCN_ = 98.14 W m^−2^ in RCE_small300 (Table A1) and is larger in models with explicit convection (Q_OCN_ = 101.75 W m^−2^) than those with parameterized convection (Q_OCN_ = 87.96 W m^−2^). This relationship is also true in RCE_large300 (Table A2), but in those simulations, the implied ocean heat uptake is smaller (Q_OCN_ = 66.47 W m^−2^).

In RCE_small300, R_Net_ and OLR are on average larger in magnitude in the models with explicit convection, indicating the atmosphere is cooling more strongly compared to models with parameterized convection (R_Net_ = −86.37 W m^−2^ vs. −79.84 W m^−2^, OLR = 245.01 W m^−2^ vs. 230.60 W m^−2^). This is consistent with the larger values of R_TOA_, ASR, surface latent and sensible heat fluxes, and precipitation in the models with explicit convection (R_TOA_ = 101.75 W m^−2^ vs. 87.96 W m^−2^, ASR = 346.76 W m^−2^ vs. 318.56 W m^−2^, LHF = 76.94 W m^−2^ vs. 73.21 W m^−2^, SHF = 9.80 W m^−2^ vs. 8.10 W m^−2^, Precip = 3.1 mm day^−1^ vs. 2.5 mm day^−1^). The models with explicit convection also have a higher fraction of condensed water in ice form (IWP/(LWP + IWP) = 0.68 vs. 0.59). In RCE_large300 (Table A2), models with explicit and parameterized convection are more similar to each other, except that models with explicit convection again have more net radiation into the atmosphere than those with parameterized convection (R_TOA_ = 71.60 W m^−2^ vs. 61.34 W m^−2^), which is due to greater top of atmosphere absorbed solar radiation (ASR = 339.19 W m^−2^ vs. 327.28 W m^−2^), and again have a higher fraction of condensed water in ice form (IWP/(LWP + IWP) = 0.60 vs. 0.34).

In terms of the differences between pairs of RCE_small300 and RCE_large300 simulations (Table A3), models with parameterized convection have a greater average increase in atmospheric cooling, OLR, latent heat fluxes, precipitation, and lapse rate with aggregation than models with explicit convection (ΔR_Net_ = −31.90 W m^−2^ vs. −18.80 W m^−2^, ΔOLR = 32.29 W m^−2^ vs. 19.63 W m^−2^, ΔLHF = 30.12 W m^−2^ vs. 18.70 W m^−2^, ΔPrecip = 1.0 mm day^−1^ vs. 0.7 mm day^−1^, ΔLapse Rate = 0.45 K km^−1^ vs. 0.31 K km^−1^). This suggests that models with parameterized convection may be, on average, more aggregated than those with explicit convection. However, this is not apparent from the metrics of aggregation presented in section 5, so it is perhaps instead that models with parameterized convection have a greater mean state response to the same amount of aggregation. It also may be possible that SCMs are not consistent with a statistical ensemble of randomly convecting GCM grid columns, and so the difference between a SCM RCE_small simulation and a GCM RCE_large simulation may not solely reflect aggregation.

## Appendix B Aggregation Metrics

### B.1

*I*_*org*_ is calculated as in Tompkins and Semie (2017) where deep convective entities are identified as four‐point connected convective pixels. Deep convective pixels are defined as those with an OLR value less than 173 W m^−2^ corresponding to cold cloud top temperatures (Wing et al., 2018), as opposed to the previously used updraft threshold. The cumulative distribution function (CDF) of the nearest neighbor distances of the centroids of these convecting entities is plotted against the theoretical CDF of a two‐dimensional Poisson point process, with the area under this curve defining *I*_*org*_. Since an aggregated state has convective entities clustered in limited areas of the domain instead of randomly occurring over the whole domain, the CDF is skewed toward smaller nearest neighbor distances. Therefore, an *I*_*org*_ value greater than 0.5 indicates that convection is clustered, while *I*_*org*_= 0.5 indicates the convection is randomly distributed and an *I*_*org*_ value less than 0.5 indicates that convection is regularly distributed. To account for the geometry of a sphere, the calculation of *I*_*org*_ in the global simulations is limited to the tropical band, 30°S–30°N. In the supporting information, we discuss the sensitivity of using four‐point connectivity versus zero connectivity for defining convective entities in the GCMs (Figures S22 and S23) as well as the sensitivity of the calculation to various assumptions applied to the CDF calculation (Text S2). While the absolute value of *I*_*org*_ is affected by the choice of connectivity, its dependence on SST and the intermodel spread are not. Values of *I*_*org*_ are also sensitive to choices such as how to compute the empirical CDF; while most of these differences are small, they are important to consider if it is desired to reproduce identical results.

Subsidence fraction (*f*_*sub*_) is the area fraction of the domain where the daily‐average large‐scale vertical velocity at 500 hPa is directed downward. Vertical velocity is first averaged in time over a day, and in space over ∼100 × ∼100 km^2^ blocks. For CRMs, the RCE_large domain, which is roughly 6,000 × 400 km^2^, is divided into 60 blocks on the long side and four blocks on the short side. When the domain size *s* is not divisible by the number of blocks *k*, then some blocks have one grid point more than the others. The index that follows when rounding *s*/*k*i* down belongs to block *i*, the rounded up index belongs to block *i* + 1. This method minimizes the difference in box size across models, which is important because subsidence fraction is sensitive to the area over which vertical velocity is averaged (Cronin & Wing, 2017), though the tendency with temperature is less sensitive than the absolute values in any one simulation. The resulting boxes are roughly 100 × 100 km^2^ (the smallest is 96 × 96 km^2^ while the largest is 108 × 108 km^2^). For the GCRMs the same approach is used, except we divide the global domain in boxes that would represent a 1 × 1° grid on a full‐size globe, which is why we use 90 × 45 blocks in NICAM and SAM_GCRM and 45 × 23 blocks in MPAS. The GCMs already are on a coarse grid and are not further smoothed in space.

## References

[jame21181-bib-0001] Arnold, N. P. , & Randall, D. A. (2015). Global‐scale convective aggregation: Implications for the Madden‐Julian Oscillation. Journal of Advances in Modeling Earth Systems, 7, 1499–1518. 10.1002/2015MS000498

[jame21181-bib-0002] Bazile, E. , Couvreux, F. , Moigne, P. L. , Genthon, C. , Holtslag, A. A. M. , & Svensson, G. (2014). GABLS4: An intercomparison case to study the stable boundary layer over the Antarctic plateau. Global Energy Water Cycle Experiment News, 24(4), 4–4.

[jame21181-bib-0003] Becker, T. , Hohenegger, C. , & Stevens, B. (2017). Imprint of the convective parameterization and sea‐surface temperature on large‐scale convective self‐aggregation. Journal of Advances in Modeling Earth Systems, 9, 1488–1505. 10.1002/2016MS000865

[jame21181-bib-0004] Becker, T. , & Stevens, B. (2014). Climate and climate sensitivity to changing CO2 on an idealized land planet. Journal of Advances in Modeling Earth Systems, 6, 1205–1223. 10.1002/2014MS000369

[jame21181-bib-0006] Blossey, P. N. , Bretherton, C. S. , & Wyant, M. C. (2009). Subtropical low cloud response to a warmer climate in a superparameterized climate model. Part II: Column modeling with a cloud resolving model. Journal of Advances in Modeling Earth Systems, 1, 8 10.3894/JAMES.2009.1.8

[jame21181-bib-0007] Blossey, P. N. , Bretherton, C. S. , Zhang, M. , Cheng, A. , Endo, S. , Heus, T. , Liu, Y. , Lock, A. P. , de Roode, S. R. , & Xu, K.‐M. (2013). Marine low cloud sensitivity to an idealized climate change: The CGILS LES intercomparison. Journal of Advances in Modeling Earth Systems, 5, 234–258. 10.1002/jame.20025

[jame21181-bib-0008] Bony, S. , Semie, A. , Kramer, R. J. , Soden, B. J. , Tompkins, A. M. , & Emanuel, K. A. (2020). Observed modulation of the tropical radiation budget by deep convective organization and lower‐tropospheric stability. AGU Advances. 10.1029/2019AV000155

[jame21181-bib-0009] Bony, S. , Stevens, B. , Coppin, D. , Becker, T. , Reed, K. A. , Voigt, A. , & Medeiros, B. (2016). Thermodynamic control of anvil cloud amount. Proceedings of the National Academy of Sciences, 113(32), 8927–8932. 10.1073/pnas.1601472113 PMC4987798 27412863

[jame21181-bib-0010] Bony, S. , Stevens, B. , Frierson, D. M. W. , Jakob, C. , Kageyam, M. , Pincus, R. , Shepherd, T. G. , Sherwood, S. C. , Siebesma, A. P. , Sobel, A. H. , Watanabe, M. , & Webb, M. J. (2015). Clouds, circulation and climate sensitivity. Nature Geoscience, 8, 261–268. 10.1038/ngeo2398

[jame21181-bib-0011] Bosveld, F. C. , Baas, P. , Steeneveld, G.‐J. , Holtslag, A. A. M. , Angevine, W. M. , Bazile, E. , de Bruijn, E. I. F. , Deacu, D. , Edwards, J. M. , Ek, M. , Larson, V. E. , Pleim, J. E. , Raschendorfer, M. , & Svensson, G. (2014). The third GABLS intercomparison case for evaluation studies of boundary‐layer models. Part B: Results and process understanding. Boundary‐Layer Meteorology, 152, 157–187. 10.1007/s10546-014-9919-1

[jame21181-bib-0012] Brenowitz, N. D. , Majda, A. J. , & Yang, Q. (2018). The multiscale impacts of organized convection in global 2‐D cloud‐resolving models. Journal of Advances in Modeling Earth Systems, 10, 2009–2025. 10.1029/2018MS001335

[jame21181-bib-0013] Bretherton, C. S. , Blossey, P. N. , & Khairoutdinov, M. (2005). An energy‐balance analysis of deep convective self‐aggregation above uniform SST. Journal of the Atmospheric Sciences, 62, 4237–4292. 10.1175/JAS3614.1

[jame21181-bib-0014] Brient, F. , & Bony, S. (2012). How may low‐cloud radiative properties simulated in the current climate influence low‐cloud feedbacks under global warming? Geophysical Research Letters, 39, L20807 10.1029/2012GL053265

[jame21181-bib-0015] Browning, K. A. , Betts, A. , Jonas, P. R. , Kershaw, R. , Manton, M. , Mason, P. J. , Miller, M. , Moncrieff, M. W. , Sundqvist, H. , Tao, W. K. , Tiedtke, M. , Hobbs, P. V. , Mitchell, J. , Raschke, E. , Steward, R. E. , & Simpson, J. (1993). The GEWEX Cloud System Study (GCSS). Bulletin of the American Meteorological Society, 74, 387–400.

[jame21181-bib-0016] Ceppi, P. , Brient, F. , Zelinka, M. D. , & Hartmann, D. L. (2017). Cloud feedback mechanisms and their representation in global climate models. WIREs Climate Change, 8, e465 10.1002/wcc.465

[jame21181-bib-0017] Cess, R. D. , & Potter, G. L. (1988). A methodology for understanding and intercomparing atmospheric climate feedback processes in general circulation models. Journal of Geophysical Research, 93, 8305–8314. 10.1029/JD093iD07p08305

[jame21181-bib-0018] Chen, Y.‐W. , Seiki, T. , Kodama, C. , Satoh, M. , Noda, A. T. , & Yamada, Y. (2016). High cloud responses to global warming simulated by two different cloud microphysics schemes implemented in the nonhydrostatic icosahedral atmospheric model (NICAM). Journal of Climate, 29, 5949–5964. 10.1175/JCLI-D-15-0668.1

[jame21181-bib-0019] Coppin, D. , & Bony, S. (2015). Physical mechanisms controlling the initiation of convective self‐aggregation in a General Circulation Model. Journal of Advances in Modeling Earth Systems, 7, 2060–2078. 10.1002/2015MS000571

[jame21181-bib-0020] Coppin, D. , & Bony, S. (2018). On the interplay between convective aggregation, surface temperature gradients, and climate sensitivity. Journal of Advances in Modeling Earth Systems, 10, 3123–3138. 10.1029/2018MS001406 31007836 PMC6472628

[jame21181-bib-0021] Cronin, T. W. , & Emanuel, K. A. (2013). The climate time scale in the approach to radiative‐convective equilibrium. Journal of Advances in Modeling Earth Systems, 5, 843–849. 10.1002/jame.20049

[jame21181-bib-0022] Cronin, T. W. , & Wing, A. A. (2017). Clouds, circulation, and climate sensitivity in a radiative‐convective equilibrium channel model. Journal of Advances in Modeling Earth Systems, 9, 2833–2905. 10.1002/2017MS001111

[jame21181-bib-0023] Cuxart, J. , Holtslag, A. A. M. , Beare, R. J. , Bazile, E. , Beljaars, A. , Cheng, A. , Conangla, L. , Ek, M. , Freedman, F. , Hamdi, R. , Kerstein, A. , Kitagawa, H. , Lenderink, G. , Lewellen, D. , Mailhot, J. , Mauritsen, T. , Perov, V. , Schayes, G. , Steeneveld, G.‐J. , Svensson, G. , Taylor, P. , Weng, W. , Wunsch, S. , & Xu, K.‐M. (2006). Single‐column model intercomparison for a stably stratified atmospheric boundary layer. Boundary Layer Meteorol., 118, 273–300.

[jame21181-bib-0024] de Roode, S. R. , Sandu, I. , Van Der Dussen, J. J. , Ackerman, A. S. , Blossey, P. , Jarecka, D. , Lock, A. , Siebesma, A. P. , & Stevens, B. (2016). Large‐eddy simulations of EUCLIPSE–GASS Lagrangian stratocumulus‐to‐cumulus transitions: Mean state, turbulence, and decoupling. Journal of the Atmospheric Sciences, 73(6), 2485–2508.

[jame21181-bib-0025] Dunion, J. P. (2011). Rewriting the climatology of the tropical North Atlantic and Caribbean Sea atmosphere. Journal of Climate, 24, 893–908. 10.1175/2010JCLI3496.1

[jame21181-bib-0026] Emanuel, K. (2019). Inferences from simple models of slow, convectively coupled processes. Journal of the Atmospheric Sciences, 76, 195–208.

[jame21181-bib-0027] Eyring, V. , Bony, S. , Meehl, G. A. , Senior, C. A. , Stevens, B. , Stouffer, R. J. , & Taylor, K. E. (2016). Overview of the Coupled Model Intercomparison Project Phase 6 (CMIP6) experimental design and organization. Geoscientific Model Development, 9(5), 1937–1958. 10.5194/gmd-9-1937-2016

[jame21181-bib-0028] Grabowski, W. , & Moncrieff, M. (2001). Large‐scale organization of tropical convection in two‐dimensional explicit numerical simulations. Quarterly Journal of the Royal Meteorological Society, 127, 445–468.

[jame21181-bib-0029] Grabowski, W. , & Moncrieff, M. (2002). Large‐scale organization of tropical convection in two‐dimensional explicit numerical simulations: Effects of interactive radiation. Quarterly Journal of the Royal Meteorological Society, 128, 2349–2375. 10.1256/qj.01.104

[jame21181-bib-0030] Grabowski, W. W. , Moncrieff, M. W. , & Kiehl, J. T. (1996). Long‐term behavior of precipitating tropical cloud systems: A numerical study. Quarterly Journal of the Royal Meteorological Society, 122, 1019–1042.

[jame21181-bib-0031] Haarsma, R. J. , Roberts, M. J. , Vidale, P. L. , Senior, C. A. , Bellucci, A. , Bao, Q. , Chang, P. , Corti, S. , Fučkar, N. S. , Guemas, V. , von Hardenberg, J. , Hazeleger, W. , Kodama, C. , Koenigk, T. , Leung, L. R. , Lu, J. , Luo, J.‐J. , Mao, J. , Mizielinski, M. S. , Mizuta, R. , Nobre, P. , Satoh, M. , Scoccimarro, E. , Semmler, T. , Small, J. , & von Storch, J.‐S. (2016). High Resolution Model Intercomparison Project (High ResMIP v1.0) for CMIP6. Geoscientific Model Development, 9, 4185–4208. 10.5194/gmd-9-4185-2016

[jame21181-bib-0032] Harrop, B. E. , & Hartmann, D. L. (2012). Testing the role of radiation in determining tropical cloud top temperature. Journal of Climate, 25, 5731–5747.

[jame21181-bib-0033] Hartmann, D. L. , & Larson, K. (2002). An important constraint on tropical cloud‐climate feedback. Geophysical Research Letters, 29(20), 1951 10.1029/2002GL015835

[jame21181-bib-0034] Held, I. M. (2005). The gap between simulation and understanding in climate modeling. Bulletin of the American Meteorological Society, 86, 1609–1614. 10.1175/bams-86-11-1609

[jame21181-bib-0035] Held, I. M. (2014). Simplicity amid complexity. Science, 343, 1206–1207. 10.1126/science.1248447 24626917

[jame21181-bib-0036] Held, I. M. , Hemler, R. S. , & Ramaswamy, V. (1993). Radiative‐convective equilibrium with explicit two‐dimensional moist convection. Journal of the Atmospheric Sciences, 50, 3909–3927.

[jame21181-bib-0037] Held, I. M. , Zhao, M. , & Wyman, B. (2007). Dynamic radiative‐convective equilibria using GCM column physics. Journal of the Atmospheric Sciences, 64, 228–238. 10.1175/JAS3825.11

[jame21181-bib-0038] Hohenegger, C. , & Stevens, B. (2016). Coupled radiative convective equilibrium simulationswith explicit and parameterized convection. Journal of Advances in Modeling Earth Systems, 8, 1468–1482. 10.1002/2016MS000666

[jame21181-bib-0039] Holloway, C. E. , & Woolnough, S. J. (2016). The sensitivity of convective aggregation to diabatic processes in idealized radiative‐convective equilibrium simulations. Journal of Advances in Modeling Earth Systems, 8, 166–195. 10.1002/2015MS000511

[jame21181-bib-0040] Islam, S. , Bras, R. L. , & Emanuel, K. A. (1993). Predictability of mesoscale rainfall in the tropics. Journal of Applied Meteorology, 32, 297–310.

[jame21181-bib-0041] Jeevanjee, N. , Hassanzadeh, P. , Hill, S. A. , & Sheshadri, A. (2017). A perspective on climate model hierarchies. Journal of Advances in Modeling Earth Systems, 9, 1760–1771. 10.1002/2017MS001038

[jame21181-bib-0042] Jeevanjee, N. , & Romps, D. M. (2013). Convective self‐aggregation, cold pools, and domain size. Geophysical Research Letters, 40, 994–998. 10.1002/grl/50204

[jame21181-bib-0043] Khairoutdinov, M. F. , & Emanuel, K. (2013). Rotating radiative‐convective equilibrium simulated by a cloud‐resolving model. Journal of Advances in Modeling Earth Systems, 5, 816–825. 10.1002/2013MS000253

[jame21181-bib-0044] Kluft, L. , Dacie, S. , Buehler, S. A. , Schmidt, H. , & Stevens, B. (2019). Re‐examining the first climate models: Climate sensitivity of a modern radiative‐convective equilbrium model. Journal of Climate, 32, 8111–8125. 10.1175/JCLI-D-18-0774.1

[jame21181-bib-0045] Kuang, Z. , & Hartmann, D. L. (2007). Testing the fixed anvil temperature hypothesis in a cloud‐resolving model. Journal of Climate, 20, 2051–2057.

[jame21181-bib-0046] Möller, F. (1963). On influence of changes in CO_2_ concentration in air on radiation balance of Earth's surface and on climate. Journal of Geophysical Research, 68, 3877.

[jame21181-bib-0047] Maher, P. , Gerber, E. P. , Medeiros, B. , Merlis, T. M. , Sherwood, S. , Sheshadri, A. , Sobel, A. H. , Vallis, G. K. , Voigt, A. , & Zurita‐Gotor, P. (2019). Model hierarchies for understanding atmospheric circulation. Reviews of Geophysics, 57, 250–280. 10.1029/2018rg000607

[jame21181-bib-0048] Manabe, S. , & Strickler, R. F. (1964). Thermal equilibriation of the atmosphere with a convective adjustment. Journal of the Atmospheric Sciences, 21, 361–385. 10.1175/1520-0469(1964)021<0361:TEOTAW>2.0.CO;2

[jame21181-bib-0049] Mauritsen, T. , & Stevens, B. (2015). Missing iris effect as a possible cause of muted hydrological change and high climate sensitivity in models. Nature Geoscience, 8, 346–351.

[jame21181-bib-0050] Meehl, G. A. , Boer, G. J. , Covey, C. , Latif, M. , & Stouffer, R. J. (1997). Intercomparison makes for a better climate model. EOS Transactions American Geophysical Union, 78, 445–451.

[jame21181-bib-0051] Meehl, G. A. , Boer, G. J. , Covey, C. , Latif, M. , & Stouffer, R. J. (2000). The Coupled Model Intercomparison Project (CMIP). Bulletin of the American Meteorological Society, 81, 313–318.

[jame21181-bib-0052] Meehl, G. A. , Covey, C. , Taylor, K. E. , Delworth, T. , Stouffer, R. J. , Latif, M. , McAveney, B. , & Mitchell, J. F. B. (2007). The WCRP CMIP3 multimodel dataset: A new era in climate change research. Bulletin of the American Meteorological Society, 88(1383–1394).

[jame21181-bib-0053] Moeng, C.‐H. , Cotton, W. R. , Bretherton, C. , Chlond, A. , Khairoutdinov, M. , Krueger, S. , Lewellen, W. S. , MacVean, M. K. , Pasquier, J. R. M. , Rand, H. A. , Siebesma, A. P. , Stevens, B. , & Sykes, R. I. (1996). Simulation of a stratocumulus‐topped planetary boundary layer: intercomparison among different numerical codes. Bulletin of the American Meteorological Society, 77, 261–278.

[jame21181-bib-0054] Muller, C. J. , & Bony, S. (2015). What favors convective aggregation and why? Geophysical Research Letters, 42, 5626–5643. 10.1002/2015GL064260

[jame21181-bib-0055] Muller, C. J. , & Held, I. M. (2012). Detailed investigation of the self‐aggregation of convection in cloud resolving simulations. Journal of the Atmospheric Sciences, 69, 2551–2565. 10.1175/JAS-D-11-0257.1

[jame21181-bib-0056] Nakajima, K. , & Matsuno, T. (1988). Numerical experiments concerning the origin of cloud clusters in the tropical atmospheres. Journal of the Meteorological Society of Japan, 66, 309–329.

[jame21181-bib-0057] Neggers, R. A. J. , Ackerman, A. S. , Angevine, W. M. , Bazile, E. , Beau, I. , Blossey, P. N. , Boutle, I. A. , Bruijn, C. D. , Cheng, A. , van der Dussen, J. , Fletcher, J. , Dal Gesso, S. , Jam, A. , Kawai, H. , Cheedela, S. K. , Larson, V. E. , Lefebvre, M.‐P. , Lock, A. P. , Meyer, N. R. , de Roode, S. R. , de Rooy, W. , Sandu, I. , Xiao, H. , & Xu, K.‐M. (2017). Single‐column model simulations of subtropical marine boundary‐layer cloud transitions under weakening inversions. Journal of Advances in Modeling Earth Systems, 9, 2385–2412. 10.1002/2017MS001064

[jame21181-bib-0058] Ohno, T. , & Satoh, M. (2018). Roles of cloud microphysics on cloud responses to sea surface temperatures in radiative‐convective equilibrium experiments using a high‐resolution global nonhydrostatic model. Journal of Advances in Modeling Earth Systems, 10, 1970–1989. 10.1029/2018MS001386

[jame21181-bib-0059] Ohno, T. , Satoh, M. , & Noda, A. T. (2019). Fine vertical resolution radiative‐convective equilibrium experiments: Roles of turbulent mixing on the high‐cloud response to sea surface temperatures. Journal of Advances in Modeling Earth Systems, 11, 1637–1654. 10.1029/2019MS001704

[jame21181-bib-0060] Popke, D. , Stevens, B. , & Voigt, A. (2013). Climate and climate change in a radiative‐convective equilibrium version of ECHAM6. Journal of Advances in Modeling Earth Systems, 5, 1–14. 10.1029/2012MS000191

[jame21181-bib-0061] Randall, D. A. , Hu, Q. , Xu, K.‐M. , & Krueger, S. K. (1994). Radiative‐convective disequilibrium. Atmospheric Research, 31, 315–327.

[jame21181-bib-0062] Reed, K. A. , & Medeiros, B. (2016). A reduced complexity framework to bridge the gap between AGCMs and cloud‐resolving models. Geophysical Research Letters, 43, 860–866. 10.1002/2015GL066713

[jame21181-bib-0063] Reed, K. A. , Medeiros, B. , Bacmeister, J. T. , & Lauritzen, P. H. (2015). Global radiative‐convective equilibrium in the Community Atmosphere Model 5. Journal of the Atmospheric Sciences, 72, 2183–2197. 10.1175/JAS-D-14-0268.1

[jame21181-bib-0064] Romps, D. M. (2014). An analytical model for tropical relative humidity. Journal of Climate, 27, 7432–7449. 10.1175/JCLI-D-14-00255.1

[jame21181-bib-0065] Romps, D. M. (2016). Clausius‐Clapeyron scaling of CAPE from analytical solutions to RCE. Journal of the Atmospheric Sciences, 73, 3719–3737.

[jame21181-bib-0066] Seeley, J. T. , Jeevanjee, N. , Langhans, W. , & Romps, D. M. (2019). Formation of tropical anvil clouds by slow evaporation. Geophysical Research Letters, 46, 492–501. 10.1029/2018gl080747

[jame21181-bib-0067] Seeley, J. T. , Jeevanjee, N. , & Romps, D. M. (2019). FAT or FiTT: Are anvil clouds or the tropopause temperature invariant? Geophysical Research Letters, 46, 1842–1850. 10.1029/2018gl080096

[jame21181-bib-0068] Seeley, J. T. , & Romps, D. M. (2015). Why does tropical convective available potential energy (CAPE) increase with warming? Geophysical Research Letters, 42, 10,429–10,437. 10.1002/2015GL066199

[jame21181-bib-0069] Seidel, S. D. , & Yang, D. (2020). The lightness of water vapor helps to stabilize tropical climate. Science Advances, 6, eaba1951. 32494724 10.1126/sciadv.aba1951 PMC7202867

[jame21181-bib-0070] Silvers, L. G. , Stevens, B. , Mauritsen, T. , & Giorgetta, M. (2016). Radiative convective equilibrium as a framework for studying the interaction between convection and its large‐scale environment. Journal of Advances in Modeling Earth Systems, 8, 1330–1344. 10.1002/2016MS000629

[jame21181-bib-0071] Singh, M. S. , & O'Gorman, P. (2013). Influence of entrainment on the thermal stratification in simulations of radiative‐convective equilibrium. Geophysical Research Letters, 40, 4398–4403. 10.1002/glr.50796

[jame21181-bib-0072] Singh, M. S. , & O'Gorman, P. (2015). Increases in moist‐convective updraft velocities with warming in radiative‐convective equilibrium. Quarterly Journal of the Royal Meteorological Society, 141, 2828–2838. 10.1002/qj.2567

[jame21181-bib-0073] Singh, M. S. , Warren, R. A. , & Jakob, C. J. (2019). A steady‐state model for the relationship between humidity, instability, and precipitation in the tropics. Journal of Advances in Modeling Earth Systems, 11, 3973–3994. 10.1029/2019MS001686

[jame21181-bib-0074] Soden, B. J. , Broccoli, A. J. , & Hemler, R. S. (2004). On the use of cloud forcing to estimate cloud feedback. Journal of Climate, 17, 3661–3665.

[jame21181-bib-0075] Soden, B. J. , Held, I. M. , Colman, R. , Shell, K. M. , Kiehl, J. T. , & Shields, C. A. (2008). Quantifying climate feedbacks using radiative kernels. Journal of Climate, 21, 3504–3520.

[jame21181-bib-0076] Stephens, G. L. , van den Heever, S. , & Pakula, L. (2008). Radiative‐convective feedbacks in idealized states of radiative‐convective equilibrium. Journal of the Atmospheric Sciences, 65, 3899–3916. 10.1175/2008JAS2524.1

[jame21181-bib-0077] Stevens, B. , Satoh, M. , Auger, L. , Biercamp, J. , Bretherton, C. S. , Chen, X. , Díben, P. , Judt, F. , Khairoutdinov, M. , Klocke, D. , Kodama, C. , Kornblueh, L. , Lin, S.‐J. , Neumann, P. , Putman, W. M. , Röber, N. , Shibuya, R. , Vanniere, B. , Vidale, P. L. , Wedi, N. , & Zhou, L. (2019). DYAMOND: The DYnamics of the Atmospheric general circulation Modeled On Non‐hydrostatic Domains. Progress in Earth and Planetary Science, 6(1), 61 10.1186/s40645-019-0304-z

[jame21181-bib-0078] Sui, C. H. , Lau, K. M. , Tao, W. K. , & Simpson, J. (1994). The tropical water and energy cycles in a cumulus ensemble model. Part I: Equilibrium climate. Journal of the Atmospheric Sciences, 51, 711–728.

[jame21181-bib-0079] Svensson, G. A. A. M. H. , Kumar, V. , Mauritsen, T. , Steeneveld, G. J. , Angevine, W. M. , Bazile, E. , Beljaars, A. , de Bruijn, E. I. F. , Cheng, A. , Conangla, L. , Cuxart, J. , Ek, M. , Falk, M. J. , Freedman, F. , Kitagawa, H. , Larson, V. E. , Lock, A. , Masson, J. V. , Park, S. , Pleim, J. , Söderberg, S. , Weng, W. , & Zampieri, M. (2011). Evaluation of the diurnal cycle in the atmospheric boundary layer over land as represented by a variety of single‐column models: The second GABLS experiment. Boundary‐Layer Meteorology, 140(2), 177–206. 10.1007/s10546-011-9611-7

[jame21181-bib-0080] Taylor, K. E. , Stouffer, R. J. , & Meehl, G. A. (2012). An overview of CMIP5 and the experiment design. Bulletin of the American Meteorological Society, 93, 485–498. 10.1175/BAMS-D

[jame21181-bib-0081] Tobin, I. , Bony, S. , & Roca, R. (2012). Observational evidence for relationships between the degree of aggregation of deep convection, water vapor, surface fluxes, and radiation. Journal of Climate, 25, 6885–6904. 10.1175/jcli-d-11-00258.1

[jame21181-bib-0082] Tompkins, A. M. , & Craig, G. C. (1998a). Radiative‐convective equilibrium in a three‐dimensional cloud‐ensemble model. Quarterly Journal of the Royal Meteorological Society, 124, 2073–2097.

[jame21181-bib-0083] Tompkins, A. M. , & Craig, G. C. (1998b). Time? scales of adjustment to radiative‐convective equilibrium in the tropical atmosphere. Quarterly Journal of the Royal Meteorological Society, 124, 2693–2713. 10.1002/qj.49712455208

[jame21181-bib-0084] Tompkins, A. M. , & Craig, G. C. (1999). Sensitivity of tropical convection to sea surface temperature in the absence of large‐scale flow. Journal of Climate, 12, 462–476.

[jame21181-bib-0085] Tompkins, A. M. , & Semie, A. G. (2017). Organization of tropical convection in low vertical wind shears: Role of updraft entrainment. Journal of Advances in Modeling Earth Systems, 9, 1046–1068. 10.1002/2016MS000802

[jame21181-bib-0086] Tsushima, Y. , Iga, S. , Tomita, H. , Satoh, M. , Noda, A. T. , & Webb, M. J. (2014). High cloud increase in a perturbed SST experiment with a global nonhydrostatic model including explicit convective processes. Journal of Advances in Modeling Earth Systems, 6, 571–585. 10.1002/2013MS000301

[jame21181-bib-0087] Ullrich, P. A. , Jablonowski, C. , Kent, J. , Lauritzen, P. H. , Nair, R. , Reed, K. A. , Zarzycki, C. M. , Hall, D. M. , Dazlich, D. , Heikes, R. , Konor, C. , Randall, D. , Dubos, T. , Meurdesoif, Y. , Chen, X. , Harris, L. , Kühnlein, C. , Lee, V. , Qaddouri, A. , Girard, C. , Giorgetta, M. , Reinert, D. , Klemp, J. , Park, S.‐H. , Skamarock, W. , Miura, H. , Ohno, T. , Yoshida, R. , Walko, R. , Reinecke, A. , & Viner, K. (2017). DCMIP2016: A review of non‐hydrostatic dynamical core design and intercomparison of participating models. Geoscientific Model Development, 10(12), 4477–4509. 10.5194/gmd-10-4477-2017

[jame21181-bib-0088] van den Heever, S. C. , Stephens, G. L. , & Wood, N. B. (2011). Aerosol indirect effects on tropical convection characteristics under conditions of radiative‐convective equilibrium. Journal of the Atmospheric Sciences, 68, 699–718. 10.1175/2010JAS3603.1

[jame21181-bib-0089] Vial, J. , Vogel, R. , Bony, S. , Stevens, B. , Winker, D. M. , Cai, X. , Hohenegger, C. , Naumann, A. K. , & Brogniez, H. (2019). A new look at the daily cycle of trade wind cumuli. journal of advances in modeling Earth systems. Journal of Advances in Modeling Earth Systems, 11, 3148–3166. 10.1029/2019MS001746 31894190 PMC6919927

[jame21181-bib-0090] Voigt, A. , Biasutti, M. , Scheff, J. , Bader, J. , Bordoni, S. , Codron, F. , Dixon, R. D. , Jonas, J. , Kang, S. M. , Klingaman, N. P. , Leung, R. , Lu, J. , Mapes, B. , Maroon, E. A. , McDermid, S. , Park, J. , Roehrig, R. , Rose, B. E. J. , Russell, G. L. , Seo, J. , Toniazzo, T. , Wei, H.‐H. , Yoshimori, M. , & Vargas Zeppetello, L. R. (2016). The tropical rain belts with an annual cycle and a continent model intercomparison project: TRACMIP. Journal of Advances in Modeling Earth Systems, 8, 1868–1891. 10.1002/2016MS000748 32850005 PMC7447145

[jame21181-bib-0091] Wagner, W. , & Pruß, A. (2002). The IAPWS formulation 1995 for the thermodynamic properties of ordinary water substance for general and scientific use. Journal of Physical and Chemical Reference Data, 31, 387–535.

[jame21181-bib-0092] Wagner, W. , Riethmann, T. , Feistel, R. , & Harvey, A. H. (2011). New equations for the sublimation pressure and melting pressure of H_2_O ice Ih. Journal of Physical and Chemical Reference Data, 40, 43103 10.1063/1.3657937

[jame21181-bib-0093] Webb, M. J. , Andrews, T. , Bodas‐Salcedo, A. , Bony, S. , Bretherton, C. S. , Chadwich, R. , Chepfer, H. , Douville, H. , Good, P. , Kay, J. E. , Klein, S. A. , Marchand, R. , Medeiros, B. , Siebesma, A. P. , Skinner, C. B. , Stevens, B. , Tselioudis, G. , Tsushima, Y. , & Watanabe, M. (2017). The Cloud Feedback Model Intercomparison Project (CFMIP) contribution to CMIP6. Geoscientific Model Development, 10, 359–384. 10.5194/gmd-10-359-2017

[jame21181-bib-0094] Wing, A. A. (2019). Self‐aggregation of deep convection and its implications for climate. Current Climate Change Reports, 5, 1–11. 10.1007/s40641-019-00120-3

[jame21181-bib-0095] Wing, A. A. , & Cronin, T. W. (2016). Self‐aggregation of convection in long channel geometry. Quarterly Journal of the Royal Meteorological Society, 142, 1–15. 10.1002/qj.2628

[jame21181-bib-0096] Wing, A. A. , & Emanuel, K. A. (2014). Physical mechanisms controlling self‐aggregation of convection in idealized numerical modeling simulations. Journal of Advances in Modeling Earth Systems, 6, 59–74. 10.1002/2013MS000269

[jame21181-bib-0097] Wing, A. A. , Emanuel, K. , Holloway, C. E. , & Muller, C. (2017). Convective self‐aggregation in numerical simulations: A review. Surveys in Geophysics, 38(6), 1173–1197. 10.1007/s10712-017-9419-1 PMC6956938 31997841

[jame21181-bib-0098] Wing, A. A. , Reed, K. A. , Satoh, M. , Stevens, B. , Bony, S. , & Ohno, T. (2018). Radiative‐Convective Equilibrium Model Intercomparison Project. Geoscientific Model Development, 11, 793–813. 10.5194/gmd-11-793-2018

[jame21181-bib-0099] Yang, D. (2018a). Boundary‐layer diabatic processes, the virtual effect, and convective self‐aggregation. Journal of Advances in Modeling Earth Systems, 10, 2163–2176. 10.1029/2017MS001261

[jame21181-bib-0100] Yang, D. (2018b). Boundary layer height and buoyancy determine the horizontal scale of convective self‐aggregation. Journal of the Atmospheric Sciences, 75(2), 469–478. 10.1175/JAS-D-17-0150.1

[jame21181-bib-0101] Zelinka, M. D. , & Hartmann, D. L. (2010). Why is longwave cloud feedback positive? Journal of Geophysical Research, 115, D16117 10.1029/2010JD013817

[jame21181-bib-0102] Zhang, M. , Bretherton, C. S. , Blossey, P. N. , Austin, P. H. , Bacmeister, J. T. , Bony, S. , Brient, F. , Cheedela, S. K. , Cheng, A. , Genion, A. D. D. , Roode, S. R. D. , Endo, S. , Franklin, C. N. , Golaz, J.‐C. , Hannay, C. , Heus, T. , Isotta, F. A. , Dufresne, J.‐L. , Kang, I.‐S. , Kawai, H. , Kohler, M. , Larson, V. E. , Liu, Y. , Lock, A. P. , Lohmann, U. , Khairoutdinov, M. F. , Molod, A. M. , Neggers, R. A. J. , Rasch, P. , Sandu, I. , Senkbeil, R. , Siebesma, A. P. , Siegenthaler‐LeDrian, C. , Stevens, B. , Suarez, M. J. , Xu, K.‐M. , von Salzen, K. , Wolf, A. , & Zhao, M. (2013). CGILS: Results from the first phase of an international project to understand the physical mechanisms of low cloud feedbacks in single column models. Journal of Advances in Modeling Earth Systems, 5, 826–842. 10.1002/2013MS000246

[jame21181-bib-0103] Zhang, M. , Bretherton, C. S. , Blossey, P. N. , Bony, S. , Brient, F. , & Golaz, J.‐C. (2012). The CGILS experimental design to investigate low cloud feedbacks in general circulation models by using single–column and large‐eddy simulation models. Journal of Advances in Modeling Earth Systems, 4, M12001 10.1029/2012MS000182

