# Supplementary material for: Clouds and Convective Self‐Aggregation in a Multimodel Ensemble of Radiative‐Convective Equilibrium Simulations
Source: J Adv Model Earth Syst. 2020 Sep 18;12(9):e2020MS002138. doi: 10.1029/2020MS002138 (PMC7539986; doi:10.1029/2020MS002138)
Supplement: Supplementary file 1 — Supporting Information S1 [file JAME-12-e2020MS002138-s001.pdf]

Supporting Information for "Clouds and Convective Self-Aggregation in a Multi-Model Ensemble of Radiative-Convective Equilibrium Simulations"

Allison A. Wing[1], Catherine L. Stauffer[1], Tobias Becker[2], Kevin A. Reed[3], Min-Seop Ahn[4], Nathan P. Arnold[5], Sandrine Bony[6], Mark Branson[7], George H. Bryan[8], Jean-Pierre Chaboureau[9], Stephan R. de Roode[10], Kulkarni Gayatri[11], Cathy Hohenegger[2], I-Kuan Hu[12], Fredrik Jansson[10,13], Todd R. Jones[14], Marat Khairoutdinov[15], Daehyun Kim[4], Zane K. Martin[16], Shuhei Matsugishi[17], Brian Medeiros[7], Hiroaki Miura[18], Yumin Moon[4], Sebastian K. Müller[2], Tomoki Ohno[19], Max Popp[20], Thara Prabhakaran[11], David Randall[7], Rosimar Rios-Berrios[8], Nicolas Rochetin[2,20], Romain Roehrig[22], David M. Romps[23,24], James H. Ruppert, Jr.[25], Masaki Satoh[17], Levi G. Silvers[3], Martin S. Singh[26], Bjorn Stevens[2], Lorenzo Tomassini[27], Chiel C. van Heerwaarden[28], Shuguang Wang[16], Ming Zhao[29]

[1]Department of Earth, Ocean and Atmospheric Science, Florida State University, Tallahassee, FL, USA

[2]Max Planck Institute for Meteorology, Hamburg, Germany

[3]School of Marine and Atmospheric Sciences, Stony Brook University, Stony Brook, NY, USA

[4]Department of Atmospheric Sciences, University of Washington, Seattle, WA, USA

[5]Global Modeling and Assimilation Office, NASA Goddard Space Flight Center, Greenbelt, MD, USA

[6]Laboratoire de Météorologie Dynamique (LMD)/IPSL/Sorbonne Université/CNRS, Paris, France

[7]Department of Atmospheric Science, Colorado State University, Fort Collins, CO, USA

[8]National Center for Atmospheric Research, Boulder, CO, USA

[9]Laboratoire d'Aérologie, Université de Toulouse, CNRS, UPS, Toulouse, France

[10]Delft University of Technology, Delft, The Netherlands

[11]Indian Institute of Tropical Meteorology, Pune, India

[12]Rosenstiel School of Marine and Atmospheric Science, University of Miami, Miami, FL, USA

[13]Centrum Wiskunde & Informatica, Amsterdam, The Netherlands

[14]Department of Meteorology, University of Reading, Reading, UK

[15]School of Marine and Atmospheric Sciences, and Institute for Advanced Computational Science, Stony Brook University, State University of New York, Stony Brook, NY, USA

[16]Department of Applied Physics and Applied Mathematics, Columbia University, New York, NY, USA

[17]Atmosphere and Ocean Research Institute, The University of Tokyo, Kahsiwa, Japan

[18]Department of Earth and Planetary Science, Graduate School of Science, The University of Tokyo, Tokyo, Japan

[19]Japan Agency for Marine-Earth Science and Technology, Yokohama, Japan

[20]Laboratoire de Météorologie Dynamique (LMD)/IPSL/Sorbonne Université/CNRS/École Polytechnique/École Normale Supérieure, Paris, France

[21]Laboratoire de Météorologie Dynamique, Paris, France

[22]CNRM, Université de Toulouse, Météo-France, CNRS, Toulouse, France

[23]Department of Earth and Planetary Science, University of California, Berkeley, CA, USA

[24]Climate and Ecosystem Sciences Division, Lawrence Berkeley National Laboratory, Berkeley, CA, USA

[25]Department of Meteorology and Atmospheric Science and Center for Advanced Data Assimilation and Predictability Techniques, The Pennsylvania State University, University Park, PA, USA

[26]School of Earth, Atmosphere and Environment, Monash University, Victoria, Australia

[27]Met Office, Exeter, UK

[28]Meteorology and Air Quality Group, Wageningen University, Wageningen, The Netherlands

[29]NOAA/Geophysical Fluid Dynamics Laboratory, Princeton, NJ, USA

Contents of this file

1. Text S1-S2
2. Captions for Movies S1-S65
3. Tables S1-S13
4. Figures S1-S28

Introduction This supporting information contains the documentation for each model included in RCEMIP (Text S1), information on the sensitivity of the I_{org} calculation to the assumptions in those calculation (Text S2, Table S1), as well as captions for Movies S1 to S65. Tables S2-S7 provide the anvil cloud fractions, temperatures, and heights for each simulation. Tables S8-S13 provide calculations of the net climate feedback parameter and change in cloud radiative effect in each model.

This supporting information also includes additional figures to provide a complete view of the RCEMIP simulations. A series of figures shows snapshots of hourly-averaged precipitable water during the equilibrium state in each model in the simulations at 300 K (Figures S1-S5). The precipitable water is spatially homogenous in Figures S1 and S2, reflecting that the `RCE_small300`, `RCE_small_vert300`, and `RCE_smal_les300` simulations are un-aggregated (except UKMO-RA1-T), while there are regions of moister and drier air in Figures S3-S5, reflecting that the `RCE_large300` simulations are aggregated. Figures S6-S8 display the temporal evolution of y-averaged precipitable water, showing the rich structure of variability across scales. Figure S9 shows snapshots of hourly-averged outgoing longwave radiation in each GCM in the simulations at 300 K over a subset of the global domain that approximately matches the size of the CRM simulations. Figure S10 shows the precipitable water distribution in all simulations, in which the `RCE_large` simulations have a strikingly broader distribution of precipitable water than the `RCE_small` simulations.

Figures S11-S14 display average profiles for the simulations at 295 K, while Figures S15-S18 display average profiles for the simulations at 305 K. The equivalent profiles for simulations at 300 K are in the main text. The next set of figures display measures of the degree of aggregation. Figures S19-S20 show the metrics of aggregation for `RCE_large295` and `RCE_large305` (`RCE_large300` is in the main text). Figure S21 shows the rate of change of the aggregation metrics with warming from 295K-300K and 300K-305K. Figures S22 and S23 show aggregation metrics in `RCE_large300` and their rate of change when I_{org} is calculated using zero instead of four-point connectivity. Figures S24 and S25 show aggregation metrics in `RCE_large300` and their rate of change when the spatial variance of precipitable water scaled by its domain mean value is used as a moisture-based metric

---

Corresponding author: A. A. Wing, Department of Earth, Ocean and Atmospheric Science, Florida State University, 1011 Academic Way, Tallahassee, FL 32306, USA. (awing@fsu.edu)

rather than the spatial variance of column relative humidity (as the latter may suffer from artifacts due to different specifications of vertical levels across models).

Figures S26-S28 display anvil cloud height, temperature, and fraction as a function of SST in which the models are split into those with explicit and parameterized convection. The equivalent plots for the entire ensemble of models are shown in the main text.

Text S1. Model Documentation

Here we provide information about each model included in RCEMIP, including information about the dynamical core, physics packages, and grid settings for the `RCE_small`, `RCE_large`, and (if applicable) `RCE_small_vert` and `RCE_small_les`.

S1.1. CRM/LES Models

S1.1.1. CM1

CM1 (cm1r19.6; Bryan & Morrison, 2012) is used to perform both CRM and LES simulations. CM1 employs a Cartesian grid with Arakawa-C staggering and a compressible time-split, RK3, finite difference dynamical core (Wicker & Skamarock, 2002). The default advection schemes for CM1 are used (a 5th-order finite difference scheme for velocities and a 5th-order WENO scheme for scalars). The time step is variable with an average value of 18 s. `RCE_small` has 108 x 108 x 74 grid points with 1 km horizontal grid spacing. `RCE_large` has 2016 x 134 x 74 grid points with 3 km horizontal grid spacing. In both cases, the vertical levels are those specified by Wing et al. (2018). There is a sponge layer above 25 km. `RCE_small_vert` has 108 x 108 x 146 grid points with 1 km horizontal grid spacing while `RCE_small_les` has 540 x 540 x 146 grid points with 200 m horizontal grid spacing. The physics packages are RRTMG (Mlawer et al., 1997) for shortwave and longwave radiation, Morrison et al. (2009) double-moment for microphysics, and Jimenez et al. (2012) for the surface layer. No convection scheme is used. For CM1 and CM1-vert, the boundary layer scheme of Bryan and Rotunno (2009) is used, with no other sub-grid scale turbulence scheme. For CM1-LES, the Deardorff (1980) sub-grid scale turbulence scheme is used.

S1.1.2. DALES, DALES-damping

The Dutch Atmospheric Large-Eddy Simulation (DALES) model v4.2 (Heus et al., 2010) has a finite volume dynamical core, employs the anelastic approximation, and uses a Cartesian grid with an adaptive time step of $\sim$ 2 s. The `RCE_small` configuration has 100 x 100 x 74 grid points with 1 km horizontal grid spacing, `RCE_small_vert` has 100 x 100 x 146 grid points with 1 km horizontal grid spacing, and `RCE_small_les` has 504 x 504 x 146 grid points with 200 m grid spacing. The vertical levels are a specified by Wing et al. (2018) and there is a sponge layer in the top 25% levels. The physics packages are RRTMG for shortwave and longwave radiation (Mlawer et al., 1997), Grabowski (1998); Khairoutdinov and Randall (2006) for microphysics, Deardorff (1980) for sub-grid scale turbulence and MOST for calculating the surface fluxes. DALES-damping is identical to DALES, with the addition of a damping of layer-averaged horizontal velocity towards zero, with a damping constant of 1 d^{-1}. The damping is added to decrease the build-up of strong winds in the stratosphere.

S1.1.3. DAM

Das Atmosphaerische Modell (DAM; Romps, 2008) has a finite volume dynamical core and uses a Cartesian grid. The `RCE_small` configuration has 108 x 108 x 74 grid points

with 1 km horizontal grid spacing. RCE_large has 2592 x 144 x 74 grid points with 2.5 km horizontal grid spacing. The time step is variable between 7 and 20 s, depending on the CFL condition. The 74 vertical levels are stretched to a spacing of 500 m by 3 km, which is continued up to the model top of 33 km; there is no sponge layer. RRTMG is used for shortwave and longwave radiation and the Lin-Lord-Krueger scheme is used for microphysics. There is no sub-grid scale turbulence scheme and no boundary layer scheme; a bulk aerodynamic scheme is used for surface fluxes.

S1.1.4. FV3

The GFDL-FV3CRM (Lin, 2004; Putman & Lin, 2007; Harris & Lin, 2013; Zhou et al., 2019) has a finite volume dynamical core and uses a Cartesian grid with a time step of 75 s. The RCE_large configuration has 2048 x 128 x 79 grid points with 3 km horizontal grid spacing. There is a sponge layer in the top two levels. The physics packages are GFDL AM4 radiation, modified Lin microphysics (as in the GFDL microphysics in NGGPS), the GFDL AM4 PBL Lock scheme for boundary layer. There is no convection scheme and no sub-grid scale turbulence scheme. Output is not available for RCE_small.

S1.1.5. ICON-LEM

The ICOsahedral Nonhydrostatic model (ICON) version 2.3.00 (Dipankar et al., 2015) is used to perform both CRM and LES simulations. ICON-LEM employs an icoshedral-triangular Arakawa-C grid with a non-hydrostatic dynamical core. The edge of each triangle is 1 km in the RCE_small configuration and 3 km in the RCE_large configuration. RCE_small and RCE_large use 5 s and 10 s time steps, respectively. After remapping to a Cartesian grid, the RCE_small configuration has 100 x 100 x 75 grid points with a 1 km grid spacing and the RCE_large configuration has 134 x 2000 x 75 grid points with a 3 km grid spacing. The vertical levels are geopotential height levels and extend up to 33.947 km, with a sponge layer starting at 19 km. RCE_small_vert has 100 x 100 x 156 grid points while RCE_small_les has 500 x 500 x 156 grid points. The physics packages are RRTMG (Mlawer et al., 1997) for shortwave and longwave radiation (called every 300 s), Seifert and Beheng (2006) two-moment mixed-phase bulk scheme for microphysics, and Smagorinsky boundary layer and sub-grid scale turbulence schemes. The RCE_large simulations were initialized from RCE_small simulations at 3 km grid spacing.

S1.1.6. ICON-NWP

ICON-NWP (Zängl et al., 2015) is the same as ICON-LEM in terms of the dynamical core and grid configuration, but uses different boundary layer, turbulence and cloud schemes. The physics packages are RRTMG (Mlawer et al., 1997) for shortwave and longwave radiation (called every 300 s), Seifert and Beheng (2006) two-moment mixed-phase bulk scheme for microphysics, Raschendorfer (2001) boundary layer and sub-grid scale turbulence schemes, and a diagnostic cloud cover scheme by Martin Koehler. The RCE_large simulations were initialized from RCE_small simulations at 3 km grid spacing.

S1.1.7. MESONH

MESO-NH version 5.4.1 (Lac et al., 2018) employs the anelastic equation set on a fully staggered Cartesian Arakawa-C grid. RCE_small has 100 x 100 x 74 grid points with 1 km horizontal grid spacing. RCE_large has 2000 x 128 x 74 grid points with 3 km horizontal grid spacing. The vertical levels are those specified by Wing et al. (2018) with a sponge layer from 22 to 33 km. RCE_small_vert has 100 x 100 x 146 grid points with 1 km horizontal grid spacing. RCE_small_les has 500 x 500 x 146 grid points

with 200 m horizontal grid spacing.The physics packages include RRTMG for longwave radiation, Fouquart and Bonnel (1980) for shortwave radiation (which are called every 900 s), the Pinty and Jabouille (1998) single-moment mixed microphysics scheme, the Pergaud, Masson, Malardel, and Couvreux (2009) eddy diffusivity mass flux scheme for shallow convection, and a 4th-order centered advection scheme for momentum variables and a PPM scheme for other variables. The LES simulations use the Cuxart, Bougeault, and Redelsperger (2000) 1.5 order closure TKE scheme withe the Deardorff (1980) mixing length while the other simulations use the same scheme but with the mixing length of Bougeault and Lacarrere (1989).

S1.1.8. MicroHH

MicroHH version 2.0 (van Heerwaarden et al., 2017) is used for the `RCE_small` and LES simulations. It has a finite volume dynamical core and uses a Cartesian grid with a third-order Runge-Kutta time-stepping scheme and variable time step with CFL at 1.2. `RCE_small` has 96 x 96 x 72 grid points, `RCE_small_vert` has 96 x 96 x 144 grid points with 1 km horizontal resolution. `RCE_small_les` has 480 x 480 x 144 grid points with 200 m horizontal resolution. The vertical levels follow those specified by Wing et al. (2018) and for the RCEMIP LES configuration and there is a sponge layer at the top of the model. The physics packages include RRTMGP (Pincus et al., 2019) for shortwave and longwave radiation, NSW6 (Tomita, 2008) for microphysics, and a Smagorinsky Lilly scheme with stability effects for sub-grid scale turbulence.

S1.1.9. SAM-CRM

The System for Atmospheric Modeling (SAM) version 6.11.2 (Khairoutdinov & Randall, 2003) is used to perform both CRM and LES simulations. SAM employs the anelastic equations on a fully staggered Arakawa C-type Cartesian grid. `RCE_small` has 96 x 96 x 74 grid points with 1 km horizontal grid spacing and a 8 s time step. `RCE_large` has 2048 x 192 x 74 grid points with 3 km horizontal grid spacing and a 12 s time step. The vertical levels are those specified by Wing et al. (2018) with the model top at 33 km and Newtonian damping of prognostic variables in the upper third of the model domain. `RCE_small_vert` has 96 x 96 x 146 grid points with 1 km horizontal grid spacing while `RCE_small_les` has 480 x 480 x 146 grid points with 200 m horizontal grid spacing. The physics packages include RRTMG for shortwave and longwave radiation, the original SAM one-moment microphysics scheme (Khairoutdinov & Randall, 2003) and a Smagorinksy sub-grid scale turbulence scheme. The original SAM advection scheme based on Smolarkiewicz' MPDATA scheme with a monotonic corrector is used.

S1.1.10. SCALE

SCALE version 5.2.5 (Nishizawa et al., 2015; Sato et al., 2015) employs a fully compressible equation set on a finite volume dynamical core on an Arakawa C-type Cartesian grid. `RCE_small` has 96 x 96 x 74 grid points with 1 km horizontal grid spacing and a 6 s advection and physics time step. `RCE_large` has 2048 x 128 x 74 grid points with 3 km horizontal grid spacing and a 12 s advection and time step. The dynamical time step is 1/3 of the advection and physics time step. The vertical levels are those specified by Wing et al. (2018) with a sponge layer from 22 to 33 km, in which only the vertical wind is damped. The physics packages include mstrnX (Sekiguchi & Nakajima, 2008) for shortwave and longwave radiation, which is called every 300 s, the Tomita (2008) six-class one-moment bulk microphysics scheme, and the Mellor-Yamada-Nakanishi-Niino

(Nakanishi & Niino, 2004) boundary layer scheme. Surface fluxes are calculated from a bulk method using the universal function (Beljaars & Holtslag, 1991; D. K. Wilson, 2001) and sea surface roughness is parameterized by the Moon, Ginis, Hara, and Thomas (2007) method. A third order upwind difference advection scheme for both velocities and scalars was used.

S1.1.11. UCLA-CRM

The University of California, Los Angeles large-eddy simulation model (UCLA-LES) (Hohenegger & Stevens, 2016) employs finite difference equations on a Cartesian Arakawa-C grid with a third-order Runge-Kutta time-stepping scheme and dynamic time step based on the CFL criterium. `RCE_small` has 96 x 96 x 75 grid points with 1 km horizontal grid spacing. `RCE_large` has 1984 x 128 x 75 grid points with 3 km horizontal grid spacing. There is a sponge layer in the top six vertical levels. Shortwave and longwave radiation are computed as in Pincus and Stevens (2009), using the delta-four stream method and Monte Carlo sampling, but with updates to include cloud ice. The two-moment microphysics of Seifert and Beheng (2006) is used, which has cloud water, rain, ice snow, graupel, and hail as the hydrometeor species. A Smagorinsky sub-grid scale turbulence scheme is used; there is no boundary layer scheme.

S1.1.12. UKMO-CASIM

The UK Met Office idealized Model version 11.0 (Stratton et al., 2018) (UKMO-CASIM) has semi-implicit, semi-lagrangian (Wood et al., 2014) numerics and a Cartesian horizontal C-grid and vertical sigma-z C-P grid. `RCE_small` uses 100 x 100 x 98 grid points with 1 km horizontal grid spacing. `RCE_large` uses 2016 x 144 x 98 grid points with 3 km horizontal grid spacing. The vertical levels are step-stretched with a grid spacing of 60 m near the surface, increasing to a constant 250 m grid spacing between 5 and 13 km, and increasing to a constant 1000 m grid spacing from 30 to 40 km. There is a sponge layer that performs Rayleigh damping of prognostic variables, in which a timescale weight follows an exponential function of height from 24-40 km. The Suite of Community RAdiative Transfer codes based on Edwards and Slingo (SOCRATES) scheme (Edwards & Slingo, 1996; Manners et al., 2015) is used for shortwave and longwave radiation and the double-moment Cloud AeroSol Interacting Microphysics (CASIM; Shipway & Hill, 2012; Hill et al., 2015) is used for microphysics. The Lock, Brown, Bush, Martin, and Smith (2000) boundary layer scheme with updates from Walters et al. (2019) and a Smagorinksy (1963) sub-grid scale turbulence scheme is also used.

S1.1.13. UKMO-RA1-T, UKMO-RA1-T-hrad, UKMO-RA1-T-nocloud

UKMO-RA1-T is the same as UKMO-CASIM except for the microphysics scheme and the use of a sub-grid cloud scheme based on Smith (1990), with uniform critical relative humidity of 0.99. The microphysics scheme is a single-moment scheme from D. R. Wilson and Ballard (1999) but with updates as in Walters et al. (2019). The UKMO-RA1-T `RCE_small` simulations unexpectedly showed convective self-aggregation, so additional simulations (UKMO-RA1-T-hrad) were performed in which radiative heating tendencies were computed interactively but applied uniformly as domain-mean profiles to prevent aggregation; the `RCE_large` UKMO-RA1-T simulations were initialized from the un-aggregated UKMO-RA1-T-hrad simulations. Additional simulations (UKMO-RA1-T-nocloud) were performed in which the sub-grid cloud scheme was disabled.

S1.1.14. WRF-COL-CRM

The Weather Research and Forecasting model (WRF) v3.5.1 (Skamarock et al., 2008) modified for performing RCE/WTG simulations (Wang et al., 2016; Martin et al., 2019), the same as used for WRF-GCM, has a conservative-form finite difference dynamical core on a Cartesian grid. The `RCE_small` configuration has 100 x 100 x 74 grid points with 1 km horizontal grid spacing. The `RCE_large` configuration has 2000 x 134 x 74 grid points with 3 km horizontal grid spacing. The time step is 20 seconds. The vertical levels are eta levels with the model top at approximately 33 km and a sponge layer in the top 5 km. The physics packages used are RRTMG (Iacono et al., 2008) for shortwave and longwave radiation, the Morrison et al. (2009) double-moment microphysics scheme, the YSU boundary layer scheme (Noh et al., 2003; Hong et al., 2006), and a horizontal Smagorinsky first-order closure for sub-grid scale turbulence.

S1.1.15. WRF-CRM

The Weather Research and Forecasting model (WRF) v3.9.1 (Skamarock et al., 2008) was used to perform `RCE_small` simulations, with 100 x 100 x 74 grid points with 1 km horizontal grid spacing and a 6 s time step. For `RCE_large` simulations, 2000 x 133 x 74 grid points with 3 km horizontal grid spacing and a 10 s time step were employed. The model top is set at 33 km with a sponge layer depth of 5 km from the model top. The physics packages include the Goddard for shortwave and longwave radiation schemes (Chou & Suarez, 1999; Chou et al., 2001) and the WDM6 (Lim & Hong, 2010) double-moment cloud microphysics scheme. The YSU boundary layer scheme (Hong et al., 2006) coupled with Monin-Obukhov similarity (Jimenez et al., 2012) for the surface layer and 5-layer thermal diffusion scheme (Dudhia, 1996) are used as a surface option.

S1.2. GCRMs

S1.2.1. MPAS

The Model for Prediction Across Scales-Atmosphere version 6.1 (MPAS) (Skamarock et al., 2012) is a nonhydrostatic atmospheric model with a finite element dynamical core that is here used in its global spherical configuration. MPAS uses C-grid discretization on a Voronoi unstructured grid. The `RCE_small` configuration is a small sphere with a reduced Earth radius of $\sim$ 52km and 10,242 horizontal grid columns, which are interpolated to 180 x 90 latitude by longitude grid points in the output. The horizontal grid spacing is 1 km, which is interpolated to 2° in the output. The time step is 6 seconds. The `RCE_large` configuration is a sphere with reduced Earth radius of $R_E/8$ and 1,024,002 horizontal grid columns, which are interpolated to 900 x 1800 latitude by longitude grid points in the output. The horizontal grid spacing is 3 km, which is interpolated to 0.2° in the output. The time step is 15 seconds. Both `RCE_small` and `RCE_large` use 74 vertical levels as defined in (Wing et al., 2018), with a sponge layer above 25 km. The physics packages used are RRTMG (Mlawer et al., 1997; Iacono et al., 2008) for shortwave and longwave radiation, the WSM6 microphysics scheme (Hong et al., 2004), and the YSU boundary layer scheme (Noh et al., 2003).

S1.2.2. NICAM

The Non-hydrostatic Icosahedral Atmospheric Model (NICAM) version 16.3 (Satoh et al., 2014) employs the fully compressible equations on a spherical A-type grid, with time steps of < 8 s for dynamics, < 30 s for cloud microphysics, and 300 s for radiation. `RCE_small` has 128 x 128 x 117 grid points with a horizontal grid spacing of 1 km, while `RCE_large` has reduced Earth radius of $R_E/4$ with a horizontal grid spacing

of 3.5 km and 117 vertical levels. The vertical grid has stretched grid spacing with 200 m grid spacing in the free atmosphere, a model top at 33 km, and Newtonian damping of momentum and internal energy above 20 km. The physics packages include mstrnX for radiation (Sekiguchi & Nakajima, 2008) and the NICAM double moment microphysics (NDW6) scheme. The turbulent closure was calculated using level 2 of the Mellor-Yamada-Nakanishi-Niino scheme (Nakanishi & Niino, 2004) in both the planetary boundary layer and the free atmosphere. There is no convection scheme.

S1.2.3. SAM-GCRM

The global version of the System for Atmospheric Modeling (SAM) (Khairoutdinov & Randall, 2003) uses version 7.3 with a spherical latitude-longitude grid (Satoh et al., 2019). Like SAM-CRM, SAM-GCRM employs the same anelastic finite-difference equation set. The `RCE_large` configuration has 2304 x 1152 x 74 grid points with 0.15625° x 0.1545° grid spacing and a reduced Earth radius of $R_E/4$, which corresponds to a horizontal grid spacing of 4.25 km at the Equator. The time step is 12 s. There are rigid walls at 89 N and 89 S. The vertical levels are those specified by Wing et al. (2018), with a sponge layer starting at 20 km. The physics packages include RRTMG for shortwave and radiation, the original SAM one-moment microphysics scheme (Khairoutdinov & Randall, 2003) and a Smagorinksy sub-grid scale turbulence scheme. The `RCE_large` simulations are initialized from the corresponding `RCE_small` simulations with SAM-CRM.

S1.3. GCMs/SCMs

S1.3.1. CAM5-GCM

The Community Atmosphere Model version 5 (CAM5) (Neale et al., 2012) is configured here with the spectral element dynamical core (Lauritzen et al., 2018) on a spherical grid for the GCM for `RCE_large` and as the single column model SCAM5 with the Eulerian spectral transform dynamical core for `RCE_small`. The physics time step is 1800 s, the dynamical time step is 300 s, and the simulations are run for 1200 days. `RCE_small` is a single column with 30 vertical levels corresponding to a $\sim$ 320 km grid (T42), in which the time step is 1800 s for both physics and dynamics. `RCE_large` has 48602 columns but is interpolated to a latitude-longitude grid of 192 x 288 columns, with a horizontal grid spacing of 0.9 x 1.25 degrees. There are 30 vertical levels that employ a terrain-following hybrid vertical coordinate and a sponge layer in the top 3 levels. The physics packages used are RRTMG (Mlawer et al., 1997; Iacono et al., 2008) for radiation, Morrison and Gettelman (2008) for microphysics, Park and Bretherton (2009) for boundary layer, Zhang and McFarlane (1995) for convection, and Bretherton and Park (2009) for sub-grid scale turbulenece. There are a few parameters that are different for SCAM5 and CAM5 due to the different dynamical cores used - [cldfrc_sh1]: 0.07D0 vs. 0.04D0; [zmconv_ke]: 3.0E-6 vs. 5.0E-6.

S1.3.2. CAM6-GCM

The Community Atmosphere Model version 6 (CAM6) (`https://ncar.github.io/CAM/doc/build/html/index.html`) is configured here with the spectral element dynamical core (Lauritzen et al., 2018) on a spherical grid for the GCM for `RCE_large` and as the single column model SCAM6 with the Eulerian spectral transform dynamical core for `RCE_small`. The physics time step is 1800 s, the dynamical time step is 300 s, and the simulations are run for 1200 days. `RCE_small` is a single column with 30 vertical levels corresponding to a $\sim$ 320 km grid (T42) in which the time step is 1800 s for both physics

and dynamics. `RCE_large` has 48602 columns but is interpolated to a latitude-longitude grid of 192 x 288 columns, with a horizontal grid spacing of 0.9 x 1.25 degrees. There are 32 vertical levels that employ a terrain-following hybrid vertical coordinate and a sponge layer in the top 3 levels. The physics packages used are RRTMG (Mlawer et al., 1997) for radiation, Gettelman and Morrison (2015) for microphysics, Zhang and McFarlane (1995) for convection, and CLUBB (Bogenschutz et al., 2013) for boundary layer and sub-grid scale turbulence. There are a few parameters that are different for SCAM6 and CAM6 due to the different dynamical cores used - [cldfrc_sh1]: 0.07D0 vs. 0.04D0; [zmconv_ke]: 3.0E-6 vs. 5.0E-6; [cldfrc_premit]: 75000.0D0 vs. 25000.0D0.

S1.3.3. CNRM-CM6-1

The CNRM climate model CNRM-CM6-1 is based on the atmospheric component ARPEGE-Climat Version 6.3 (Roehrig & coauthors, 2020; Voldoire & coauthors, 2019). Its dynamical core is spectral for linear terms (T127 truncature, ∼150km horizontal grid spacing) while all non-linear terms are computed on the associated reduced Gaussian grid with a two time-level semi-Lagrangian semi-implicit scheme and a 15 min time step. The model vertical grid consists of 91 hybrid sigma-pressure levels between the surface and 1 Pa, with smaller grid spacing in the boundary layer. The CNRM-CM6-1 physics packages include RRTMG (Mlawer et al., 1997) for longwave radiation and Fouquart and Bonnel (1980) for shortwave radiation, the Lopez (2002) single-moment microphysics scheme, the Cuxart et al. (2000) 1.5 order turbulence scheme, coupled to the Bougeault (1981) cloud scheme and a convection scheme unified for shallow and deep convection following Piriou, Redelsperger, Geleyn, Lafore, and Guichard (2007) and Guérémy (2011).

S1.3.4. ECHAM6-GCM

ECHAM version 6.3.04p1 is the atmospheric component of the MPI-M Earth System Model (Stevens et al., 2013) and employs a dry spectral-transform dynamical core on a spherical grid with a time step of 450 s. `RCE_large` has 192 x 96 horizontal grid points with T63 resolution and 47 vertical levels, employing a hybrid sigma-pressure vertical coordinate up to 1 Pa and a sponge layer in the uppermost level. ECHAM6 does not have a single column set-up, so `RCE_large` is initialized from the mean profile on the last day of a simulation with cloud-radiative effects switched off that was run for 30 days (an un-aggregated simulation). The physics packages are PSrad/RRTMG (Iacono et al., 2008) for radiation, a one-moment bulk microphysics scheme (Lohmann & Roeckner, 1996), an eddy-diffusivity approach for boundary layer and sub-grid scale turbulence (Brinkop & Roeckner, 1995), the Nordeng scheme for convection (Nordeng, 1994), and the Sundqvist scheme for stratiform clouds (Sundqvist et al., 1989).

S1.3.5. GEOS-GCM

GEOS 5.21 (Molod et al., 2015; Arnold & Putman, 2018) employs a finite volume dynamical core (Lin, 2004) on a spherical, cubed sphere grid with a time step of 450 s for moist physics. `RCE_small` employs a single column configuration corresponding to a grid spacing of 100 km and 72 hybrid levels. `RCE_large` has 90 x 540 grid points with horizontal grid spacing of ∼ 100 km and 72 hybrid levels. The physics packages are RRTMG for longwave radiation and Chou (1990, 1992) for shortwave radiation, the Bacmeister, Suarez, and Robertson (2006) single-moment microphysics scheme, Lock et al. (2000) and Louis, Tiedtke, and Geleyn (1982) for boundary layer, and the relaxed Arakawa-Schubert convection scheme (Moorthi & Suarez, 1992).

S1.3.6. ICON-GCM

ICON-A is the atmosphere component of the ICON Earth System Model (Giorgetta et al., 2018) which employs a finite volume dynamical core on an icosahedral grid with a time step of 4 minutes. `RCE_large` has 20,480 grid points with a horizontal grid spacing of 158 km and 47 vertical levels, with the top level at 32.9 km and a sponge layer above 25 km. The physics packages are PSRAD (Pincus & Stevens, 2013) for radiation, a cloud microphysical scheme, the Tiedtke mass flux scheme for convection (Tiedtke, 1989) including updates by Nordeng (Nordeng, 1994), and Mauritsen et al. (2007) for vertical diffusion. Because of a bug in the configuration, no cloud microphysics were calculated above a height of $\sim$ 16 km.

S1.3.7. IPSL-CM6

IPSL-CM6A-LR (Hourdin & coauthors, 2020) employs a finite difference dynamical core on a longitude-latitude grid with a time step of 120 s. `RCE_large` has 144 x 142 horizontal grid points and equal area grid spacing with 79 sigma-hybrid vertical levels. Output from the `RCE_small` single column simulation is not available. The physics packages are RRTM (Mlawer et al., 1997) for longwave radiation and Fouquart and Bonnel (1980) and Morcrette (1991) for shortwave radiation, Le Treut and Li (1991) for microphysics, a thermal plume model for shallow convection (Hourdin et al., 2002, 2019), a deep convection scheme based on the Emanuel mass flux scheme (Emanuel, 1991; Grandpeix et al., 2004) with ALE-triggering and ALP closure (Rio et al., 2013), and Yamada (1983) for sub-grid scale turbulence. Several statistical cloud schemes are used in the model - one for shallow clouds (Jam et al., 2013; Hourdin et al., 2019) coupled to the thermal plume model, one for deep convective clouds (Bony & Emanuel, 2001) coupled to the Emanuel convection scheme, and one for stratiform clouds (Madeleine et al., 2020).

S1.3.8. SAM0-UNICON

The Seoul National University Atmosphere Model Version 0 (SAM0-UNICON; Park et al., 2019) is a CAM5-based model that is configured with a spectral element dynamical core on a cubed-sphere mesh, a dynamics time step of 360 s, and a physics time step of 1800 s. The horizontal grid resolution is ne30 which corresponds to a grid spacing of 111 km at the equator. There are 30 hybrid sigma-pressure vertical levels with the midpoint height of the lowest model layer at the sigma pressure level of 0.9926 and the highest model interface at 0.00225 with approximately 1200 m vertical spacing. The physics packages are RRTMG (Iacono et al., 2008; Mitchell, 2002) for radiation, the Morrison and Gettelman (2008) double-moment microphysics scheme, the UW moist turbulence scheme for boundary layer (Bretherton & Park, 2009), UNICON for convection (Park, 2014). Note that SAM0-UNICON differs from CAM5 in its shallow and deep convection scheme and treatment of convective detrainment process in cloud macrophysics scheme (Park et al., 2017).

S1.3.9. SP-CAM, SPX-CAM

The Super-parameterized Community Atmosphere Model (SP-CAM) (Randall et al., 2016) employs a finite volume dynamical core with a spherical grid and a time step of 1800 s. `RCE_large` has 288 x 192 horizontal grid points, corresponding to 1 degree grid spacing, and 26 hybrid pressure levels with a sponge layer at the model top. The radiation scheme is CAM-RT; all other physics parameterizations are represented via super-parameterization with an embedded cloud-resolving model (SAM) (Khairoutdinov & Randall, 2003). There

are 32 CRM columns in each GCM grid cell with 4 km grid spacing and a CRM time step of 20 s. The CRM uses the bottom 24 CAM levels. The microphysics scheme is the SAM single-moment microphysics. The Multi-instance Super-parameterized Community Atmosphere Model (SPX-CAM; Phillips, 2018) differs from SP-CAM in that the surface fluxes are computed separately in each CRM grid column and are averaged back to the GCM.

S1.3.10. UKMO-GA7.1

The Met Office Unified Model Global Atmosphere (GA7.1, CMIP6 model version) (Walters et al., 2019) employs a spherical grid with a 20 minute time step. `RCE_small` employs a single column configuration with 85 vertical levels while `RCE_large` has 192 x 144 horizontal grid points at N96 resolution corresponding to 130 km and 85 vertical levels. The vertical levels are height levels; most variables provided for RCEMIP are on "rho levels" but several are on "theta levels". The physics packages are detailed in Walters et al. (2019).

S1.3.11. WRF-GCM

The Weather Research and Forecasting model v3.5.1 (Skamarock et al., 2008), the same as is used for WRF-COL-CRM, employs the Advanced Research WRF (ARW) dynamical core on a Cartesian grid. The `RCE_large` configuration is the same as for WRF-COL-CRM but has coarser horizontal resolution, fewer vertical levels, and employs convective parameterizations. The domain has 120 x 8 x 48 grid points, with a horizontal grid spacing of 50 km and a time step of 75 seconds. The vertical levels are eta levels (with hydrostatic pressure) with a model top of approximately 33 km and a sponge layer in the top 5 km. The physics packages used are RRTMG (Iacono et al., 2008) for shortwave and longwave radiation, the Morrison et al. (2009) double-moment microphysics scheme, the YSU boundary layer scheme (Noh et al., 2003; Hong et al., 2006), and a horizontal Smagorinsky first-order closure for sub-grid scale turbulence. Six different schemes are used: cps0 (no convective scheme), cps1 (Kain-Fritsch), cps2 (Betts-Miller-Janjic), cps3 (Grell-Freitas), cps4 (Simplified Arakawa Schubert), and cps6 (Tiedtke).

Text S2. Sensitivity of I_{org} Calculation

The calculation of I_{org} is sensitive to numerous details, some of which may not be readily apparent to the casual user. We present our approach to calculating I_{org} to reveal these nuances and so that our results may be reproduced. We discuss three ways to calculate the cumulative distribution function and the sensitivity to the choice in number of connections used in identification of convective entities. Most differences in the calculation approach are small, but they can quickly accumulate for some models.

In addition to which variable and what threshold to use to identify convective pixels, as discussed in the main text's Appendix C, there are four primary considerations when setting up the I_{org} calculation:

1. Periodic boundary conditions should be taken into account, as they allow for convective entities to span the boundaries and for those near the boundaries to consider nearest neighbors near the opposing boundary.
2. For global simulations, limiting the calculation to the tropical band where the curvature of Earth has very little impact on the calculation of distances between entities and the assumption of a cartesian grid can be reasonably applied.

3. The number of connective points by which to classify a group of convective pixels into a convective entitiy
4. Approach to calculating the cumulative density function (CDF, discussed further in **S2.1**.)

The RCEMIP simulations employ doubly periodic boundary conditions for cloud-resolving models (CRMs) and the global simulations are of course cyclic in the zonal direction. These boundary conditions are taken into account when identifying convective entities and their nearest neighbors. We limit the data in the I_{org} calculation for global models to a tropical band of 30°S-30°N and thus must consider the band to be zonally re-entrant. However, the tropical band is not periodic in the meridional direction. The use of a tropical band, rather than the full global domain, is valid as long as the convective entities are significantly smaller than the tropical band, and as long as the aggregation is not so extreme that all convective activity occurs outside of the tropical band. This happens transiently prior to day 80 in the `RCE_large305` simulation in NICAM, so we only consider I_{org} values after day 80 in this simulation.

For the calculation of I_{org} in CRMs, we identify connected convective pixels as convective entities using four-point connectivity (considering adjacent pixels). We tested the sensitivity of the I_{org} calculation to instead using eight-point connectivity (considering adjacent and diagonal pixels) and found it to be relatively insensitive to this choice. Four-point connectivity is also used for the GCM simulations, but given the parameterization of convection and coarse grid spacing, one individual convective pixel may be considered its own convective entity. Therefore, we also present results in which each convective pixel is considered to be independent (that is, using zero connectivity to identify convective entities). It is not clear which approach is more accurate. In the extreme limit of only a few clusters, the nearest neighbor distances between convective entities defined by four-point connectivity would be large and I_{org} may be artificially low. With zero connectivity, on the other hand, I_{org} is dominated by the small scales (i.e., how close convective pixels are to each other within a broader convective area) and may be artificially large. The actual differences depend on the structure of convection in a given model; I_{org} is larger with zero connectivity in some models but smaller in others (Figure S22). While the absolute value of I_{org} is affected by the choice of connectivity, the dependence on SST and the inter-model spread is not (Figure S23).

There are several approaches to the calculation of empirical CDF, discussed below. This paper uses *Method II*. The differences in the value of I_{org} using the three different methods range across models from 0.001 to 0.081 with an average difference of 0.015 ($\sim 2.5\%$ error) when I_{org}'s calculated from Method II and III were considered. All ECHAM6-GCM and WRF-GCM simulations and the warmest CAM5-GCM and CAM6-GCM simulations stand out as the models in which the ECDF method has the strongest impact on the I_{org} calculation. This may be because ECHAM has the coarsest resolution of all GCMs and WRF-GCM has few grid points. The global cloud-resolving model (GCRM) simulations are the least impacted by the choice of ECDF method. Interestingly, these and other (relatively) higher resolution GCMs also have the least difference (and sometimes no difference) between them from the use of different nearest neighbor image processing packages (e.g., Pedregosa et al., 2011; Virtanen et al., 2020).

We note one additional possible source of error in the I_{org} calculation: the nearest neighbor distances are calculated using the gridded indices of the centroids of the convec-

tive entities. This calculation assumes a cartesian grid in which there is equal distance between indices in both horizontal directions. However, this is not always the case in the global simulations. For example, IPSL-CM6, the model with the coarsest resolution, has a grid spacing of 2.5° in the zonal direction and ∼0.8° in the meridional direction (for the latitude range we are considering) which is a difference in distance by a factor of three. However, when the data are interpolated onto a regular 1°x1°, the I_{org} calculation differs by less than 1%, which is as small a difference as that which results from other choices. Therefore, we simply note it as a caveat.

These nuances of the I_{org} calcluation are important to consider if it is desired to reproduce identical results. The sensitivity of the I_{org} calculation in the GCMs, especially to choices as seemingly arbitrary as which ECDF method or which image processing package to use, suggest that I_{org} is perhaps not a metric best suited for use in global models with coarse grids, at least when the absolute value of I_{org} is of interest. The assumption that $I_{org} = 0.5$ represents un-aggregated convection may not be valid in such models.

S2.1. ECDF Theory

The empirical cumulative distribution function, ECDF, is described by the following equation (van der Vaart, 1998):

$$F_n = \frac{\mathrm{N} \leq \mathrm{t}}{n} = \frac{1}{n}\sum_{i=1}^{n} 1_{x_i \leq t} \tag{1}$$

where N is the number of elements less or equal to t, n is the total number of elements, $1_{x_i \leq t}$ is the indicator of event $x_i \leq t$, and the data, x, are independent and identically distributed. The methods described below will use the following list of numbers as example input and an example using a few models will follow the descriptions of the methods: $x_{input} = [0, 0, 1, 2, 2, 3]$. Note each method sorts its input data from lowest to highest, but the example data, x_{input}, is already sorted for simplicity. y_{output} is a vector of values of the empirical CDF evaluated at x_{output}, where this can be determined from x_{input} (a vector of input data values) in several ways different ways (detailed below). The results of the three methods applied to example data are compared in Table S1.

Method I:

Method I is based on the Python package *statsmodels* (Seabold et al., 2010), following `https://www.statsmodels.org/dev/generated/statsmodels.distributions.empirical_distribution.ECDF.html#statsmodels.distributions.empirical_distribution.ECDF`. x_{output} comes from the sorted x_{input} data and $y_{output} = \left[\frac{1}{n}, \frac{2}{n}, ..., \frac{n}{n}\right]$ where n is the number of elements in x_{input}. For our example x_{input}, $x_{output} = [0, 0, 1, 2, 2, 3]$ and $y_{output} = \left[\frac{1}{6}, \frac{2}{6}, \frac{3}{6}, \frac{4}{6}, \frac{5}{6}, \frac{6}{6}\right]$.

Method II:

Method II is based on the R ECDF function (R Core Team, 2017), following `https://www.codementor.io/@kripanshubharga/calculate-ecdf-in-python-gycltzxi3`.

$$x_{output} = [\min(x_{input}), ..., \max(x_{input})] \tag{2}$$

with spacing between elements equal to

$$\frac{\max(x_{\text{input}}) - \min(x_{\text{input}})}{n-1} \tag{3}$$

and y_{output} follows the following formula:

$$y_{output_i} = \frac{\sum x_{input} \leq x_{output_i}}{n} \tag{4}$$

which counts the number of elements of x_{input} that are less than or equal to a particular element of the new x_{output}. The results for our example are as follows:

$$x_{output} = [0.0, 0.6, 1.2, 1.8, 2.4, 3.0] \tag{5}$$

$$y_{output} = \left[\frac{2}{6}, \frac{2}{6}, \frac{3}{6}, \frac{3}{6}, \frac{5}{6}, \frac{6}{6}\right] \tag{6}$$

Method III:

Method III is based on the MATLAB (MATLAB, 2019) ECDF function (Cox & Oakes, 1984; Lawless, 2002; Kleinbaum & Klein, 2005) which uses the Kaplan and Meier (1958) estimate of the CDF.

This method uses a temporary x list containing only unique elements of x_{input} and then calculates a temporary y list containing the total amount of elements in x_{input} that are less than or equal to an element in $x_{\text{temporary}}$

$$x_{temporary} = \text{unique}(x_{input}) \tag{7}$$

$$y_{temporary_i} = \frac{\sum x_{input} \leq x_{temporary_i}}{n} \tag{8}$$

As a final step, a copy of the first element of $x_{temporary}$ is added to the beginning of the $x_{temporary}$ list and a zero is added to the beginning of the $y_{temporary}$ list. For our example:

$$x_{temporary} = [0, 1, 2, 3] \tag{9}$$

$$y_{temporary} = \left[\frac{2}{6}, \frac{3}{6}, \frac{5}{6}, \frac{6}{6}\right] \tag{10}$$

$$x_{output} = [0, 0, 1, 2, 3] \tag{11}$$

$$y_{output} = \left[\frac{0}{6}, \frac{2}{6}, \frac{3}{6}, \frac{5}{6}, \frac{6}{6}\right] \tag{12}$$

Movie S1. Animation of hourly-averaged outgoing longwave radiation (OLR; W m^2) and precipitable water (PW; kg m^{-2}) from day 65 to day 95 in `RCE_large` simulations for CAM5-GCM. Note that the colorbar for PW is different in the 305K simulation.
Movie S2. As in Movie S1 but for CAM6-GCM.
Movie S3. As in Movie S1 but for CM1.
Movie S4. As in Movie S1 but for CNRM-CM6-1.
Movie S5. As in Movie S1 but for DAM.
Movie S6. As in Movie S1 but for ECHAM6-GCM.
Movie S7. As in Movie S1 but for daily-averaged PW only in FV3.
Movie S8. As in Movie S1 but for GEOS-GCM.
Movie S9. As in Movie S1 but for ICON-GCM.
Movie S10. As in Movie S1 but for ICON-LEM.
Movie S11. As in Movie S1 but for ICON-NWP.
Movie S12. As in Movie S1 but for daily-averaged IPSL-CM6.
Movie S13. As in Movie S1 but for MESONH.
Movie S14. As in Movie S1 but for MPAS.
Movie S15. As in Movie S1 but for NICAM.
Movie S16. As in Movie S1 but for SAM0-UNICON.
Movie S17. As in Movie S1 but for SAM-CRM.
Movie S18. As in Movie S1 but for SAM-GCRM.
Movie S19. As in Movie S1 but for SCALE.
Movie S20. As in Movie S1 but for SP-CAM.
Movie S21. As in Movie S1 but for SPX-CAM.
Movie S22. As in Movie S1 but for UCLA-CRM.
Movie S23. As in Movie S1 but for UKMO-GA7.1.
Movie S24. As in Movie S1 but for UKMO-CASIM.
Movie S25. As in Movie S1 but for UKMO-RA1-T.
Movie S26. As in Movie S1 but for UKMO-RA1-T-nocloud.
Movie S27. As in Movie S1 but for WRF-COL-CRM.
Movie S28. As in Movie S1 but for WRF-CRM.
Movie S29. As in Movie S1 but for WRF-GCM-cps0.
Movie S30. As in Movie S1 but for WRF-GCM-cps1.
Movie S31. As in Movie S1 but for WRF-GCM-cps2.
Movie S32. As in Movie S1 but for WRF-GCM-cps3.
Movie S33. As in Movie S1 but for WRF-GCM-cps4.
Movie S34. As in Movie S1 but for WRF-GCM-cps6.
Movie S35. Animation of hourly-averaged outgoing longwave radiation (OLR; W m^2) and precipitable water (PW; kg m^{-2}) from day 65 to day 95 in `RCE_small` simulations for CM1.
Movie S36. As in Movie S35 but for DALES.
Movie S37. As in Movie S35 but for DALES-damping.
Movie S38. As in Movie S35 but for DAM. OLR data after day 85.5 in `RCE_small295` is missing.
Movie S39. As in Movie S35 but for ICON-LEM.
Movie S40. As in Movie S35 but for ICON-NWP.
Movie S41. As in Movie S35 but for MESONH.

Movie S42. As in Movie S35 but for MicroHH.
Movie S43. As in Movie S35 but for MPAS.
Movie S44. As in Movie S35 but for SAM-CRM.
Movie S45. As in Movie S35 but for SCALE.
Movie S46. As in Movie S35 but for UCLA-CRM.
Movie S47. As in Movie S35 but for UKMO-CASIM.
Movie S48. As in Movie S35 but for UKMO-RA1-T.
Movie S49. As in Movie S35 but for UKMO-RA1-T-hrad.
Movie S50. As in Movie S35 but for UKMO-RA1-T-nocloud.
Movie S51. As in Movie S35 but for WRF-COL-CRM.
Movie S52. As in Movie S35 but for WRF-CRM.
Movie S53. Animation of hourly-averaged outgoing longwave radiation (OLR; W m^2) and precipitable water (PW; kg m^{-2}) from day 65 to day 95 in `RCE_small_vert` simulations for CM1.
Movie S54. As in Movie S53 but for DALES.
Movie S55. As in Movie S53 but for DALES-damping.
Movie S56. As in Movie S53 but for ICON-LEM.
Movie S57. As in Movie S53 but for MESONH.
Movie S58. As in Movie S53 but for MicroHH.
Movie S59. As in Movie S53 but for SAM-CRM.
Movie S60. Animation of hourly-averaged outgoing longwave radiation (OLR; W m^2) and precipitable water (PW; kg m^{-2}) from day 20 to day 50 in `RCE_small_les` simulations for CM1.
Movie S61. As in Movie S60 but for DALES.
Movie S62. As in Movie S60 but for ICON-LEM.
Movie S63. As in Movie S60 but for MESONH.
Movie S64. As in Movie S60 but for MicroHH.
Movie S65. As in Movie S60 but for SAM-CRM.

References

Arnold, N. P., & Putman, W. M. (2018). Nonrotating convective self-aggregation in a limited area AGCM. *J. Adv. Model. Earth Syst.*, *10*(4), 1029–1046. doi: 10.1002/2017ms001218

Bacmeister, J. T., Suarez, M. J., & Robertson, F. R. (2006). Rain reevaporation, boundary layer-convection interactions, and pacific rainfall patterns in an AGCM. *J. Atmos. Sci.*, *63*(12), 3383–3403. doi: 10.1175/jas3791.1

Beljaars, A., & Holtslag, A. (1991). Flux parameterization over land surfaces for atmospheric models. *J. Appl. Meteor.*, *30*, 327-341.

Bogenschutz, P. A., Gettelman, A., Morrison, H., Larson, V. E., Craig, C., & Schanen, D. P. (2013). Higher-order turbulence closure and its impact on climate simulations in the community atmosphere model. *J. Climate*, *26*(23), 9655–9676. doi: 10.1175/jcli-d-13-00075.1

Bony, S., & Emanuel, K. A. (2001). A parameterization of the cloudiness associated with cumulus convection; evaluation using TOGA COARE data. *J. Atmos. Sci.*, *58*, 3158-3183.

Bougeault, P. (1981). Modeling the trade-wind cumulus boundary layer. Part I: Testing the ensemble cloud relations against numerical data. *J. Atmos. Sci.*, *38*(11), 2414-2428.

Bougeault, P., & Lacarrere, P. (1989). Parameterization of orograophy-induced turbulence in a Mesobeta–Scale model. *Mon. Wea. Rev.*, *117*, 1872-1890.

Bretherton, C. S., & Park, S. (2009). A new moist turbulence parameterization in the Community Atmosphere Model. *Journal of Climate*, *22*(12), 3422–3448. doi: 10.10.1175/2008JCLI2556.1

Brinkop, S., & Roeckner, E. (1995). Sensitivity of a general circulation model to parameterizations of cloud-turbulence interactions in the atmospheric boundary layer. *Tellus A*, *47*, 197-220.

Bryan, G. H., & Morrison, H. (2012). Sensitivity of a simulated squall line to horizontal resolution and parameterization of microphysics. *Mon. Wea. Review*. doi: 10.1175/MWR-D-11-00046.1

Bryan, G. H., & Rotunno, R. (2009). The maximum intensity of tropical cyclones in axisymmetric numerical model simulations. *Mon. Wea. Rev.*, *137*, 1770-1789. doi:

10.1175/2008MWR2709.1

Chou, M. D. (1990). Parameterizations for the absorption of solar radiation by O2 and CO2 with application to climate studies. *J. Climate*, *3*(2), 209–217.

Chou, M. D. (1992). A solar radiation model for use in climate studies. *J. Atmos. Sci.*, *49*(9), 762–772.

Chou, M. D., & Suarez, M. J. (1999). *A solar radiation parameterization for atmospheric studies* (Tech. Rep. Nos. 104606, 15, 40 pp.) NASA Tech. Memo.

Chou, M. D., Suarez, M. J., Liang, X. Z., & Yan, M. M. H. (2001). *A thermal infrared radiation parameterization for atmospheric studies* (Tech. Rep. Nos. 104606, 19, 68 pp.) NASA Tech. Memo.

Cox, D., & Oakes, D. (1984). *Analysis of survival data.* London: Chapman and Hall.

Cuxart, J., Bougeault, P., & Redelsperger, J.-L. (2000). A turbulence scheme allowing for mesoscale and large-eddy simulations. *Q. J. R. Meteorol. Soc.*, *126*, 1-30.

Deardorff, J. (1980). Stratocumulus-capped mixed layers derived from a three-dimensional model. *Boundary-Layer Meteorol.*, *18*, 495-527.

Dipankar, A., Stevens, B., Heinze, R., Moseley, C., Zängl, G., Giorgetta, M., & Brdar, S. (2015). Large eddy simulation using the general circulation model ICON. *J. Adv. Model. Earth Sys.*, *7*, 963-986. doi: 10.1002/2015MS000431

Dudhia, J. (1996). A multi-layer soil temperature model for MM5. *The Sixth PSU/NCAR Mesoscale Model Users' Workshop.*

Edwards, J., & Slingo, A. (1996). Studies with a flexible new radiation code. I: Choosing a configuration for a large-scale model. *Q. J. R. Meteorol. Soc.*, *122*, 689-719. doi: 10.1002/qj.49712253107

Emanuel, K. (1991). A scheme for representing cumulus convection in large-scale models. *J. Atmos. Sci.*, *48*, 2313-2329.

Fouquart, Y., & Bonnel, B. (1980). Computations of solar heating of the Earth's atmosphere - a new parmeterization. *Beitrage zur Physik der Atmosphere*, *53*, 35-62.

Gettelman, A., & Morrison, H. (2015). Advanced two-moment bulk microphysics for global models. part i: Off-line tests and comparison with other schemes. *J. Climate*, *28*(3), 1268–1287. doi: 10.1175/jcli-d-14-00102.1

Giorgetta, M. A., Brokopf, R., Crueger, T., Esch, M., Fiedler, S., Helmert, J., ... Stevens, B. (2018). ICON-A, the atmosphere component of the ICON earth system model: I. model description. *J. Adv. Model. Earth Syst.*, *10*(7), 1613–1637. doi: 10.1029/2017ms001242

Grabowski, W. W. (1998). Toward cloud resolving modeling of large-scale tropical circulations: A simple cloud microphysics parameterization. *J. Atmos. Sci.*, *55*(21), 3283–3298. doi: 10.1175/1520-0469(1998)055⟨3283:tcrmol⟩2.0.co;2

Grandpeix, J. Y., Phillips, V., & Tailleux, R. (2004). Improved mixing representation in emanuel's convection scheme. *Q. J. R. Meteorol. Soc.*, *130*, 3207-3222.

Guérémy, J. F. (2011). A continuous buoyancy based convection scheme: one- and three-dimensional validation. *Tellus A*, *63*(4), 687–706. doi: 10.1111/j.1600-0870.2011.00521.x

Harris, L. M., & Lin, S.-J. (2013). A two-way nested global-regional dynamical core on the cubed-sphere grid. *Mon. Wea. Rev.*, *141*(1), 283–306. doi: 10.1175/mwr-d-11-00201.1

Heus, T., van Heerwaarden, C. C., Jonker, H. J. J., Pier Siebesma, A., Axelsen, S.,

van den Dries, K., ... Vilà-Guerau de Arellano, J. (2010). Formulation of the Dutch Atmospheric Large-Eddy Simulation (DALES) and overview of its applications. *Geoscientific Model Development*, *3*(2), 415–444. doi: 10.5194/gmd-3-415-2010

Hill, A. A., Shipway, B. J., & Boutle, I. A. (2015). How sensitive are aerosol-precipitation interactions to the warm rain representation? *J. Adv. Model. Earth Syst.*, *7*, 987–1004. doi: 10.1002/2014ms000422

Hohenegger, C., & Stevens, B. (2016). Coupled radiative convective equilibrium simulationswith explicit and parameterized convection. *J. Adv. Model. Earth Sys.*, *8*. doi: 10.1002/2016MS000666

Hong, S.-Y., Dudhia, J., & Chen, S.-H. (2004). A Revised Approach to Ice Microphysical Processes for the Bulk Parameterization of Clouds and Precipitation. *Mon. Wea. Rev.*, *132*, 103–120. doi: 10.1175/1520-0493(2004)132⟨0103:ARATIM⟩2.0.CO;2

Hong, S.-Y., Noh, Y., & Dudhia, J. (2006). A new vertical diffusion package with an explicit treatment of entrainment processes. *Mon. Wea. Rev.*, *134*, 2318-2341. doi: 10.1175/Mwr3199.1

Hourdin, F., & coauthors. (2020). LMDZ6A: The atmospheric component of the ISPL climate model with improved and better tuned physics. *J. Adv. Model. Earth Syst.*, *12*, e2019MS001892. doi: 10.1029/2019MS001892

Hourdin, F., Couvreux, F., & Menut, L. (2002). Parameterization of the dry convective boundary layer based on a mass flux representation of thermals. *J. Atmos. Sci.*, *59*(6), 1105–1123. doi: 10.1175/1520-0469(2002)059⟨1105:potdcb⟩2.0.co;2

Hourdin, F., Jam, A., Rio, C., Couvreux, F., Sandu, I., Lefebvre, M.-P., ... Idelkadi, A. (2019). Unified parameterization of convective boundary layer transport and clouds

with the thermal plume model. *J. Adv. Model. Earth Syst.*, *11*(9), 2910–2933. doi: 10.1029/2019ms001666

Iacono, M. J., Delamere, J. S., Mlawer, E. J., Shephard, M. W., Clough, S. A., & Collins, W. D. (2008). Radiative forcing by long-lived greenhousegases: calculations with the AER radiative transfer models. *J. Geophys. Res.*, *113*, D13103. doi: 10.1029/2008JD009944

Jam, A., Hourdin, F., Rio, C., & Couvreux, F. (2013). Resolved versus parametrized boundary-layer plumes. part iii: Derivation of a statistical scheme for cumulus clouds. *Boundary-Layer Meteorology*, *147*(3), 421–441. doi: 10.1007/s10546-012-9789-3

Jimenez, P., Dudhia, J., Gonzalez-Rouco, J., Navarro, J., Montavez, J., & Garcia-Bustamante, E. (2012). A revised scheme for the wrf surface layer formulation. *Mon. Wea. Rev.*, *140*, 898-918. doi: 10.1175/MWR-D-11-00056.1

Kaplan, E. L., & Meier, P. (1958). Nonparametric estimation from incomplete observations. *J. Amer. Statist. Assoc.*, *53*(282), 457-481. doi: 10.2307/2281868 .JSTOR2281868

Khairoutdinov, M. F., & Randall, D. (2003). Cloud resolving modeling of the ARM Summer 1997 IOP: Model formulation, results, uncertainties, and sensitivities. *J. Atmos. Sci.*, *60*, 607-625.

Khairoutdinov, M. F., & Randall, D. (2006). High-resolution simulation of shallow-to-deep convection transition over land. *J. Atmos. Sci.*, *63*(12), 3421–3436. doi: 10.1175/jas3810.1

Kleinbaum, D., & Klein, M. (2005). Statistics for biology and health, 2nd edition. In (chap. Suvival Analysis). Springer.

Lac, C., Chaboureau, J.-P., Masson, V., Pinty, J.-P., Tulet, P., Escobar, J., ... Wautelet, P. (2018). Overview of the Meso-NH model version 5.4 and its applications. *Geosci. Model Dev.*, *11*, 1929-1969. doi: 10.5194/gmd-11-1929-2018

Lauritzen, P. H., Nair, R. D., Herrington, A., Callaghan, P., Goldhaber, S., Dennis, J., ... others (2018). Ncar release of cam-se in cesm2. 0: A reformulation of the spectral element dynamical core in dry-mass vertical coordinates with comprehensive treatment of condensates and energy. *Journal of Advances in Modeling Earth Systems*, *10*(7), 1537–1570.

Lawless, J. (2002). *Statistical models and methods for lifetime data.* Hoboken, NJ: Wiley-Interscience.

Le Treut, H., & Li, Z.-X. (1991). Sensitivity of an atmospheric general circulation model to prescribed sst changes: feedback effects associated with the simulation of cloud optical properties. *Climate Dynamics*, *5*(3), 175–187. doi: 10.1007/BF00251808

Lim, K. S., & Hong, S. (2010). Development of an effective double-moment cloud microphysics scheme with prognostic cloud condensation nuclei (CCN) for weather and climate models. *Mon. Wea. Rev.*, *138*, 1587-1612. doi: 10.1175/2009MWR2968.1

Lin, S.-J. (2004). A vertically lagrangian finite-volume dynamical core for global models. *Mon. Wea. Rev.*, *132*, 2293–2307.

Lock, A., Brown, A., Bush, M., Martin, G., & Smith, R. (2000). A new boundary layer mixing scheme. Part I: Scheme description and single-column model tests. *Mon. Wea. Rev.*, *128*, 3187-3199.

Lohmann, U., & Roeckner, E. (1996). Design and performance of a new cloud micro-

physics scheme developed for the ECHAM general circulation model. *Clim. Dyn.*, *12*, 557-572.

Lopez, P. (2002). Implementation and validation of a new prognostic large-scale cloud and precipitation scheme for climate and data-assimilation purposes. *Q.J.R. Meteorol. Soc.*, *128*(579), 229–257. doi: 10.1256/00359000260498879

Louis, J.-F., Tiedtke, M., & Geleyn, J.-F. (1982). A short history of the PBL parameterization of ECMWF. In *ECMWF workshop on planetary boundary layer parameterization* (p. 59-79). Reading, UK: ECWMF.

Madeleine, J.-B., Hourdin, F., Grandpeix, J.-Y., Rio, C., Dufresne, J.-L., Konsta, D., ... Bonazzola, M. (2020). Improved representation of clouds in the LMDZ6A global climate model. *J. Adv. Model. Earth Syst.*, submitted.

Manners, J., Edwards, J., Hill, P., & Thelen, J.-C. (2015). *SOCRATES Technical Guide: Suite of Community RAdiative Transfer codes based on Edwards and Slingo* (Tech. Rep.). Met Office. Retrieved from `http://homepages.see.leeds.ac.uk/~lecsjed/winscpuse/socrates_techguide.pdf`

Martin, Z., Wang, S., Nie, J., & Sobel, A. (2019). The impact of the QBO on MJO convection in cloud-resolving simulations. *J. Atmos. Sci.*, *76*, 669-688. doi: 10.1175/JAS-D-18-0179.1

MATLAB. (2019). *9.7.0.1190202 (r2019b).* Natick, MA: The MathWorks Inc.

Mauritsen, T., Svensson, G., Zilitinkevich, S. S., Esau, I., Enger, L., & Grisogono, B. (2007). A total turbulent energy closure model for neutrally and stably stratified atmospheric boundary layers. *J. Atmos. Sci.*, *64*(11), 4113–4126. doi: 10.1175/2007jas2294.1

Mitchell, D. L. (2002). Effective diameter in radiation transfer: General definition, applications, and limitations. *J. Atmos. Sci.*, *59*(15), 2330–2346. doi: 10.1175/1520-0469(2002)059⟨2330:edirtg⟩2.0.co;2

Mlawer, E. J., Taubman, S. J., Brown, P. D., Iacono, M. J., & Clough, S. A. (1997). Radiative transfer for inhomogeneous atmospheres: RRTM, a validated correlated-k model for the longwave. *J. Geophys. Res.*, *102*, 16663-16682.

Molod, A., Takacs, L., Suarez, M., & Bacmeister, J. (2015). Development of the GEOS-5 atmospheric general circulation model: Evolution from MERRA to MERRA2. *Geoscientific Model Development*, *8*(5), 1339–1356. doi: 10.5194/gmd-8-1339-2015

Moon, I.-J., Ginis, I., Hara, T., & Thomas, B. (2007). A physics-based parameterization of air sea momentum flux at high wind speeds and its impact on hurricane intensity predictions. *Mon. Wea. Rev.*, *135*, 2869-2878.

Moorthi, S., & Suarez, M. J. (1992). Relaxed arakawa-schubert. a parameterization of moist convection for general circulation models. *Mon. Wea. Rev.*, *120*(6), 978–1002.

Morcrette, J.-J. (1991). Radiation and cloud radiative properties in the European Centre for Medium Range Weather Forecasts forecasting system. *J. Geophys. Res.*, *96*(D5), 9121–9132. doi: 10.1029/89jd01597

Morrison, H., & Gettelman, A. (2008). A new two-moment bulk stratiform cloud microphysics scheme in the community atmosphere model, version 3 (cam3). part i: Description and numerical tests. *J. Climate*, *21*(15), 3642–3659. doi: 10.1175/2008jcli2105.1

Morrison, H., McCoy, R. B., Klein, S. A., Xie, S., Luo, Y., Avramov, A., … Zhang, G. (2009). Intercomparison of model simulations of mixed-phase clouds observed

during the ARM Mixed-Phase Arctic Cloud Experiment. II: Multilayer clouds. *Q. J. R. Meteorol. Soc.*, *153*, 1003-1019. doi: doi:10.1002/qj.415

Nakanishi, M., & Niino, H. (2004). An improved Mellor-Yamada level-3 model with condensation physics: Its design and verification. *Bound. Layer Meteor.*, *112*, 1-31.

Neale, R., Chen, C.-C., Gettelman, A., Lauritzen, P., Park, S., WIlliamson, D., ... Taylor, M. (2012). Description of the NCAR Community Atmosphere Model (CAM 5.0). *NCAR Tech. Note TN-486*. Retrieved from http://www.cesm.ucar.edu/models/cesm1.0/cam/docs/description/cam5_desc.pdf

Nishizawa, S., Yashiro, H., Sato, Y., Miyamoto, Y., & Tomita, H. (2015). Influence of grid aspect ratio on planetary boundary layer turbulence in large-eddy simulations. *Geosci. Model Dev.*, *8*, 3393-3419. doi: 10.5194/gmd-8-3393-2015

Noh, Y., Cheon, W., Hong, S., & Raasch, S. (2003). Improvement of the K-profile model for the planetary boundary layer based on large eddy simulation data. *Boundary-Layer Meteorology*, *107*, 401-427.

Nordeng, T. (1994). *Extended versions of the convective parameterization scheme at ECMWF and their impact on the mean and transient activity of the model in the tropics* (Tech. Rep. No. 206). Eur. Cent. for Medium-Range Weather Forecasts, Reading, U. K.

Park, S. (2014). A unified convection scheme (UNICON). Part I: Formulation. *J. Atmos. Sci.*, *71*(11), 3902–3930. doi: 10.1175/jas-d-13-0233.1

Park, S., Baek, E.-H., Kim, B.-M., & Kim, S.-J. (2017). Impact of detrained cumulus on climate simulated by the Community Atmosphere Model version 5 with a unified convection scheme. *J. Adv. Model. Earth Syst.*, *9*(2), 1399–1411. doi: 10.1002/

2016ms000877

Park, S., & Bretherton, C. S. (2009). The University of Washington shallow convection and moist turbulence schemes and their impact on climate simulations with the Community Atmosphere Model. *Journal of Climate*, *22*(12), 3449-3469. doi: 10.1175/2008JCLI2557.1

Park, S., Shin, J., Kim, S., Oh, E., & Kim, Y. (2019). Global climate simulated by the Seoul National University Atmosphere Model Version 0 with a Unified Convection Scheme (SAM0-UNICON). *J. Climate*, *32*(10), 2917–2949. doi: 10.1175/jcli-d-18-0796.1

Pedregosa, F., Varoquaux, G., Gramfort, A., Michel, V., Thirion, B., Grisel, O., ... Duchesnay, E. (2011). Scikit-learn: Machine learning in Python. *Journal of Machine Learning Research*, *12*, 2825–2830.

Pergaud, J., Masson, V., Malardel, S., & Couvreux, F. (2009). A parmeterization of dry thermals and shallow cumuli for mesoscale numerical weather prediction. *Boundary-Layer Meteorology*, *132*, 83. doi: 10.1007/s10546-009-9388-0

Phillips, M. (2018). *Multiple scale of surface-atmosphere coupling in an Earth system model* (Unpublished doctoral dissertation). Colorado State University, 111 pp.

Pincus, R., Mlawer, E., & Delamere, J. S. (2019). Balancing accuracy, efficiency, and flexibility in radiation calculations for dynamical models. *J. Adv. Model. Earth Sys.*, *11*. doi: 10.1029/2019MS001621

Pincus, R., & Stevens, B. (2009). Monte Carlo spectral integration: a consistent appr for radiative transfer in large eddy simulations. *J. Adv. Model. Earth Sys.*, *1*, 1. doi: 10.3894/JAMES.2009.1.1.

Pincus, R., & Stevens, B. (2013). Paths to accuracy for radiation parameterizations in atmospheric models. *J. Adv. Model. Earth Syst.*, *5*(2), 225–233. doi: 10.1002/jame.20027

Pinty, J., & Jabouille, P. (1998). A mixed-phase cloud parameterization for use in mesoscale non-hydrostatic model: Simulations of a squall line and of orographic precipitation. *Preprints of the conference on cloud physics, Everett, WA*, *American Meteorological Society, Boston, USA*, 217-220.

Piriou, J.-M., Redelsperger, J.-L., Geleyn, J.-F., Lafore, J.-P., & Guichard, F. (2007). An approach for convective parameterization with memory: Separating microphysics and transport in grid-scale equations. *J. Atmos. Sci.*, *64*(11), 4127–4139. doi: 10.1175/2007jas2144.1

Putman, W., & Lin, S.-J. (2007). Finite-volume transport on various cubed-sphere grids. *J. Comput. Phys.*, *64*, 3377-3405.

R Core Team. (2017). R: A language and environment for statistical computing [Computer software manual]. Vienna, Austria. Retrieved from `https://www.R-project.org/`

Randall, D. A., DeMott, C., Stan, C., Khairoutdinov, M., Benedict, J., McCrary, R., ... Branson, M. (2016). Simulations of the tropical general circulation with a multiscale global model. *Meteorological Monographs*, *56*, 15.1–15.15. doi: 10.1175/amsmonographs-d-15-0016.1

Raschendorfer, M. (2001). The new turbulence parametrization of LM. *COSMO Newsl.*, *1*, 90-98.

Rio, C., Grandpeix, J.-Y., Hourdin, F., Guichard, F., Couvreux, F., Lafore, J.-P., ...

Idelkadi, A. (2013). Control of deep convection by sub-cloud lifting processes: the ALP closure in the LMDZ5B general circulation model. *Climate Dynamics*, *40*(9), 2271–2292. doi: 10.1007/s00382-012-1506-x

Roehrig, R., & coauthors. (2020). The CNRM global atmosphere model ARPEGE-Climate 6.3: description and evaluation. *J. Adv. Model. Earth Syst.*, *12*, e2020MS002075. doi: 10.1029/2020MS002075

Romps, D. M. (2008). The dry-entropy budget of a moist atmosphere. *J. Atmos. Sci.*, *65*, 3779-3799.

Sato, Y., Nishizawa, S., Yashiro, H., Miyamoto, Y., Kajikawa, Y., & Tomita, H. (2015). Impacts of cloud microphysics on trade wind cumulus: which cloud microphysics processes contribute to the diversity in a large eddy simulation? *Prog. Earth Planet. Sci.*, *2*, 23. doi: 10.1186/s40645-015-0053-6

Satoh, M., Stevens, B., Judt, F., Khairoutdinov, M., Lin, S.-J., Putnam, W., & Düben, P. (2019). Global cloud-resolving models. *Current Climate Change Reports*, *5*, 172-184. doi: 10.1007/s40641-019-00131-0

Satoh, M., Tomita, H., Yashiro, H., Miura, H., Kodama, C., Seiki, T., … Kubokawa, H. (2014). The Non-hydrostatic ICosahedral Atmospheric Model: Description and development. *Progress in Earth and Planetary Science*, *1*, 18. doi: 10.1186/s40645-014-0018-1

Seabold, Skipper, & Perktold, J. (2010). statsmodels: Econometric and statistical modeling with python. In *Proceedings of the 9th python in science conference.*

Seifert, A., & Beheng, K. (2006). A two-moment cloud microphysics parameterization for mixed-phase clouds. Part 1: Model description. *Meteorology and Atmospheric*

Physics, *92*, 45-66.

Sekiguchi, M., & Nakajima, T. (2008). A k-distribution-based radiation code and its computational optimization fo an atmospheric general circulation model. *J. Quant. Spectrosc. Radiat. Transfer*, *109*, 2779-2793. doi: 10.1016/j.jqsrt.2008.07.013

Shipway, B., & Hill, A. (2012). Diagnosis of systematic differences between multiple parameterizations of warm rain microphysics using a kinematic framework. *Q. J. R. Meteorol. Soc.*, *138*, 2196-2211. doi: 10.1002/qj.1913

Skamarock, W. C., Klemp, J., Dudhia, J., Gill, D., Barker, D., Duda, M., ... Powers, J. (2008). *A description of the Advanced Research WRF Version 3* (Tech. Rep. Nos. NCAR/TN-475+STR). University Corporation for Atmospheric Research. doi: 10.5065/D68S4MVH

Skamarock, W. C., Klemp, J. B., Duda, M. G., Fowler, L. D., Park, S.-H., & Ringler, T. D. (2012). A multiscale nonhydrostatic atmospheric model using centroidal voronoi tesselations and c-grid staggering. *Mon. Wea. Rev.*, *140*, 3090–3105. doi: 10.1175/mwr-d-11-00215.1

Smagorinksy, J. (1963). General circulation experiments with the primitive equations. *Mon. Wea. Rev.*, *91*, 99-164.

Smith, R. (1990). A scheme for predicting layer clouds and their water contents in a general circulation model. *Q. J. R. Meteorol. Soc.*, *116*, 435-460.

Stevens, B., Giorgetta, M., Esch, M., Mauritsen, T., Crueger, T., Rast, S., ... Roeckner, E. (2013). Atmospheric component of the MPI-M Earth System model: ECHAM6. *J. Adv. Model. Earth Syst.*, *5*(2), 146–172. doi: 10.1002/jame.20015

Stratton, R., Senior, C., Vosper, S., Folwell, S., Boutle, I., Earnshaw, P., ... Wilkinson,

J. M. (2018). A Pan-African convection-permitting regional climate simulation with the Met Office Unified Model: CP4-Africa. *J. Climate*, *31*, 3485-3508.

Sundqvist, H., Berge, E., & Kristjansson, J. E. (1989). Condensation and cloud parameterization studies with a mesoscale numerical weather prediction model. *Mon. Weather Rev.*, *117*, 1641-1657.

Tiedtke, M. (1989). A comprehensive mass flux scheme for cumulus parameterization in large-scale models. *Mon. Wea. Rev.*, *117*(8), 1779–1800.

Tomita, H. (2008). New microphysical schemes with five and six categories by diagnostic generation of cloud ice. *J. Meteor. Soc. Japan*, *86A*, 121-142. doi: 10.2151/jmsj.86A.121

van der Vaart, A. (1998). *Asymptotic statistics* (No. p. 265). Cambridge University Press.

van Heerwaarden, C., van Stratum, B. J. H., Heus, T., Gibbs, J., Fedorovich, E., & Mellado, J. (2017). MicroHH 1.0: a computational fluid dynamics code for direct numerical simulation and large-eddy simulation of atmospheric boundary layer flows. *Geosci. Model Dev.*, *10*, 3145-3165. doi: 10.5194/gmd-10-3145-2017

Virtanen, P., Gommers, R., Oliphant, T. E., Haberland, M., Reddy, T., Cournapeau, D., . . . Contributors, S. . . (2020). SciPy 1.0: Fundamental Algorithms for Scientific Computing in Python. *Nature Methods*, *17*, 261–272. doi: https://doi.org/10.1038/s41592-019-0686-2

Voldoire, A., & coauthors. (2019). Evaluation of CMIP6 DECK expeirments with CNRM-CM6.1. *J. Adv. Model. Earth Syst.*, *11*, 2177-2213. doi: 10.1029/2019MS001683

Walters, D., Baran, A. J., Boutle, I., Brooks, M., Earnshaw, P., Edwards, J., . . . Zer-

roukat, M. (2019). The Met Office Unified Model Global Atmosphere 7.0/7.1 and JULES global land 7.0 configurations. *Geoscientific Model Development*, *12*(5), 1909–1963. doi: 10.5194/gmd-12-1909-2019

Wang, S., Sobel, A. H., Nie, J., & Sobel, A. (2016). Modeling the MJO in a cloud-resolving modelwith parameterized large-scale dynamics: vertical structure, radiation, and horizontal advection of dry air. *J. Adv. Model. Earth Syst.*, *8*, 121-139. doi: 10.1002/2015MS000529

Wicker, L., & Skamarock, W. (2002). Time splitting methods for elastic models using forward time schemes. *Mon. Wea. Rev.*, *130*, 2088-2097.

Wilson, D. K. (2001). An alternative function for the wind and temperature gradients in unstable surface layers. *Boundary-Layer Meteorology*, *99*, 151-158.

Wilson, D. R., & Ballard, S. (1999). A microphysically based precipitation scheme for the UK meteorological office unified models. *Q. J. R. Meteorol. Soc.*, *125*, 1607-1636. doi: 10.1002/qj.49712555707

Wing, A. A., Reed, K. A., Satoh, M., Stevens, B., Bony, S., & Ohno, T. (2018). Radiative-Convective Equilibrium Model Intercomparison Project. *Geosci. Model Dev.*, *11*, 793-813. doi: 10.5194/gmd-11-793-2018

Wood, N., Staniforth, A., White, A., Allen, T., Diamantakis, M., Gross, M., ... Thuburn, J. (2014). An inherently mass-conserving semi-implicit semi-Lagrangian discretization of the deep-atmosphere global non-hydrostatic equations. *Q. J. R. Meteorol. Soc.*, *140*, 1505-1520. doi: 10.1002/qj.2235

Yamada, T. (1983, January). Simulations of nocturnal drainage flows by a q2l turbulence closure model. *J. Atmos. Sci.*, *40*(1), 91–106.

Zängl, G., Reinert, D., Ripodas, P., & Baldauf, M. (2015). The ICON (ICOsahedral Non-hydrostatic) modeling framework of DWD and MPI-M: Description of the non-hydrostatic dynamical core. *Q. J. R. Meteorol. Soc.*, *141*, 563-579.

Zhang, G. J., & McFarlane, N. A. (1995). Sensitivity of climate simulations to the parameterization of cumulus convection in the Canadian Climate Centre general circulation model. *Atmosphere-Ocean*, *33*, 407-446.

Zhou, L., Lin, S.-J., Chen, J.-H., Harris, L. M., Chen, X., & Rees, S. L. (2019). Toward convective-scale prediction within the next generation global prediction system. *Bull. Amer. Meteor. Soc.*, *100*(7), 1225–1243. doi: 10.1175/bams-d-17-0246.1

Table S1. Comparison of ECDF methods for example data.

Method	x_{output}	y_{output}
I	$[0, 0, 1, 2, 2, 3]$	$\left[\frac{1}{6}, \frac{2}{6}, \frac{3}{6}, \frac{4}{6}, \frac{5}{6}, \frac{6}{6}\right]$
II	$[0.0, 0.6, 1.2, 1.8, 2.4, 3.0]$	$\left[\frac{2}{6}, \frac{2}{6}, \frac{3}{6}, \frac{3}{6}, \frac{5}{6}, \frac{6}{6}\right]$
III	$[0, 0, 1, 2, 3]$	$\left[\frac{0}{6}, \frac{2}{6}, \frac{3}{6}, \frac{5}{6}, \frac{6}{6}\right]$

Table S2. `RCE_small` Anvil Cloud Fraction

**Model**	295K	300K	305K
CAM5-GCM	0.709	0.775	0.791
CAM6-GCM	0.994	0.976	0.915
CM1	0.594	0.568	0.5483
CM1-VER	0.481	0.471	0.488
CM1-LES	0.514	0.483	0.442
CNRM-CM6-1	0.929	0.957	0.962
DALES	0.152	0.285	0.033
DALES-VER	0.122	0.270	0.139
DALES-LES	0.259	0.294	0.145
DALES-damping	0.159	0.422	0.030
DALES-damping-VER	0.104	0.080	0.041
DAM	0.245	0.208	0.174
GEOS-GCM	0.296	0.3685	0.4401
ICON-LEM	1.000	0.999	0.989
ICON-LEM-VER	0.998	0.986	0.917
ICON-LEM-LES	0.996	0.986	0.913
ICON-NWP	0.324	0.318	0.324
MESONH	0.166	0.155	0.150
MESONH-VER	0.161	0.152	0.152
MESONH-LES	0.184	0.196	0.194
MicroHH	0.107	0.106	0.110
MicroHH-VER	0.080	0.082	0.082
MicroHH-LES	0.081	0.075	0.053
MPAS	0.164	0.127	0.100
SAM-CRM	0.263	0.241	0.204
SAM-CRM-VER	0.125	0.119	0.112
SAM-CRM-LES	0.249	0.223	0.171
SCALE	0.238	0.248	0.217
UCLA-CRM	0.078	0.082	0.063
UKMO-GA7.1	0.956	0.906	0.880
UKMO-CASIM	0.297	0.305	0.320
UKMO-RA1-T-hrad	1.000	1.000	1.000
UKMO-RA1-T	1.000	1.000	1.000
UKMO-RA1-T-nocloud	1.000	1.000	1.000
WRF-COL-CRM	0.445	0.454	0.442
WRF-CRM	0.247	0.186	0.257
WRF-GCM-cps0	0.819	0.812	0.771
WRF-GCM-cps1	0.871	0.866	0.858
WRF-GCM-cps2	0.802	0.745	0.723
WRF-GCM-cps3	0.944	0.789	0.733
WRF-GCM-cps4	0.950	0.691	0.898
WRF-GCM-cps6	0.965	0.992	0.991
MEAN-ALL	0.502	0.500	0.471
IQR-ALL	0.776	0.654	0.728
RANGE-ALL	0.922	0.925	0.970
MEAN-PARAM.	0.839	0.807	0.815
IQR-PARAM.	0.789	0.733	0.760
RANGE-PARAM.	0.916	0.917	0.961
MEAN-EXPLICIT	0.382	0.391	0.348
IQR-EXPLICIT	0.333	0.278	0.319
RANGE-EXPLICIT	0.922	0.925	0.970

Table S3. RCE_small Anvil Cloud Temperature [K]

**Model**	295K	300K	305K
CAM5-GCM	214.8	216.7	217.7
CAM6-GCM	214.5	214.3	215.0
CM1	207.4	209.9	210.0
CM1-VER	212.7	213.1	212.0
CM1-LES	212.1	215.9	214.9
CNRM-CM6-1	203.1	201.9	202.9
DALES	207.2	217.0	235.4
DALES-VER	221.8	223.2	227.7
DALES-LES	211.7	218.3	220.4
DALES-damping	207.1	216.1	237.1
DALES-damping-VER	204.5	214.6	218.8
DAM	211.2	213.5	214.2
GEOS-GCM	215.8	232.7	212.3
ICON-LEM	199.2	198.8	198.8
ICON-LEM-VER	198.1	198.6	199.9
ICON-LEM-LES	196.2	198.0	202.9
ICON-NWP	217.9	219.8	220.1
MESONH	226.3	228.0	228.1
MESONH-VER	227.0	226.7	227.3
MESONH-LES	223.7	223.7	223.7
MicroHH	210.0	211.3	210.1
MicroHH-VER	217.7	217.1	216.8
MicroHH-LES	200.4	201.1	201.2
MPAS	208.4	205.9	209.4
SAM-CRM	210.2	212.7	217.9
SAM-CRM-VER	213.1	211.0	211.9
SAM-CRM-LES	218.2	217.6	218.2
SCALE	202.7	204.2	204.3
UCLA-CRM	216.8	216.0	217.7
UKMO-GA7.1	210.6	212.0	208.5
UKMO-CASIM	199.5	198.5	199.0
UKMO-RA1-T-hrad	188.3	189.7	194.1
UKMO-RA1-T	188.2	192.1	198.8
UKMO-RA1-T-nocloud	193.9	196.1	196.2
WRF-COL-CRM	210.7	211.0	208.9
WRF-CRM	195.8	199.5	215.4
WRF-GCM-cps0	205.3	206.9	208.6
WRF-GCM-cps1	206.3	208.2	211.7
WRF-GCM-cps2	208.3	211.7	214.6
WRF-GCM-cps3	203.3	205.9	215.6
WRF-GCM-cps4	203.6	211.9	217.8
WRF-GCM-cps6	203.5	206.0	209.0
MEAN-ALL	208.3	210.6	213.0
IQR-ALL	11.0	11.9	9.3
RANGE-ALL	38.8	43.0	43.0
MEAN-PARAM.	208.1	211.7	212.1
IQR-PARAM.	9.4	8.5	6.8
RANGE-PARAM.	27.6	43.0	23.6
MEAN-EXPLICIT	208.3	210.3	213.3
IQR-EXPLICIT	11.8	12.4	10.9
RANGE-EXPLICIT	38.8	38.3	43.0

Table S4. `RCE_small` Anvil Cloud Height [km]

**Model**	295K	300K	305K
CAM5-GCM	9.7	11.1	12.5
CAM6-GCM	9.7	11.1	12.4
CM1	11.0	12.0	13.5
CM1-VER	10.4	11.6	13.2
CM1-LES	10.4	11.2	12.8
CNRM-CM6-1	10.9	12.4	14.0
DALES	11.0	11.0	11.0
DALES-VER	9.6	11.0	12.4
DALES-LES	10.4	11.0	12.8
DALES-damping	11.0	11.0	11.0
DALES-damping-VER	11.2	11.2	13.2
DAM	10.5	11.5	13.0
GEOS-GCM	9.9	9.1	13.7
ICON-LEM	12.5	14.0	16.0
ICON-LEM-VER	13.8	14.4	15.6
ICON-LEM-LES	14.6	14.8	14.6
ICON-NWP	9.5	10.5	12.0
MESONH	8.5	9.5	11.0
MESONH-VER	8.6	9.8	11.2
MESONH-LES	8.8	10.	11.4
MicroHH	11.5	12.5	14.0
MicroHH-VER	10.6	11.8	13.2
MicroHH-LES	12.4	13.4	14.6
MPAS	10.3	11.8	12.8
SAM-CRM	10.5	11.5	12.5
SAM-CRM-VER	10.2	11.8	13.4
SAM-CRM-LES	9.6	11.0	12.6
SCALE	11.0	12.0	13.5
UCLA-CRM	11.0	12.5	14.0
UKMO-GA7.1	10.9	12.0	13.8
UKMO-CASIM	12.1	13.6	15.3
UKMO-RA1-T-hrad	15.5	17.3	18.8
UKMO-RA1-T	15.5	16.7	17.3
UKMO-RA1-T-nocloud	13.6	14.7	17.3
WRF-COL-CRM	11.1	12.3	14.2
WRF-CRM	12.6	13.5	13.4
WRF-GCM-cps0	11.2	12.3	13.5
WRF-GCM-cps1	11.2	12.3	13.6
WRF-GCM-cps2	11.4	12.5	13.8
WRF-GCM-cps3	12.0	13.3	13.8
WRF-GCM-cps4	12.0	12.4	13.7
WRF-GCM-cps6	12.1	13.3	14.7
MEAN-ALL	11.2	12.2	13.6
IQR-ALL	1.7	2.0	1.4
RANGE-ALL	7.04	8.2	7.8
MEAN-PARAM.	11.0	12.0	13.6
IQR-PARAM.	1.4	1.34	1.2
RANGE-PARAM.	3.6	4.3	3.7
MEAN-EXPLICIT	11.3	12.3	13.6
IQR-EXPLICIT	2.0	2.4	1.8
RANGE-EXPLICIT	7.0	8.2	7.8

Table S5. `RCE_large` Anvil Cloud Fraction

**Model**	295K	300K	305K
CAM5-GCM	0.323	0.231	0.171
CAM6-GCM	0.337	0.244	0.822
CM1	0.312	0.240	0.173
CNRM-CM6-1	0.156	0.134	0.131
DAM	0.142	0.123	0.121
ECHAM6-GCM	0.142	0.179	0.179
FV3	0.190	0.173	0.231
GEOS-GCM	0.274	0.231	0.147
ICON-GCM	0.218	0.157	0.264
ICON-LEM	0.523	0.470	0.370
ICON-NWP	0.257	0.289	0.277
IPSL-CM6	0.081	0.080	0.074
MESONH	0.088	0.082	0.105
MPAS	0.126	0.128	0.120
NICAM	0.167	0.150	0.124
SAM0-UNICON	0.290	0.179	0.160
SAM-CRM	0.135	0.109	0.098
SAM-GCRM	0.154	0.112	0.112
SCALE	0.132	0.126	0.110
SP-CAM	0.169	0.166	0.157
SPX-CAM	0.192	0.179	0.164
UCLA-CRM	0.012	0.017	0.030
UKMO-GA7.1	0.278	0.214	0.157
UKMO-CASIM	0.161	0.103	0.060
UKMO-RA1-T	0.645	0.392	0.299
UKMO-RA1-T-nocloud	0.500	0.364	0.277
WRF-COL-CRM	0.279	0.244	0.215
WRF-CRM	0.133	0.143	0.137
WRF-GCM-cps0	0.432	0.377	0.319
WRF-GCM-cps1	0.548	0.569	0.550
WRF-GCM-cps2	0.368	0.349	0.347
WRF-GCM-cps3	0.420	0.379	0.381
WRF-GCM-cps4	0.394	0.412	0.427
WRF-GCM-cps6	0.471	0.394	0.300
MEAN-ALL	0.266	0.228	0.224
IQR-ALL	0.215	0.204	0.172
RANGE-ALL	0.633	0.553	0.792
MEAN-PARAM.	0.300	0.263	0.279
IQR-PARAM.	0.249	0.247	0.226
RANGE-PARAM.	0.536	0.553	0.792
MEAN-EXPLICIT	0.233	0.1919	0.168
IQR-EXPLICIT	0.134	0.1149	0.109
RANGE-EXPLICIT	0.633	0.453	0.340

Table S6. `RCE_large` Anvil Cloud Temperature [K]

**Model**	295K	300K	305K
CAM5-GCM	219.6	221.1	222.6
CAM6-GCM	217.4	216.6	205.6
CM1	201.6	208.2	209.4
CNRM-CM6-1	200.7	201.6	210.1
DAM	213.6	217.1	218.1
ECHAM6-GCM	206.0	207.7	207.3
FV3	216.5	217.6	221.6
GEOS-GCM	218.4	219.5	209.8
ICON-GCM	206.5	213.5	205.6
ICON-LEM	202.1	204.5	204.2
ICON-NWP	218.0	218.0	220.0
IPSL-CM6	221.4	224.9	225.2
MESONH	224.7	225.0	225.9
MPAS	210.5	217.5	220.7
NICAM	219.7	219.1	218.9
SAM0-UNICON	210.6	205.3	208.7
SAM-CRM	209.1	214.7	218.7
SAM-GCRM	210.3	221.6	216.6
SCALE	205.9	209.6	212.0
SP-CAM	219.7	220.1	223.0
SPX-CAM	221.5	215.6	219.4
UCLA-CRM	212.5	209.2	212.7
UKMO-GA7.1	200.4	205.4	216.1
UKMO-CASIM	222.7	224.2	226.5
UKMO-RA1-T	200.6	202.8	207.4
UKMO-RA1-T-nocloud	203.0	204.5	206.1
WRF-COL-CRM	211.3	213.0	215.0
WRF-CRM	184.8	198.1	205.0
WRF-GCM-cps0	206.3	210.1	215.0
WRF-GCM-cps1	206.4	209.4	209.6
WRF-GCM-cps2	210.0	215.1	213.0
WRF-GCM-cps3	207.1	210.5	215.0
WRF-GCM-cps4	209.9	207.6	213.4
WRF-GCM-cps6	204.8	211.5	211.4
MEAN-ALL	210.4	212.9	214.4
IQR-ALL	11.9	10.1	9.8
RANGE-ALL	39.9	26.9	22.3
MEAN-PARAM.	211.0	212.7	213.6
IQR-PARAM.	12.5	8.8	6.7
RANGE-PARAM.	36.7	26.8	21.0
MEAN-EXPLICIT	209.8	213.2	215.2
IQR-EXPLICIT	10.5	10.2	10.6
RANGE-EXPLICIT	39.9	26.9	22.3

Table S7. RCE_large Anvil Cloud Height [km]

**Model**	295K	300K	305K
CAM5-GCM	9.8	11.2	12.7
CAM6-GCM	9.7	11.1	18.7
CM1	12.0	12.5	14.5
CNRM-CM6-1	11.7	13.3	14.3
DAM	10.5	11.5	13.0
ECHAM6-GCM	11.5	12.8	15.3
FV3	10.8	12.5	13.9
GEOS-GCM	10.0	11.4	15.1
ICON-GCM	10.7	11.5	14.9
ICON-LEM	12.0	13.0	15.0
ICON-NWP	10.0	11.0	12.5
IPSL-CM6	9.7	11.2	13.1
MESONH	9.0	10.5	12.0
MPAS	10.5	11.0	12.0
NICAM	10.0	11.8	13.4
SAM0-UNICON	10.9	13.3	14.8
SAM-CRM	11.0	12.0	13.5
SAM-GCRM	11.0	11.5	14.0
SCALE	11.0	12.0	13.5
SP-CAM	10.0	11.3	12.8
SPX-CAM	10.0	12.5	14.0
UCLA-CRM	12.0	14.0	15.5
UKMO-GA7.1	12.6	13.8	14.4
UKMO-CASIM	9.6	10.9	12.4
UKMO-RA1-T	12.4	13.9	15.7
UKMO-RA1-T-nocloud	12.1	13.6	15.7
WRF-COL-CRM	11.0	12.3	13.8
WRF-CRM	14.5	13.9	14.5
WRF-GCM-cps0	12.1	13.5	15.0
WRF-GCM-cps1	12.2	13.4	16.3
WRF-GCM-cps2	11.3	12.6	14.9
WRF-GCM-cps3	12.2	13.5	15.0
WRF-GCM-cps4	11.3	13.3	14.9
WRF-GCM-cps6	12.1	13.5	16.3
MEAN-ALL	11.1	12.4	14.3
IQR-ALL	2.0	2.0	1.6
RANGE-ALL	5.5	3.5	6.7
MEAN-PARAM.	11.1	12.5	14.9
IQR-PARAM.	2.0	2.0	1.7
RANGE-PARAM.	3.6	3.3	6.7
MEAN-EXPLICIT	11.1	12.2	13.8
IQR-EXPLICIT	2.0	1.5	1.1
RANGE-EXPLICIT	5.5	3.5	3.7

Table S8. Net climate feedback parameter ($\lambda = dR_{\mathrm{TOA}}/dT$; $\mathrm{Wm^{-2}K^{-1}}$) and cloud radiative feedbacks ($\lambda_{\mathrm{cld}} = dCRE/dT$; $\mathrm{Wm^{-2}K^{-1}}$) in `RCE_small` simulations

Model	$\lambda_{295K-300K}$	$\lambda_{295K-305K}$	$\lambda_{300K-305K}$	$\lambda_{\mathrm{cld,295K-300K}}$	$\lambda_{\mathrm{cld,295K-305K}}$	$\lambda_{\mathrm{cld,300K-305K}}$
CAM5-GCM	1.78	-0.62	-3.02	2.67	0.37	-1.92
CAM6-GCM	1.79	-0.87	-3.52	3.00	0.14	-2.72
CM1	-0.97	-1.03	-1.09	0.15	0.02	-0.11
CM1-VER	-1.10	-1.10	-1.11	0.19	0.09	-0.02
CM1-LES	-1.21	-1.21	-1.21	0.02	-0.08	-0.18
CNRM-CM6-1	-1.59	-1.85	-2.12	-0.53	-1.05	-1.57
DALES	-1.15	-0.62	-0.10	0.55	0.53	0.51
DALES-VER	-1.67	-0.28	1.11	0.11	0.39	0.67
DALES-LES	-1.74	-1.19	-0.63	-0.24	-0.23	-0.22
DALES-damping	-1.05	-1.12	-1.18	0.82	0.59	0.36
DALES-damping-VER	-1.81	-1.09	-0.36	-0.21	0.01	0.23
DAM	-1.67	-1.57	-1.48	-0.41	-0.43	-0.45
GEOS-GCM	-0.87	-0.58	-0.29	0.67	0.61	0.55
ICON-LEM	-1.44	-1.28	-1.11	-0.14	-0.10	-0.06
ICON-LEM-VER	-1.47	-1.27	-1.07	-0.12	-0.06	-0.01
ICON-LEM-LES	-1.02	-1.03	-1.05	0.17	0.02	-0.13
ICON-NWP	-1.54	-1.51	-1.48	-0.04	-0.32	-0.60
MESONH	-1.23	-0.90	-0.57	-0.18	-0.02	0.13
MESONH-VER	-0.98	-0.83	-0.67	0.02	0.02	0.01
MESONH-LES	-0.73	-0.74	-0.75	0.27	0.06	-0.16
MicroHH	-0.09	-0.23	-0.36	-0.17	-0.21	-0.25
MicroHH-VER	-0.23	-0.53	-0.83	-0.23	-0.36	-0.49
MicroHH-LES	-0.36	-0.61	-0.85	-0.23	-0.35	-0.46
MPAS	-1.27	-1.35	-1.42	0.27	-0.01	-0.29
SAM-CRM	-1.28	-1.25	-1.22	0.12	0.04	-0.04
SAM-CRM-VER	-1.26	-1.13	-1.01	0.05	0.11	0.17
SAM-CRM-LES	-1.16	-1.22	-1.29	0.32	0.18	0.04
SCALE	-1.22	-1.31	-1.39	0.15	-0.06	-0.27
UCLA-CRM	-1.81	-1.60	-1.39	-0.55	-0.47	-0.39
UKMO-GA7.1	-2.09	-1.86	-1.64	-0.53	-0.40	-0.26
UKMO-CASIM	-1.23	-1.06	-0.90	0.13	0.01	-0.12
UKMO-RA1-T-hrad	-1.22	-0.98	-0.73	0.25	0.22	0.20
UKMO-RA1-T	-3.05	-2.33	-1.60	-0.36	-0.32	-0.29
UKMO-RA1-T-nocloud	-1.31	-1.05	-0.78	0.09	0.03	-0.02
WRF-COL-CRM	-0.89	-0.70	-0.50	0.62	0.54	0.46
WRF-CRM	-1.90	-1.27	-0.63	NaN	NaN	NaN
WRF-GCM-cps0	-2.30	-1.65	-1.00	-1.01	-0.48	0.04
WRF-GCM-cps1	-1.72	-1.63	-1.54	0.12	0.17	0.21
WRF-GCM-cps2	-1.14	-1.50	-1.87	0.66	0.18	-0.31
WRF-GCM-cps3	-0.52	-0.39	-0.26	1.50	1.55	1.60
WRF-GCM-cps4	-1.97	-1.76	-1.55	-0.29	0.01	0.30
WRF-GCM-cps6	1.96	0.78	-0.40	3.62	2.25	0.89
MEAN-ALL	-1.09	-1.08	-1.07	0.28	0.08	-0.12
IQR-ALL	0.67	0.57	0.77	0.48	0.39	0.48
MEAN-PARAM.	-0.60	-1.08	-1.56	0.90	0.30	-0.29
IQR-PARAM.	2.48	1.11	1.29	2.49	0.69	1.37
MEAN-EXPLICIT	-1.26	-1.08	-0.89	0.05	-0.01	-0.06
IQR-EXPLICIT	0.47	0.40	0.56	0.36	0.26	0.38

Table S9. Shortwave ($\lambda^{SW} = dR_{\text{TOA,SW}}/dT$; $\text{Wm}^{-2}\text{K}^{-1}$) and longwave ($\lambda^{LW} = dR_{\text{TOA,LW}}/dT$; $\text{Wm}^{-2}\text{K}^{-1}$) net climate feedback parameter in `RCE_small` simulations

Model	$\lambda^{SW}_{295K-300K}$	$\lambda^{SW}_{295K-305K}$	$\lambda^{SW}_{300K-305K}$	$\lambda^{LW}_{295K-300K}$	$\lambda^{LW}_{295K-305K}$	$\lambda^{LW}_{300K-305K}$
CAM5-GCM	-0.27	-1.22	-2.17	2.05	-0.85	0.60
CAM6-GCM	0.63	-1.27	-3.18	1.16	-0.35	0.41
CM1	-0.23	-0.04	0.16	-0.74	-1.26	-1.00
CM1-VER	-0.45	-0.23	0.00	-0.65	-1.11	-0.88
CM1-LES	0.26	0.34	0.42	-1.47	-1.63	-1.55
CNRM-CM6-1	-2.25	-2.74	-3.22	0.67	1.09	0.88
DALES	0.71	1.06	1.41	-1.86	-1.51	-1.68
DALES-VER	0.22	0.83	1.43	-1.89	-0.32	-1.11
DALES-LES	-0.14	0.25	0.64	-1.60	-1.27	-1.44
DALES-damping	0.97	1.12	1.27	-2.02	-2.45	-2.24
DALES-damping-VER	-0.11	0.51	1.13	-1.70	-1.49	-1.60
DAM	0.14	0.20	0.27	-1.81	-1.75	-1.78
GEOS-GCM	0.09	-1.21	-2.52	-0.96	2.22	0.63
ICON-LEM	0.16	0.20	0.24	-1.60	-1.35	-1.48
ICON-LEM-VER	0.14	0.22	0.31	-1.61	-1.38	-1.49
ICON-LEM-LES	0.63	0.44	0.26	-1.65	-1.30	-1.48
ICON-NWP	0.30	-0.02	-0.34	-1.83	-1.14	-1.49
MESONH	0.22	0.41	0.60	-1.45	-1.17	-1.31
MESONH-VER	0.62	0.67	0.73	-1.60	-1.40	-1.50
MESONH-LES	-0.09	0.17	0.42	-0.64	-1.17	-0.91
MicroHH	-0.12	-0.24	-0.36	0.03	0.00	0.01
MicroHH-VER	-0.30	-0.35	-0.40	0.06	-0.43	-0.18
MicroHH-LES	-0.24	-0.30	-0.37	-0.12	-0.49	-0.30
MPAS	0.74	0.39	0.03	-2.02	-1.45	-1.73
SAM-CRM	0.19	0.52	0.84	-1.47	-2.07	-1.77
SAM-CRM-VER	0.25	0.35	0.44	-1.51	-1.45	-1.48
SAM-CRM-LES	0.97	1.07	1.17	-2.13	-2.46	-2.29
SCALE	0.09	0.11	0.13	-1.31	-1.52	-1.42
UCLA-CRM	-0.01	0.19	0.39	-1.79	-1.79	-1.79
UKMO-GA7.1	0.86	0.81	0.75	-2.95	-2.39	-2.67
UKMO-CASIM	0.94	0.75	0.55	-2.17	-1.45	-1.81
UKMO-RA1-T-hrad	0.70	0.68	0.65	-1.93	-1.38	-1.65
UKMO-RA1-T	0.61	0.62	0.63	-3.67	-2.24	-2.95
UKMO-RA1-T-nocloud	0.66	0.62	0.57	-1.98	-1.35	-1.66
WRF-COL-CRM	1.23	0.97	0.72	-2.12	-1.22	-1.67
WRF-CRM	0.01	-0.62	-1.25	-1.91	0.61	-0.65
WRF-GCM-cps0	-0.19	-0.01	0.17	-2.11	-1.17	-1.64
WRF-GCM-cps1	0.31	0.18	0.05	-2.03	-1.60	-1.81
WRF-GCM-cps2	-0.08	0.23	0.54	-1.07	-2.41	-1.74
WRF-GCM-cps3	0.52	1.18	1.84	-1.03	-2.09	-1.56
WRF-GCM-cps4	1.01	0.35	-0.31	-2.98	-1.24	-2.11
WRF-GCM-cps6	4.46	3.29	2.11	-2.50	-2.51	-2.50
MEAN-ALL	0.34	0.25	0.16	-1.43	-1.33	-1.23
IQR-ALL	0.74	0.68	0.69	0.97	0.74	0.47
MEAN-PARAM.	0.46	-0.04	-0.54	-1.07	-1.05	-1.03
IQR-PARAM.	0.88	1.80	2.99	2.16	2.47	1.64
MEAN-EXPLICIT	0.29	0.35	0.41	-1.55	-1.30	-1.43
IQR-EXPLICIT	0.70	0.51	0.54	0.46	0.34	0.50

Table S10. Shortwave ($\lambda_{\text{cld}}^{SW} = dCRE_{SW}/dT$; $\text{Wm}^{-2}\text{K}^{-1}$) and longwave ($\lambda_{\text{cld}}^{LW} = dCRE_{LW}/dT$; $\text{Wm}^{-2}\text{K}^{-1}$) cloud feedbacks in `RCE_small` simulations

Model	$\lambda^{SW}_{\text{cld,295K}-300\text{K}}$	$\lambda^{SW}_{\text{cld,295K}-305\text{K}}$	$\lambda^{SW}_{\text{cld,300K}-305\text{K}}$	$\lambda^{LW}_{\text{cld,295K}-300\text{K}}$	$\lambda^{LW}_{\text{cld,295K}-305\text{K}}$	$\lambda^{LW}_{\text{cld,300K}-305\text{K}}$
CAM5-GCM	-0.43	-1.36	-2.30	3.09	1.74	0.38
CAM6-GCM	0.48	-1.40	-3.29	2.52	1.54	0.57
CM1	-0.36	-0.16	0.04	0.51	0.18	-0.15
CM1-VER	-0.58	-0.35	-0.12	0.77	0.44	0.11
CM1-LES	0.13	0.22	0.30	-0.11	-0.30	-0.48
CNRM-CM6-1	-2.40	-2.89	-3.38	1.87	1.84	1.81
DALES	0.63	0.62	0.60	-0.08	-0.09	-0.09
DALES-VER	0.13	0.37	0.61	-0.02	0.02	0.05
DALES-LES	-0.23	-0.21	-0.18	-0.01	-0.02	-0.04
DALES-damping	0.91	0.69	0.47	-0.08	-0.09	-0.10
DALES-damping-VER	-0.21	0.05	0.31	0.00	-0.04	-0.09
DAM	0.02	0.08	0.14	-0.43	-0.51	-0.59
GEOS-GCM	-0.01	-1.33	-2.64	0.68	1.94	3.19
ICON-LEM	0.05	0.08	0.11	-0.19	-0.18	-0.16
ICON-LEM-VER	0.02	0.10	0.18	-0.13	-0.16	-0.19
ICON-LEM-LES	0.51	0.32	0.13	-0.34	-0.30	-0.26
ICON-NWP	0.19	-0.14	-0.47	-0.23	-0.18	-0.12
MESONH	0.08	0.26	0.44	-0.27	-0.29	-0.31
MESONH-VER	0.47	0.52	0.57	-0.45	-0.50	-0.56
MESONH-LES	-0.24	0.01	0.26	0.51	0.04	-0.42
MicroHH	-0.12	-0.26	-0.39	-0.05	0.05	0.14
MicroHH-VER	-0.31	-0.37	-0.43	0.08	0.01	-0.06
MicroHH-LES	-0.25	-0.32	-0.39	0.02	-0.03	-0.07
MPAS	0.63	0.27	-0.09	-0.36	-0.28	-0.20
SAM-CRM	0.13	0.46	0.78	-0.01	-0.42	-0.82
SAM-CRM-VER	0.19	0.29	0.38	-0.14	-0.18	-0.22
SAM-CRM-LES	0.91	1.01	1.11	-0.59	-0.83	-1.07
SCALE	-0.03	-0.02	-0.01	0.18	-0.04	-0.26
UCLA-CRM	-0.14	0.06	0.26	-0.41	-0.53	-0.65
UKMO-GA7.1	0.76	0.70	0.64	-1.29	-1.09	-0.90
UKMO-CASIM	0.84	0.63	0.41	-0.71	-0.62	-0.53
UKMO-RA1-T-hrad	0.59	0.55	0.51	-0.33	-0.32	-0.31
UKMO-RA1-T	0.52	0.51	0.49	-0.88	-0.83	-0.78
UKMO-RA1-T-nocloud	0.55	0.49	0.43	-0.46	-0.46	-0.45
WRF-COL-CRM	1.17	0.92	0.66	-0.55	-0.38	-0.20
WRF-CRM	NaN	NaN	NaN	NaN	NaN	NaN
WRF-GCM-cps0	-0.25	-0.07	0.11	-0.76	-0.41	-0.07
WRF-GCM-cps1	0.25	0.12	-0.01	-0.13	0.04	0.22
WRF-GCM-cps2	-0.14	0.17	0.48	0.80	0.01	-0.79
WRF-GCM-cps3	0.45	1.11	1.77	1.05	0.44	-0.17
WRF-GCM-cps4	0.94	0.28	-0.38	-1.23	-0.27	0.69
WRF-GCM-cps6	4.40	3.22	2.05	-0.78	-0.97	-1.16
MEAN-ALL	0.25	0.13	0.00	0.03	-0.05	-0.12
IQR-ALL	0.69	0.65	0.62	0.51	0.43	0.43
MEAN-PARAM.	0.37	-0.13	-0.63	0.53	0.44	0.34
IQR-PARAM.	0.81	1.83	3.03	2.23	1.98	1.11
MEAN-EXPLICIT	0.21	0.22	0.24	-0.16	-0.23	-0.30
IQR-EXPLICIT	0.68	0.52	0.48	0.39	0.38	0.38

Table S11. Net climate feedback parameter ($\lambda = dR_{\mathrm{TOA}}/dT$; $\mathrm{Wm^{-2}K^{-1}}$) and cloud radiative feedbacks ($\lambda_{\mathrm{cld}} = dCRE/dT$; $\mathrm{Wm^{-2}K^{-1}}$) in `RCE_large` simulations

Model	$\lambda_{295K-300K}$	$\lambda_{295K-305K}$	$\lambda_{300K-305K}$	$\lambda_{\mathrm{cld,295K-300K}}$	$\lambda_{\mathrm{cld,295K-305K}}$	$\lambda_{\mathrm{cld,300K-305K}}$
CAM5-GCM	-4.70	-3.60	-2.50	-2.46	-1.65	-0.84
CAM6-GCM	-3.00	-2.79	-2.58	-1.03	-1.46	-1.89
CM1	-2.05	-1.90	-1.76	-0.24	-0.34	-0.43
CNRM-CM6-1	-3.31	-3.26	-3.20	-0.25	-0.73	-1.20
DAM	-1.69	-1.34	-0.99	-0.25	-0.11	0.04
ECHAM6-GCM	1.96	-1.96	-5.88	3.94	0.49	-2.96
FV3	-2.21	0.20	2.61	0.24	1.83	3.42
GEOS-GCM	-2.69	-3.36	-4.02	-0.72	0.57	1.86
ICON-GCM	-5.15	-4.60	-4.04	-2.64	-2.33	-2.02
ICON-LEM	-0.38	-0.34	-0.29	0.46	0.36	0.26
ICON-NWP	1.75	0.24	-1.28	1.86	0.71	-0.45
IPSL-CM6	-0.06	-0.20	-0.34	2.22	1.75	1.28
MESONH	-2.30	-1.05	0.20	0.29	0.31	0.32
MPAS	-1.25	-0.73	-0.21	0.53	0.32	0.11
NICAM	-2.46	-2.60	-2.74	-0.10	-0.37	-0.64
SAM0-UNICON	-4.25	-3.75	-3.26	-1.40	-1.10	-0.80
SAM-CRM	-2.63	-2.49	-2.36	-0.22	-0.14	-0.06
SAM-GCRM	-2.11	-2.26	-2.41	-0.29	-0.06	0.17
SCALE	-1.99	-2.04	-2.08	-0.22	-0.36	-0.50
SP-CAM	-2.76	-1.98	-1.20	-0.75	-0.20	0.36
SPX-CAM	-3.59	-2.54	-1.48	-1.08	-0.63	-0.17
UCLA-CRM	-2.21	0.15	2.51	-0.44	1.49	3.43
UKMO-GA7.1	-2.72	-2.12	-1.52	-0.08	0.00	0.08
UKMO-CASIM	-1.34	-0.86	-0.39	-0.28	-0.58	-0.88
UKMO-RA1-T	-2.34	-2.03	-1.72	0.02	-0.10	-0.21
UKMO-RA1-T-nocloud	-2.58	-1.85	-1.11	0.26	-0.02	-0.30
WRF-COL-CRM	-0.95	-0.92	-0.89	0.39	0.36	0.34
WRF-CRM	0.85	-0.52	-1.90	NaN	NaN	NaN
WRF-GCM-cps0	-0.18	-0.97	-1.76	2.42	1.52	0.61
WRF-GCM-cps1	-2.52	-2.98	-3.45	-0.60	-0.89	-1.17
WRF-GCM-cps2	-1.35	-2.01	-2.67	1.26	0.46	-0.34
WRF-GCM-cps3	-2.15	-0.98	0.19	0.41	1.51	2.62
WRF-GCM-cps4	-2.62	-2.37	-2.12	-0.06	0.07	0.21
WRF-GCM-cps6	-4.60	-4.91	-5.23	-1.06	-1.64	-2.23
MEAN-ALL	-2.05	-1.90	-1.76	0.00	-0.03	-0.06
IQR-ALL	1.37	1.65	1.74	0.98	1.04	1.13
MEAN-PARAM.	-2.57	-2.61	-2.65	-0.11	-0.25	-0.39
IQR-PARAM.	1.45	1.38	1.93	1.47	1.59	1.56
MEAN-EXPLICIT	-1.52	-1.20	-0.87	0.13	0.21	0.29
IQR-EXPLICIT	1.05	1.51	1.60	0.56	0.55	0.71

Table S12. Shortwave ($\lambda^{SW} = dR_{\mathrm{TOA,SW}}/dT$; $\mathrm{Wm^{-2}K^{-1}}$) and longwave ($\lambda^{LW} = dR_{\mathrm{TOA,LW}}/dT$; $\mathrm{Wm^{-2}K^{-1}}$) net climate feedback parameter in `RCE_large` simulations

Model	$\lambda^{SW}_{295K-300K}$	$\lambda^{SW}_{295K-305K}$	$\lambda^{SW}_{300K-305K}$	$\lambda^{LW}_{295K-300K}$	$\lambda^{LW}_{295K-305K}$	$\lambda^{LW}_{300K-305K}$
CAM5-GCM	-1.02	-0.40	0.22	-3.68	-3.20	-2.72
CAM6-GCM	0.10	-0.17	-0.44	-3.10	-2.62	-2.14
CM1	0.16	0.24	0.32	-2.20	-2.14	-2.08
CNRM-CM6-1	0.23	-0.55	-1.33	-3.55	-2.71	-1.87
DAM	0.04	0.02	-0.01	-1.73	-1.36	-0.98
ECHAM6-GCM	4.11	0.20	-3.72	-2.16	-2.16	-2.16
FV3	0.53	1.80	3.06	-2.74	-1.60	-0.45
GEOS-GCM	0.25	2.74	5.23	-2.95	-6.10	-9.26
ICON-GCM	-1.98	-1.34	-0.71	-3.17	-3.25	-3.34
ICON-LEM	0.33	0.28	0.23	-0.71	-0.62	-0.52
ICON-NWP	0.74	0.49	0.24	1.01	-0.25	-1.52
IPSL-CM6	3.78	2.96	2.15	-3.83	-3.16	-2.49
MESONH	1.19	0.43	-0.33	-3.48	-1.48	0.53
MPAS	0.60	0.45	0.31	-1.84	-1.18	-0.52
NICAM	0.52	0.44	0.36	-2.98	-3.04	-3.10
SAM0-UNICON	0.46	0.29	0.12	-4.71	-4.04	-3.38
SAM-CRM	0.14	0.14	0.14	-2.77	-2.64	-2.51
SAM-GCRM	0.55	0.18	-0.18	-2.66	-2.44	-2.23
SCALE	-0.04	-0.13	-0.21	-1.95	-1.91	-1.87
SP-CAM	-0.72	-0.14	0.43	-2.05	-1.84	-1.63
SPX-CAM	-0.96	-0.42	0.11	-2.64	-2.11	-1.59
UCLA-CRM	-0.16	1.85	3.86	-2.05	-1.70	-1.36
UKMO-GA7.1	1.38	1.46	1.55	-4.11	-3.58	-3.06
UKMO-CASIM	0.99	0.50	0.00	-2.33	-1.36	-0.39
UKMO-RA1-T	1.13	0.88	0.63	-3.47	-2.91	-2.35
UKMO-RA1-T-nocloud	1.46	0.90	0.34	-4.04	-2.74	-1.45
WRF-COL-CRM	0.37	0.42	0.48	-1.32	-1.34	-1.36
WRF-CRM	1.36	0.77	0.18	-0.50	-1.29	-2.08
WRF-GCM-cps0	2.97	2.02	1.08	-3.15	-2.99	-2.84
WRF-GCM-cps1	-0.99	-0.93	-0.87	-1.53	-2.06	-2.58
WRF-GCM-cps2	1.84	0.85	-0.14	-3.18	-2.86	-2.53
WRF-GCM-cps3	0.88	1.86	2.85	-3.02	-2.84	-2.65
WRF-GCM-cps4	0.27	0.13	-0.01	-2.90	-2.50	-2.11
WRF-GCM-cps6	0.99	-0.27	-1.53	-5.59	-4.65	-3.71
MEAN-ALL	0.63	0.53	0.42	-2.68	-2.43	-2.19
IQR-ALL	0.99	0.96	0.64	1.35	1.35	1.17
MEAN-PARAM.	0.68	0.49	0.29	-3.25	-3.10	-2.94
IQR-PARAM.	2.10	1.86	1.78	0.79	0.75	0.92
MEAN-EXPLICIT	0.58	0.57	0.55	-2.10	-1.77	-1.43
IQR-EXPLICIT	0.84	0.53	0.36	1.04	1.10	1.56

Table S13. Shortwave ($\lambda^{SW}_{\text{cld}} = dCRE_{SW}/dT$; $\text{Wm}^{-2}\text{K}^{-1}$) and longwave ($\lambda^{LW}_{\text{cld}} = dCRE_{LW}/dT$; $\text{Wm}^{-2}\text{K}^{-1}$) cloud feedbacks in `RCE_large` simulations

Model	$\lambda^{SW}_{\text{cld,295K−300K}}$	$\lambda^{SW}_{\text{cld,295K−305K}}$	$\lambda^{SW}_{\text{cld,300K−305K}}$	$\lambda^{LW}_{\text{cld,295K−300K}}$	$\lambda^{LW}_{\text{cld,295K−305K}}$	$\lambda^{LW}_{\text{cld,300K−305K}}$
CAM5-GCM	-1.14	-0.52	0.10	-1.33	-1.13	-0.94
CAM6-GCM	0.00	-0.29	-0.57	-1.03	-1.18	-1.33
CM1	0.04	0.12	0.19	-0.29	-0.45	-0.62
CNRM-CM6-1	0.12	-0.68	-1.48	-0.37	-0.05	0.28
DAM	-0.09	-0.11	-0.14	-0.16	0.01	0.17
ECHAM6-GCM	4.02	0.10	-3.82	-0.08	0.39	0.86
FV3	0.46	1.71	2.96	-0.22	0.12	0.46
GEOS-GCM	0.17	2.79	5.42	-0.89	-2.22	-3.56
ICON-GCM	-2.10	-1.48	-0.85	-0.54	-0.86	-1.17
ICON-LEM	0.19	0.13	0.07	0.27	0.23	0.19
ICON-NWP	0.59	0.35	0.10	1.26	0.36	-0.55
IPSL-CM6	3.67	2.85	2.03	-1.45	-1.10	-0.75
MESONH	1.07	0.28	-0.52	-0.78	0.03	0.84
MPAS	0.49	0.33	0.17	0.04	-0.01	-0.06
NICAM	0.35	0.28	0.22	-0.45	-0.66	-0.86
SAM0-UNICON	0.34	0.17	0.00	-1.74	-1.27	-0.80
SAM-CRM	0.08	0.08	0.08	-0.30	-0.22	-0.14
SAM-GCRM	0.49	0.12	-0.24	-0.78	-0.18	0.42
SCALE	-0.17	-0.26	-0.35	-0.05	-0.10	-0.15
SP-CAM	-0.76	-0.19	0.38	0.01	-0.01	-0.02
SPX-CAM	-1.00	-0.47	0.06	-0.08	-0.15	-0.23
UCLA-CRM	-0.29	1.72	3.73	-0.15	-0.22	-0.30
UKMO-GA7.1	1.27	1.35	1.42	-1.35	-1.35	-1.35
UKMO-CASIM	0.87	0.35	-0.16	-1.15	-0.93	-0.72
UKMO-RA1-T	1.01	0.75	0.48	-0.99	-0.84	-0.69
UKMO-RA1-T-nocloud	1.37	0.77	0.18	-1.11	-0.79	-0.48
WRF-COL-CRM	0.31	0.36	0.42	0.08	0.00	-0.08
WRF-CRM	NaN	NaN	NaN	NaN	NaN	NaN
WRF-GCM-cps0	2.91	1.96	1.01	-0.49	-0.44	-0.40
WRF-GCM-cps1	-1.06	-1.00	-0.94	0.46	0.12	-0.23
WRF-GCM-cps2	1.78	0.78	-0.22	-0.52	-0.32	-0.12
WRF-GCM-cps3	0.82	1.80	2.78	-0.41	-0.29	-0.16
WRF-GCM-cps4	0.21	0.07	-0.07	-0.27	0.00	0.28
WRF-GCM-cps6	0.93	-0.33	-1.59	-1.99	-1.31	-0.64
MEAN-ALL	0.51	0.42	0.33	-0.51	-0.45	-0.39
IQR-ALL	0.93	0.96	0.66	0.91	0.85	0.70
MEAN-PARAM.	0.60	0.41	0.21	-0.71	-0.66	-0.60
IQR-PARAM.	2.03	1.82	1.86	1.05	1.13	0.82
MEAN-EXPLICIT	0.42	0.44	0.45	-0.30	-0.23	-0.16
IQR-EXPLICIT	0.59	0.34	0.41	0.75	0.52	0.74

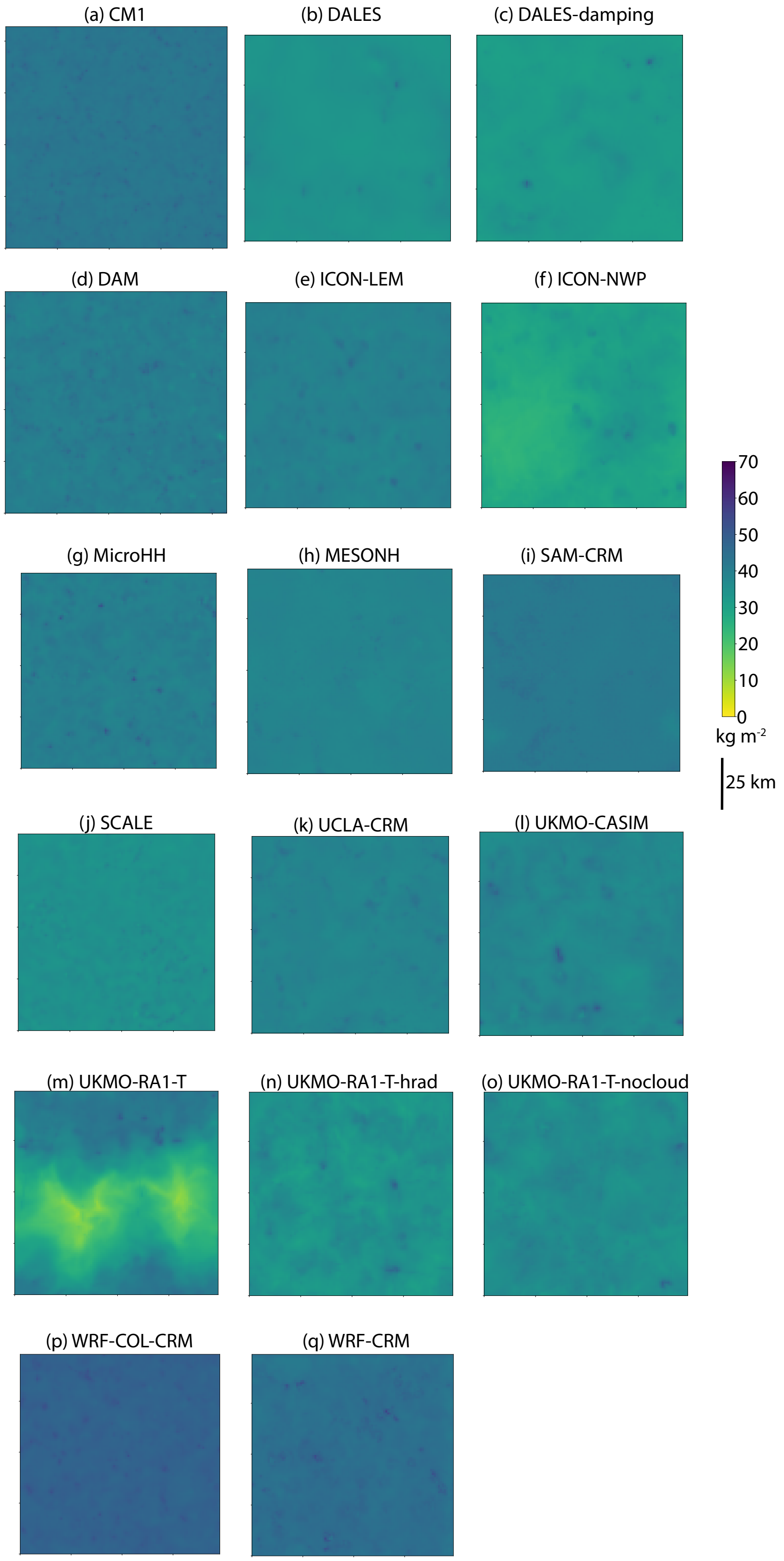

Figure S1. Hourly-averaged precipitable water (kg m^{-2}) at day 80 of the `RCE_small300` simulation for all cloud-resolving models.

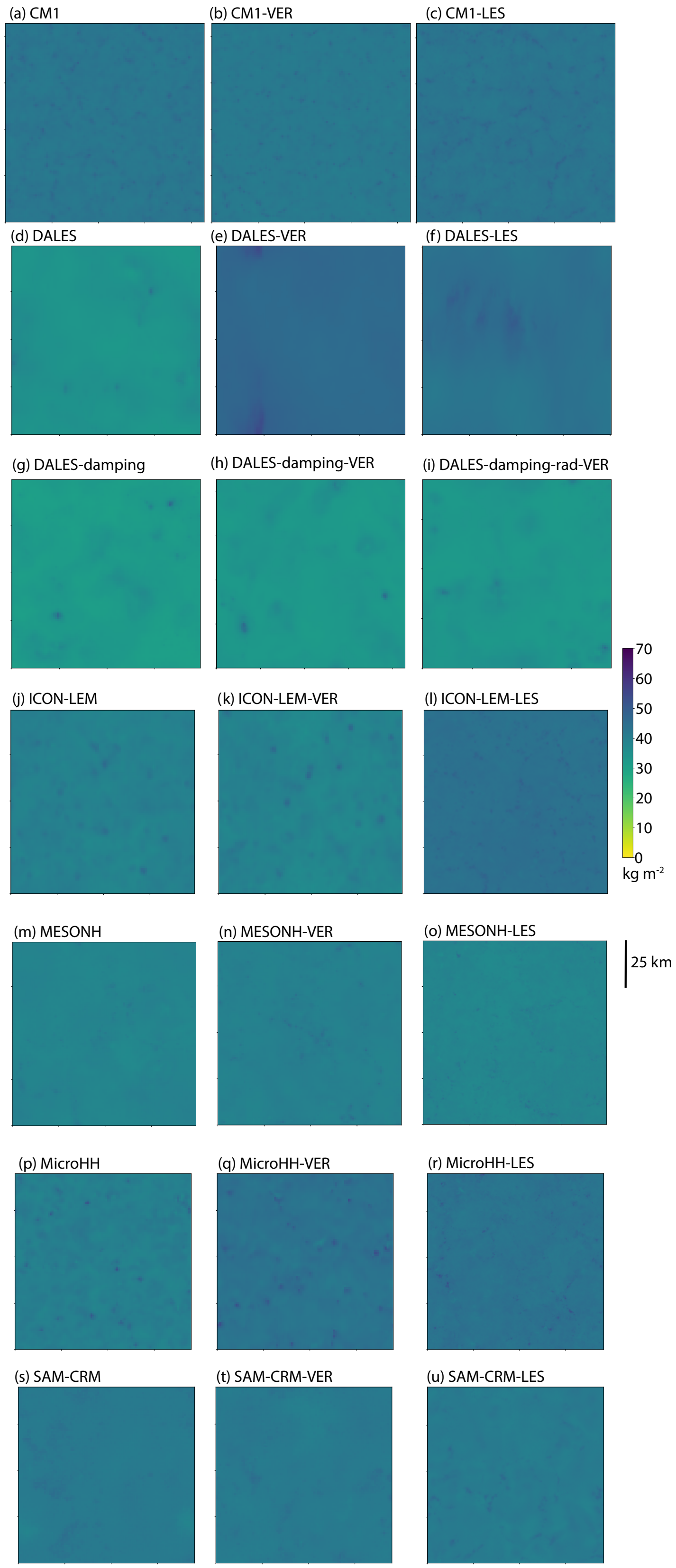

Figure S2. Hourly-averaged precipitable water (kg m^{-2})at day 80 of the `RCE_small300` (left column) and day 50 of the `RCE_small_vert300` (middle column) and `RCE_small_les300` (right column) simulations for CM1, DALES, DALES-damping, ICON_LEM, MESONH, MicroHH, and SAM. Note that the radiative properties of ice clouds were erroneously configured in DALES and DALES-damping. DALES-damping-rad is a corrected version, shown for reference (note that it is the `RCE_small_vert300` simulation that is shown despite it being in the column with LES simulations).

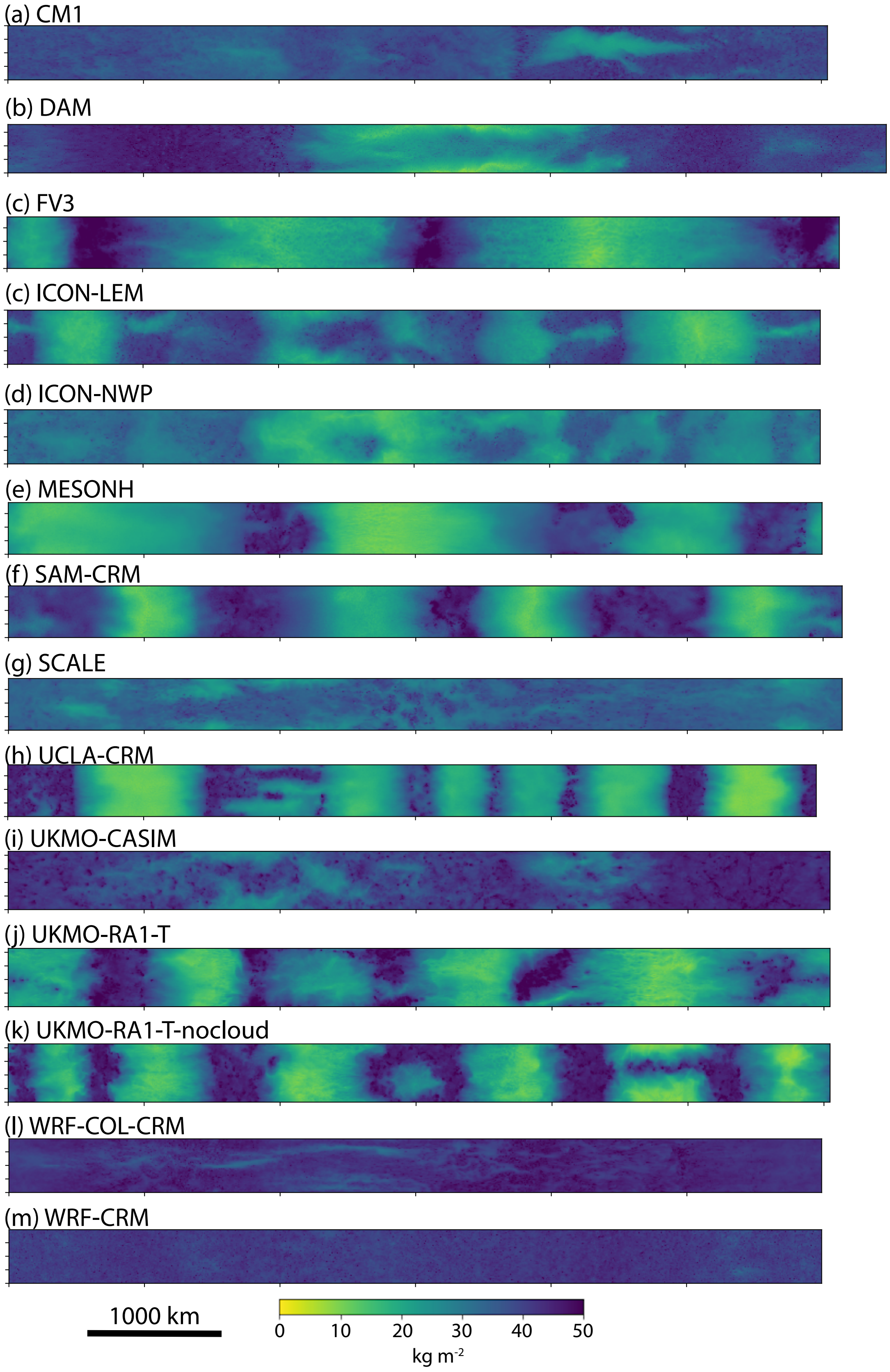

Figure S3. Hourly-averaged precipitable water (kg m^{-2}) at day 80 of the `RCE_large300` simulation for all cloud-resolving models.

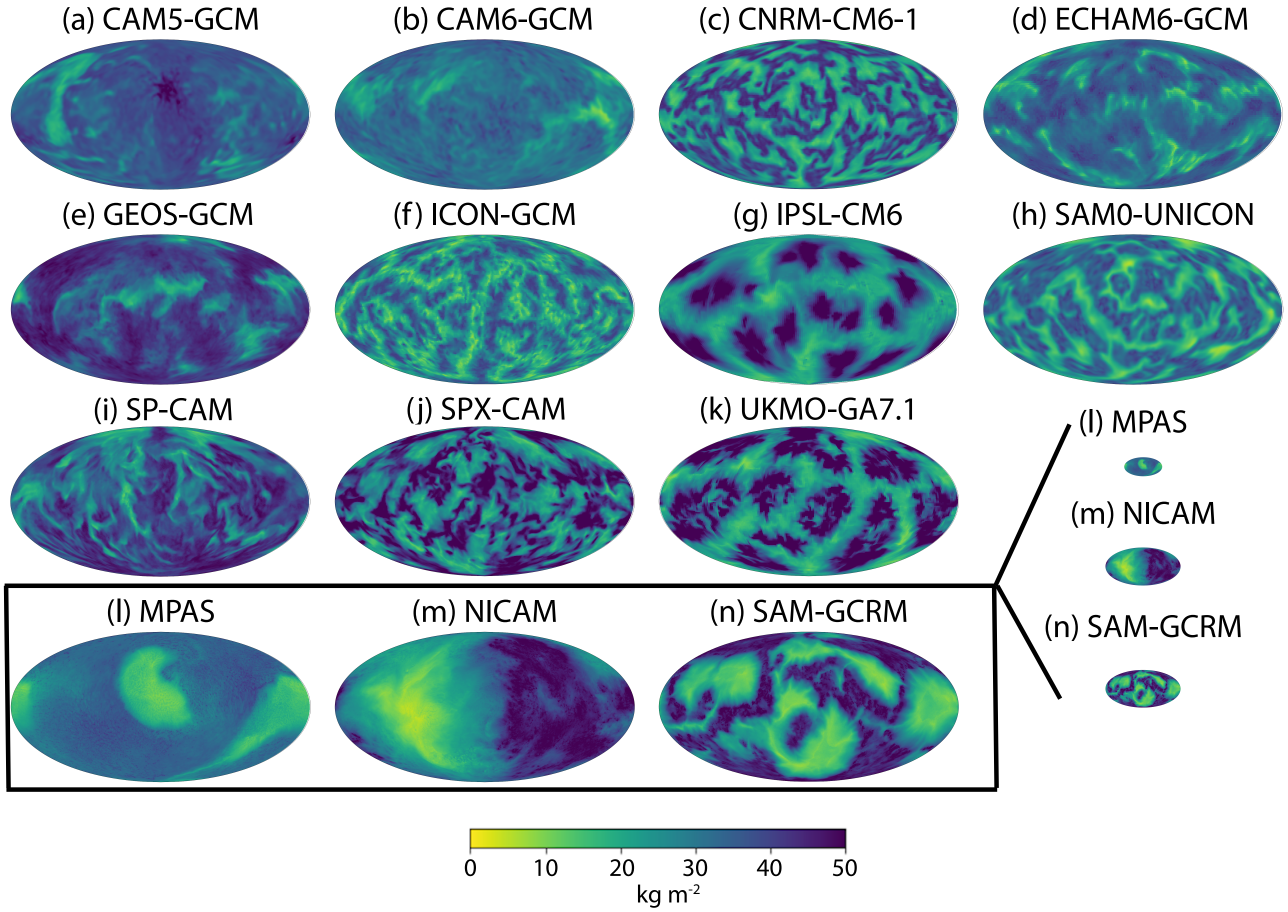

Figure S4. Hourly-averaged precipitable water (kg m^{-2}) at day 80 of the `RCE_large300` simulation for all global models. All models shown are GCMs with parameterized convection except MPAS, NICAM, and SAM, which are global cloud-resolving models that employ reduced Earth radius of $R_E/8$, $R_E/4$, and $R_E/4$, respectively, and are shown to scale and, in the box, zoomed in. IPSL-CM6 is daily-averaged.

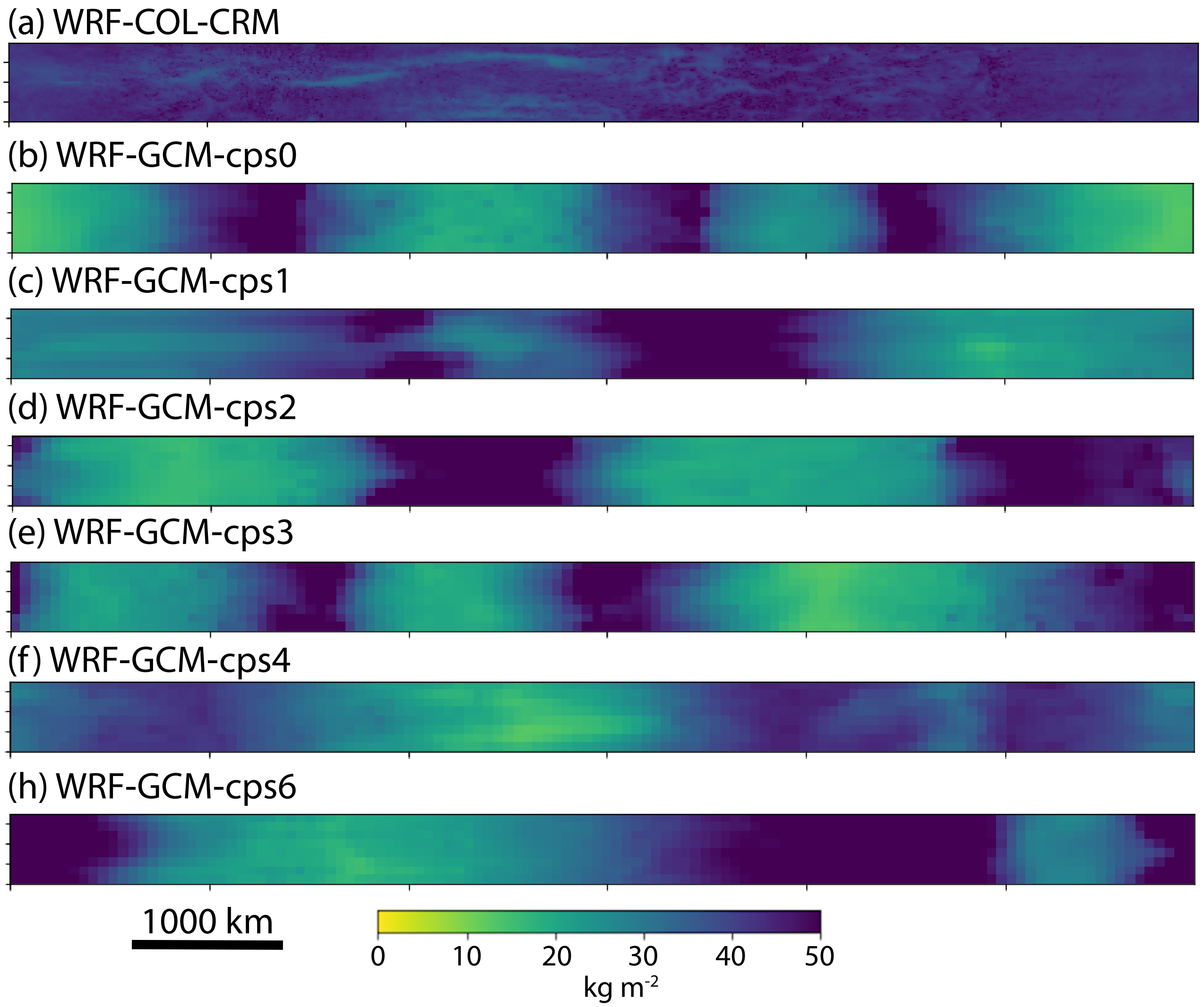

Figure S5. Hourly-averaged precipitable water (kg m^{-2}) at day 80 of the `RCE_large300` simulation for all versions of WRF 3.5.1. The top panel for the WRF-COL-CRM configuration is the same is in Figure S3. The other panels show WRF in the cartesian `RCE_large300` configuration but with 50 km grid spacing and convective parameterizations.

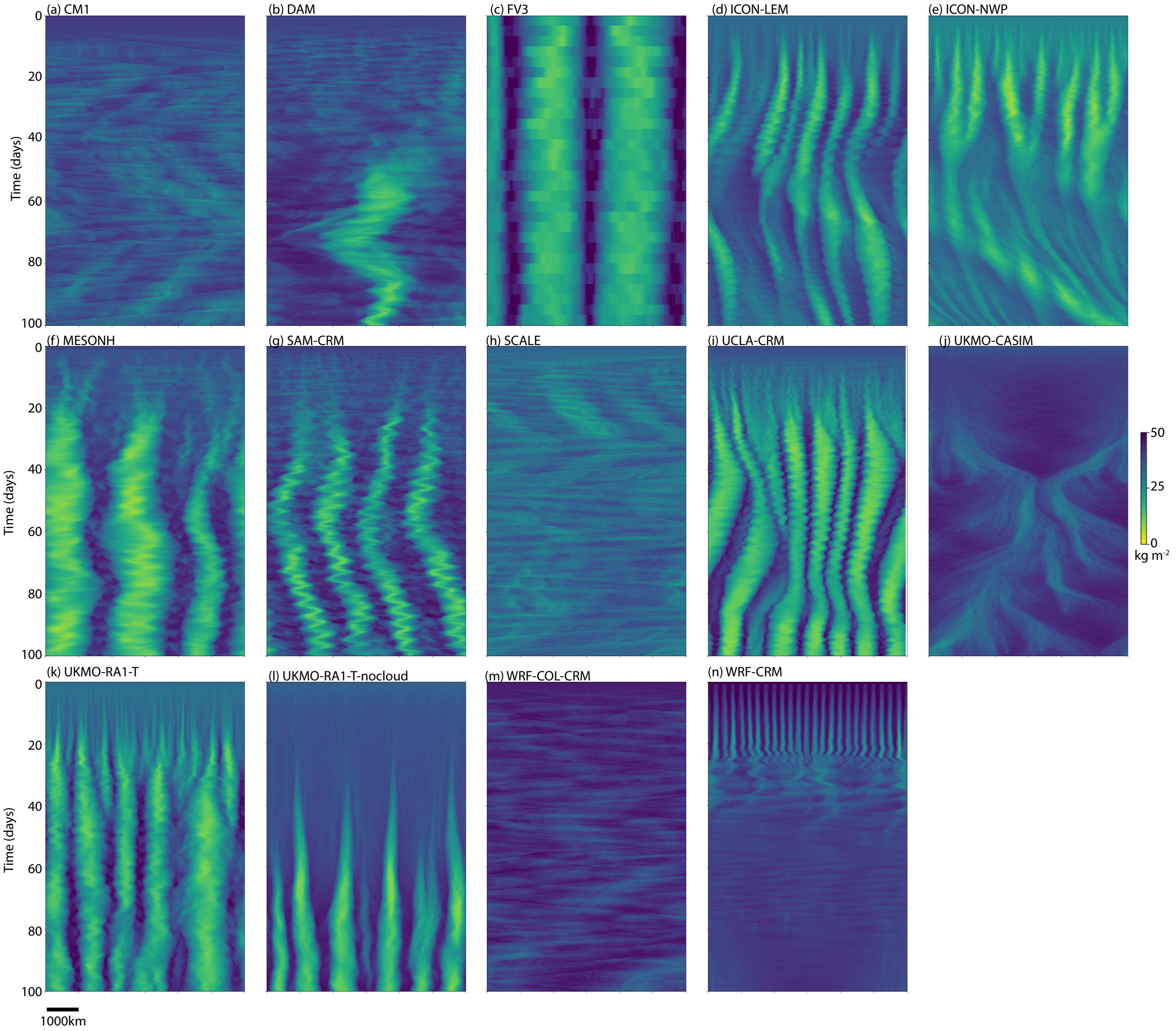

Figure S6. Temporal evolution of hourly- and y-averaged precipitable water (kg m^{-2}) in the RCE_large300 simulations for CRMs over the first 100 days of simulation (y-axis) and the first 6000 km of the domain (x-axis).

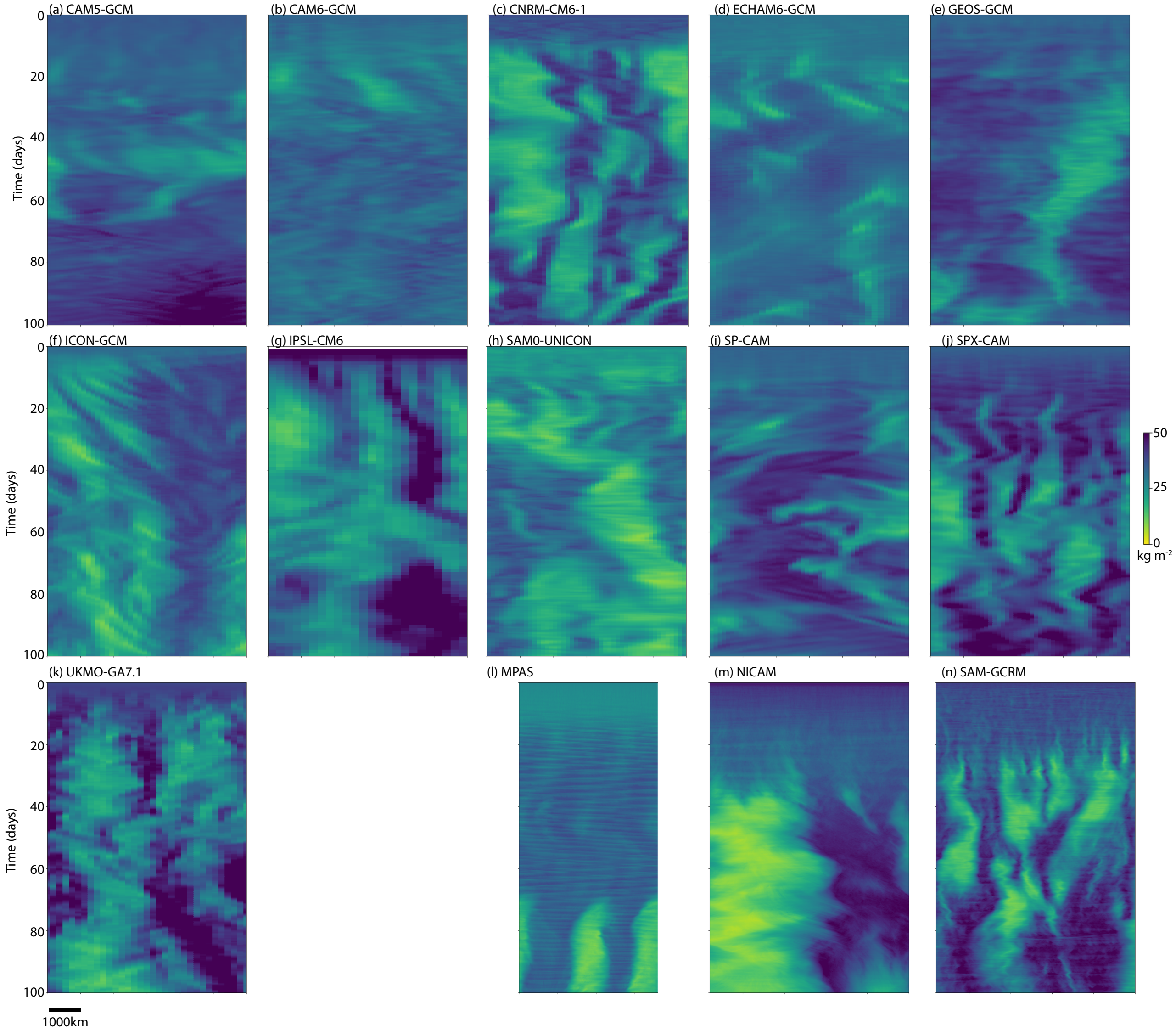

Figure S7. Temporal evolution of hourly- and y-averaged precipitable water (kg m^{-2}) in the RCE_large300 simulations for GCMs (a-k) and GCRMs (l-n) over the first 100 days of simulation (y-axis) over a ∼6000 km zonal segment of the globe (x-axis). The PW is averaged meridionally within ∼400 km of the equator. Since MPAS employs a sphere of $R_E/8$, a ∼4500 km zonal segment is shown, which is the entire domain. IPSL-CM6 is daily-averaged.

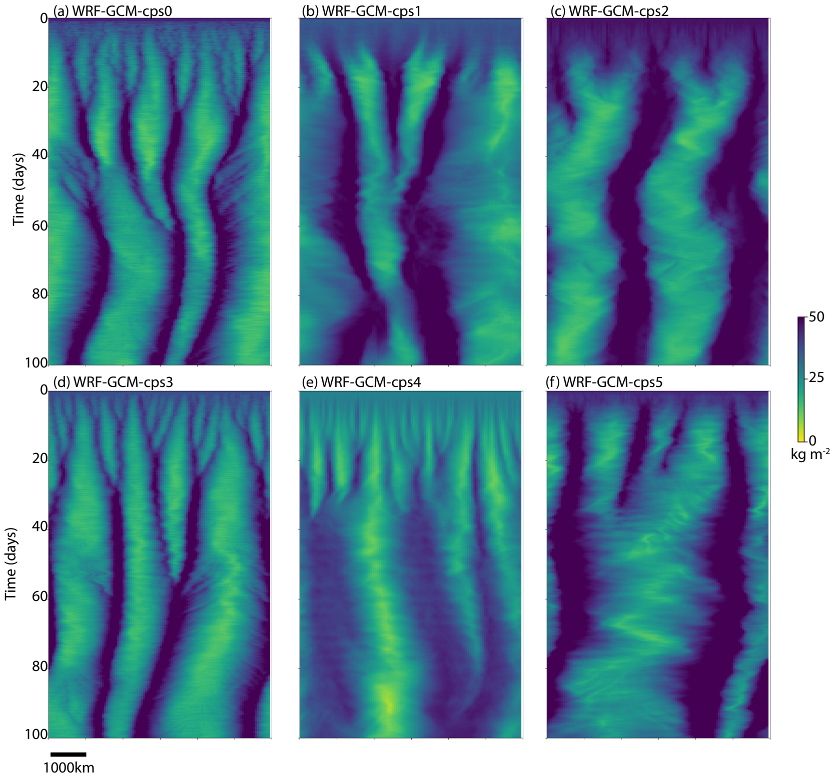

Figure S8. Temporal evolution of hourly- and y-averaged precipitable water (kg m^{-2}) in the `RCE_large300` simulations for WRF-GCM over the first 100 days of simulation (y-axis) and the first 6000 km of the domain (x-axis).

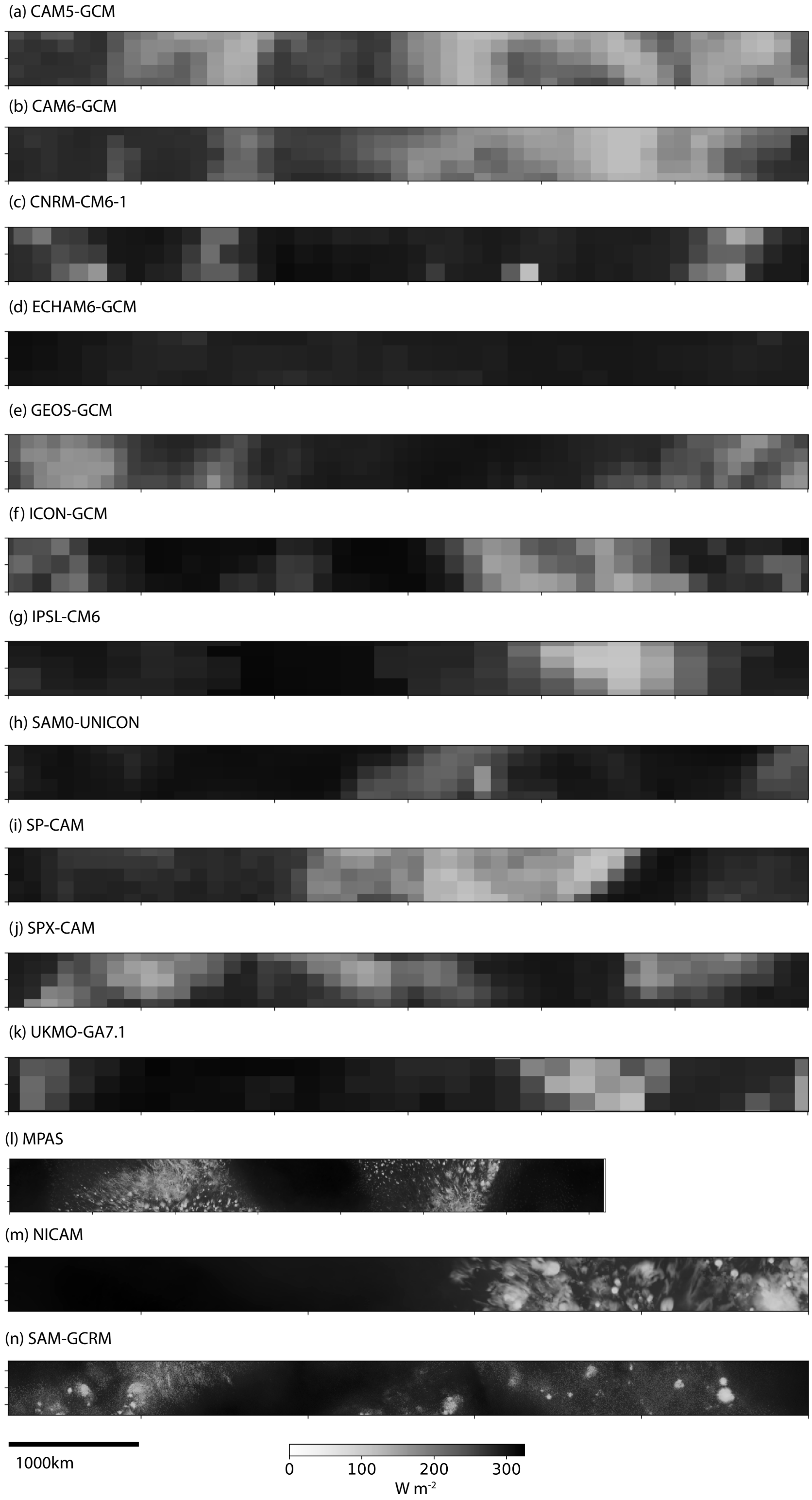

Figure S9. Hourly-averaged outgoing longwave radiation (Wm^{-2}) at day 80 of the RCE_large300 simulation for all GCMs, over a ∼6000 km x ∼400 km tropical band centered on the equator, to match the domain size of the CRM simulations (compare to Figure 4 in the main text). Since MPAS employs a sphere of $R_E/8$, a ∼4500 km zonal segment is shown, which is the entire domain. IPSL-CM6 is daily-averaged.

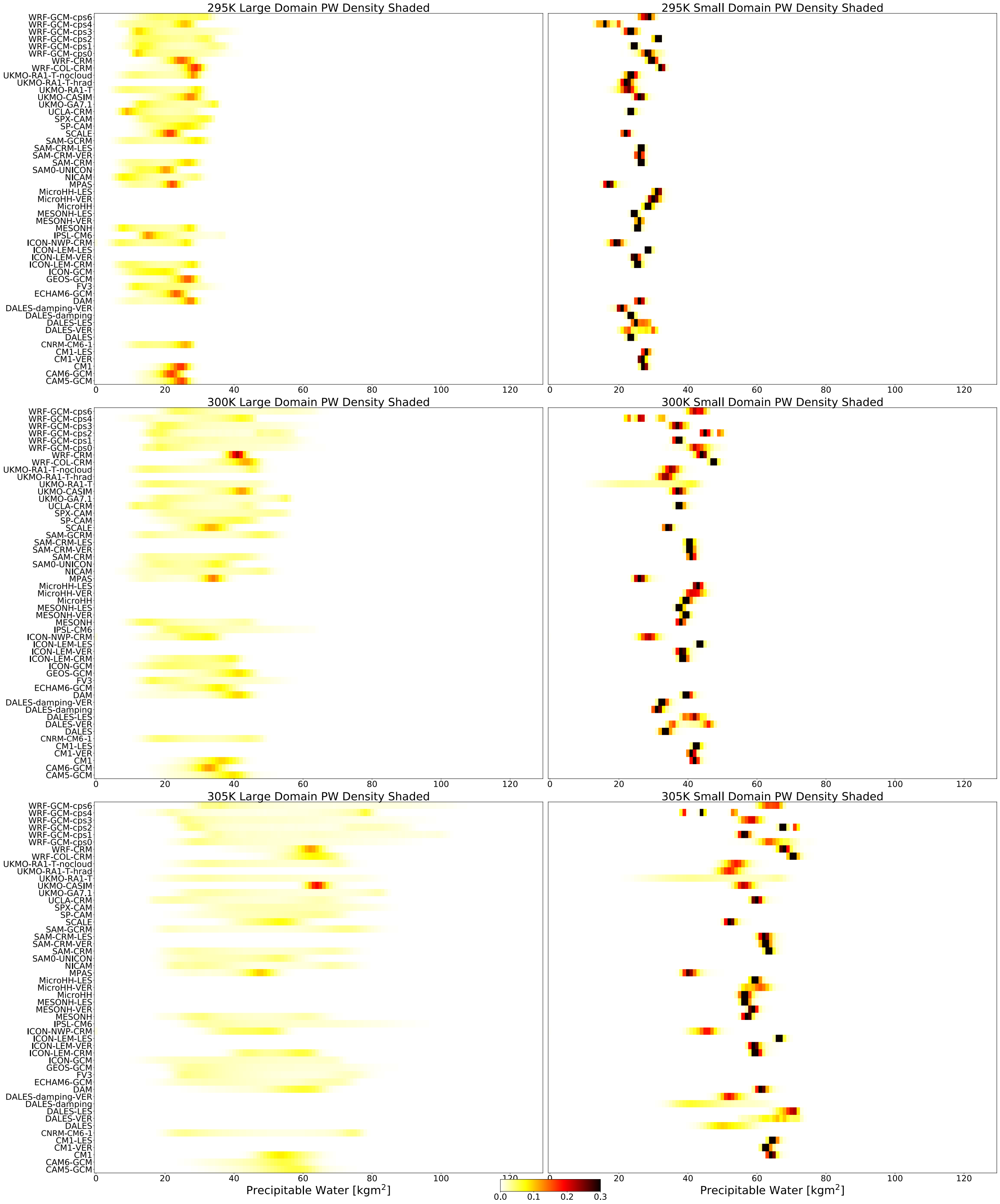

Figure S10. Probabability density function of precipitable water (PW; kg m^{-2}) in `RCE_large` simulations (left panels) and `RCE_small` simulations (right panels) at 295 K (top), 300 K (middle), and 305 K (bottom). Each row represents the PDF for a particular model (ordered alphabetically). A row with no shading means that that model did not perform that particular simulation.

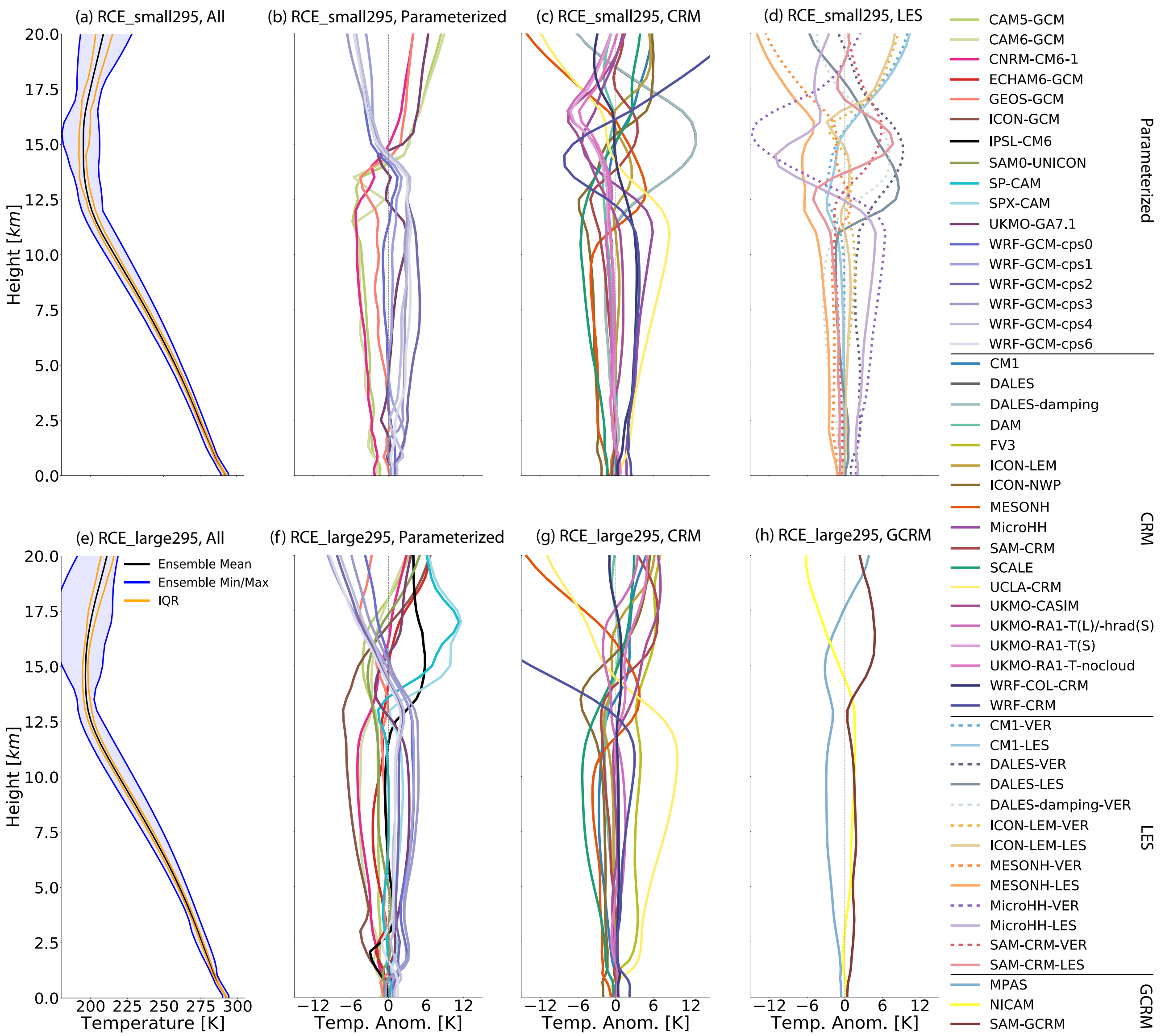

Figure S11. Horizontal-mean temperature profile, averaged in time excluding the first 75 days of simulation of the `RCE_small` (top row, a-d) and `RCE_large` (bottom row, e-h) simulations at 295 K. The first column (a,e) includes all models that performed each type of simulation, where the black line is the ensemble mean, the blue shading shows the range across all models, and the orange lines indicate the interquartile range (IQR). The other columns display the temperature anomaly in each sub-group of models as an anomaly from the ensemble mean of that sub-group, for models with parameterized convection (second column; b,f), CRMs (third column; c,g), models that performed `RCE_small_vert` (dashed) and `RCE_small_les` (solid) simulations (panel d; `RCE_small_les` simulations are averaged over days 25-50), and GCRMs (panel h).

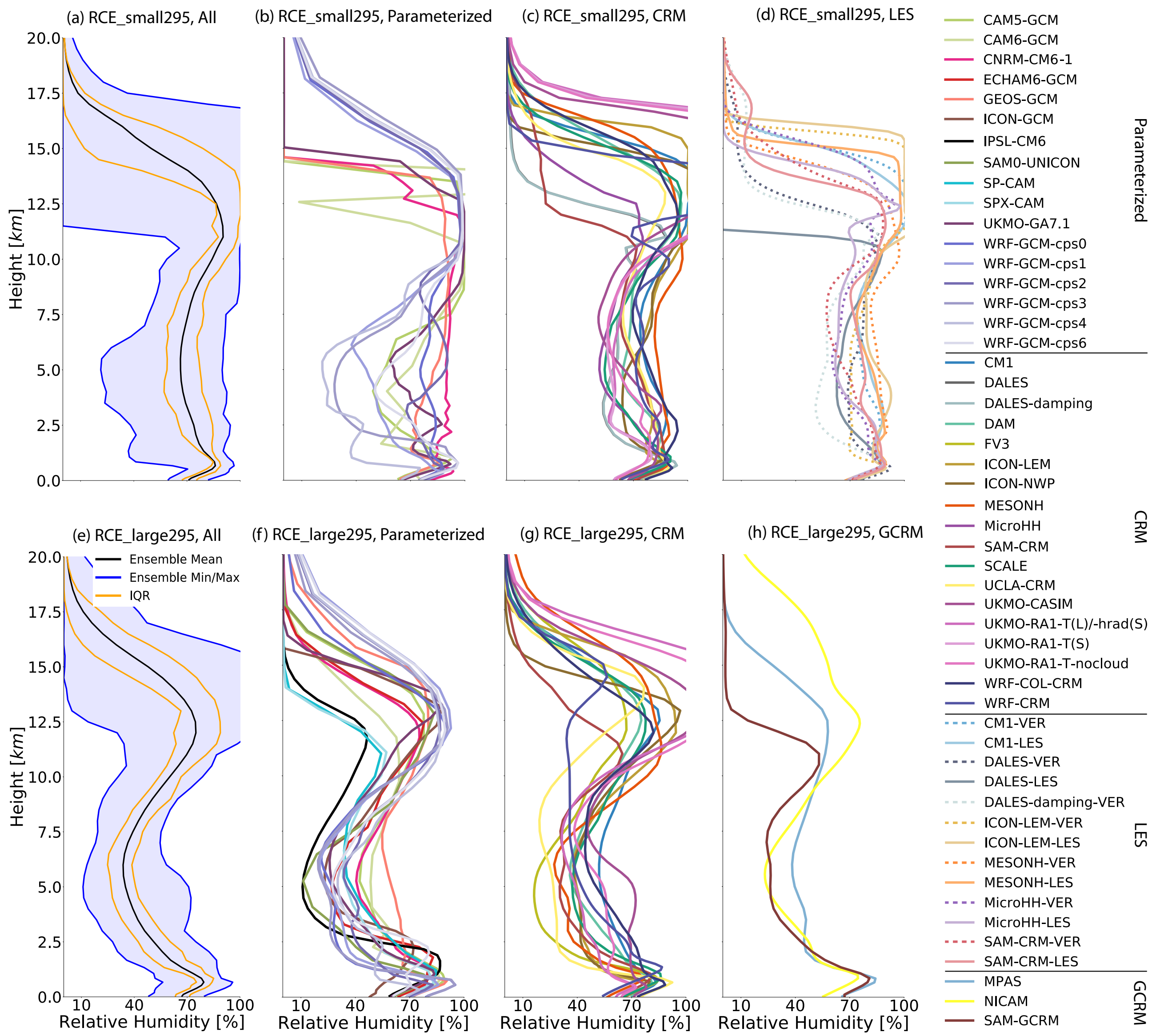

Figure S12. Horizontal-mean relative humidity profile, averaged in time excluding the first 75 days of simulation of the `RCE_small` (top row, a-d) and `RCE_large` (bottom row, e-h) simulations at 295 K. The first column (a,e) includes all models that performed each type of simulation, where the black line is the ensemble mean, the blue shading shows the range across all models, and the orange lines indicate the interquartile range (IQR). The other columns display each sub-group of models: models with parameterized convection (second column; b,f), CRMs (third column; c,g), models that performed `RCE_small_vert` (dashed) and `RCE_small_les` (solid) simulations (panel d; `RCE_small_les` simulations are averaged over days 25-50), and GCRMs (panel h).

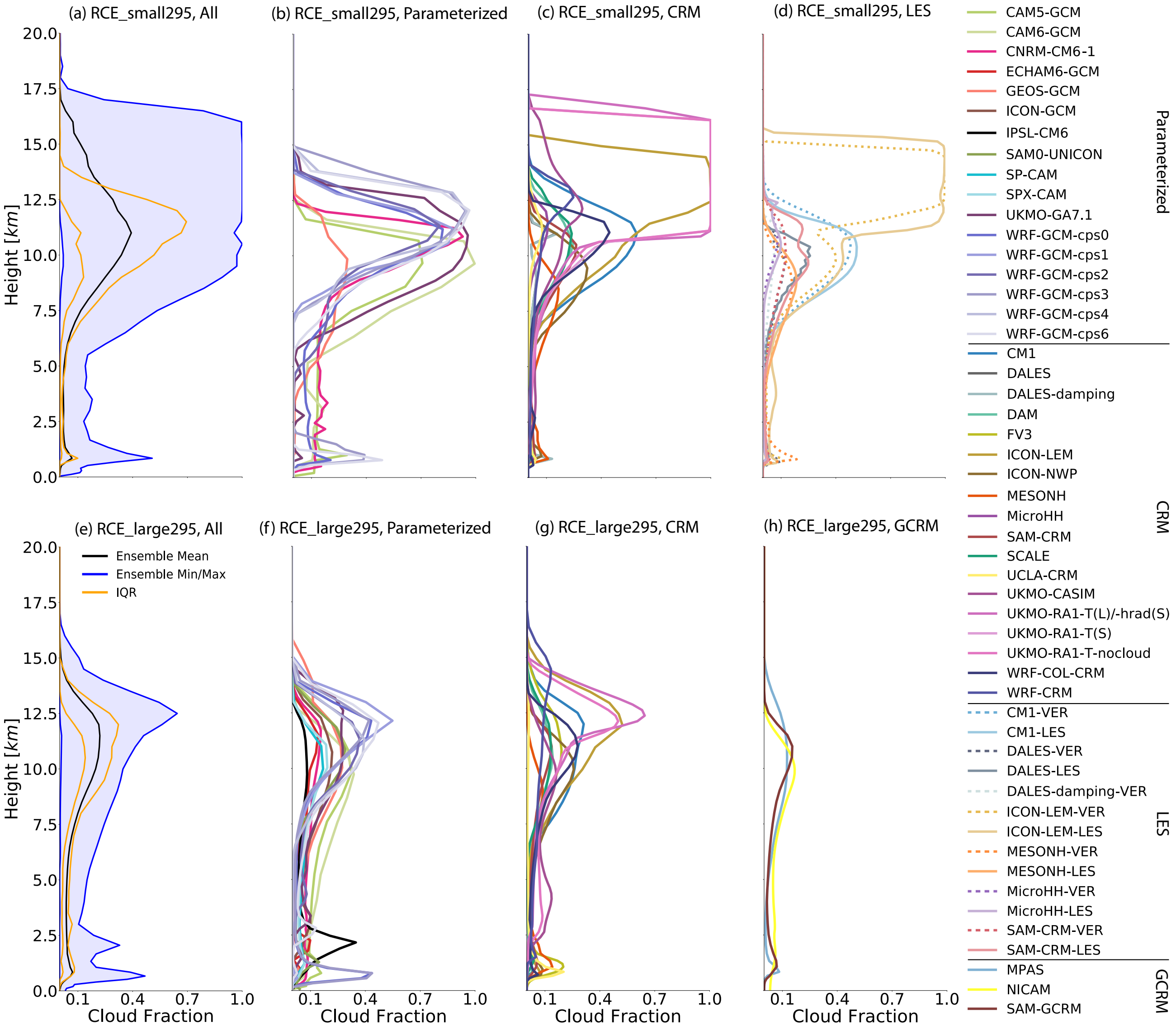

Figure S13. Domain-wide cloud fraction profile, averaged in time excluding the first 75 days of simulation of the `RCE_small` (top row, a-d) and `RCE_large` (bottom row, e-h) simulations at 295 K. The first column (a,e) includes all models that performed each type of simulation, where the black line is the ensemble mean, the blue shading shows the range across all models, and the orange lines indicate the interquartile range (IQR). The other columns display each sub-group of models: models with parameterized convection (second column; b,f), CRMs (third column; c,g), models that performed `RCE_small_vert` (dashed) and `RCE_small_les` (solid) simulations (panel d; `RCE_small_les` simulations are averaged over days 25-50), and GCRMs (panel h).

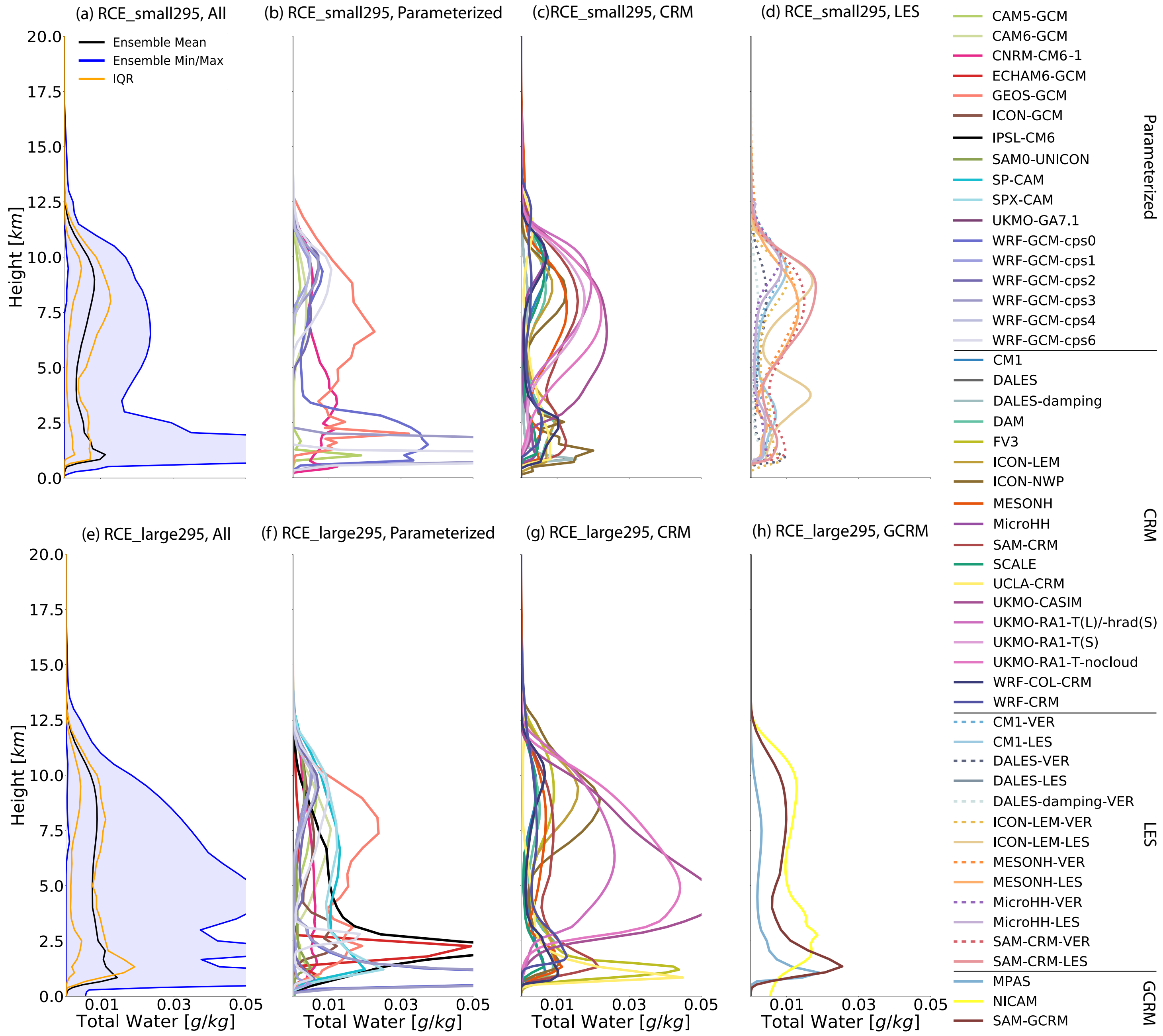

Figure S14. Horizontal-mean total cloud condensate profile, averaged in time excluding the first 75 days of simulation of the `RCE_small` (top row, a-d) and `RCE_large` (bottom row, e-h) simulations at 295 K. TThe first column (a,e) includes all models that performed each type of simulation, where the black line is the ensemble mean, the blue shading shows the range across all models, and the orange lines indicate the interquartile range (IQR). The other columns display each sub-group of models: models with parameterized convection (second column; b,f), CRMs (third column; c,g), models that performed `RCE_small_vert` (dashed) and `RCE_small_les` (solid) simulations (panel d; `RCE_small_les` simulations are averaged over days 25-50), and GCRMs (panel h).

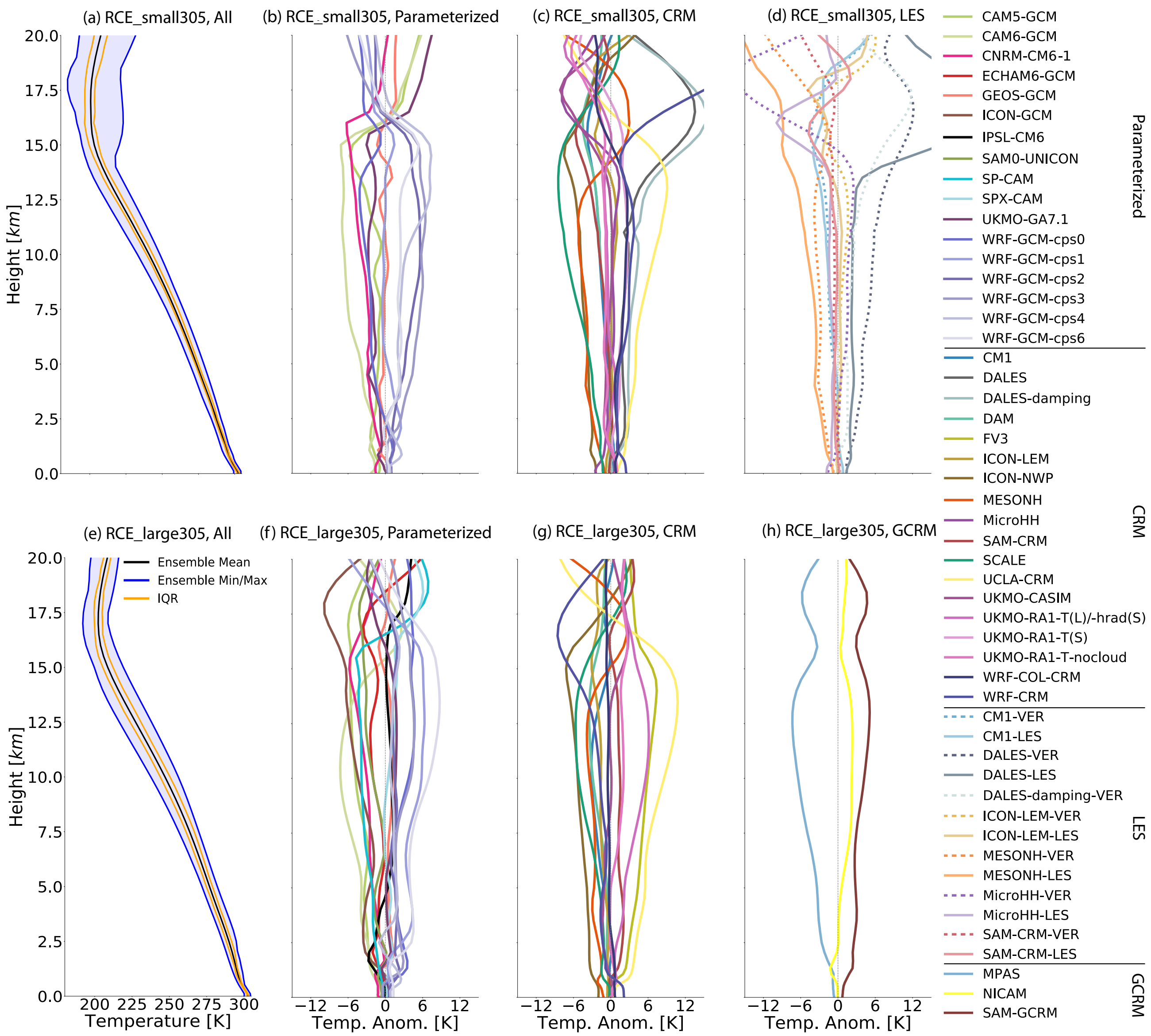

Figure S15. Horizontal-mean temperature profile, averaged in time excluding the first 75 days of simulation of the `RCE_small` (top row, a-d) and `RCE_large` (bottom row, e-h) simulations at 305 K. The first column (a,e) includes all models that performed each type of simulation, where the black line is the ensemble mean, the blue shading shows the range across all models, and the orange lines indicate the interquartile range (IQR). The other columns display the temperature anomaly in each sub-group of models as an anomaly from the ensemble mean of that sub-group, for models with parameterized convection (second column; b,f), CRMs (third column; c,g), models that performed `RCE_small_vert` (dashed) and `RCE_small_les` (solid) simulations (panel d; `RCE_small_les` simulations are averaged over days 25-50), and GCRMs (panel h).

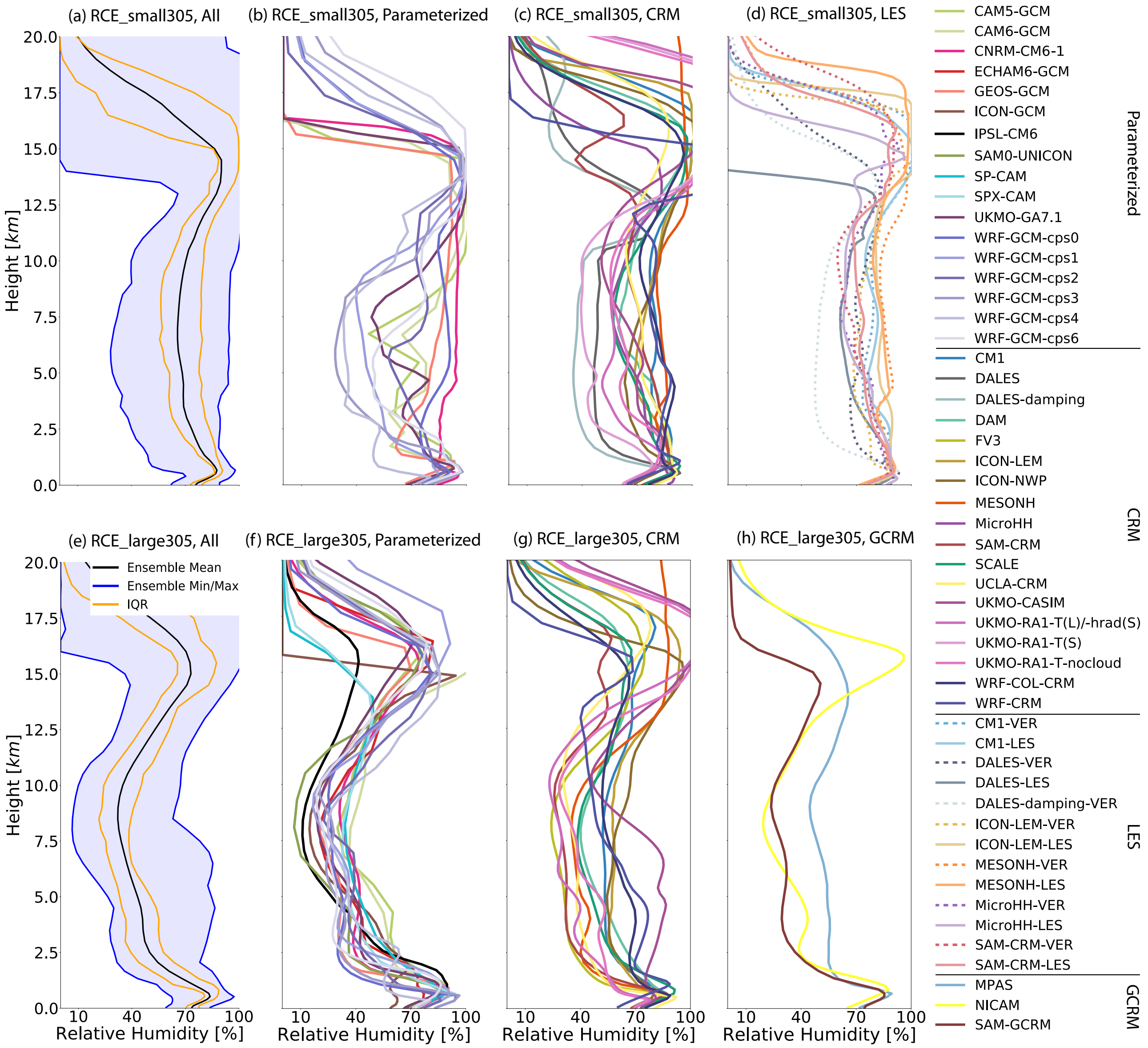

Figure S16. Horizontal-mean relative humidity profile, averaged in time excluding the first 75 days of simulation of the `RCE_small` (top row, a-d) and `RCE_large` (bottom row, e-h) simulations at 305 K. The first column (a,e) includes all models that performed each type of simulation, where the black line is the ensemble mean, the blue shading shows the range across all models, and the orange lines indicate the interquartile range (IQR). The other columns display each sub-group of models: models with parameterized convection (second column; b,f), CRMs (third column; c,g), models that performed `RCE_small_vert` (dashed) and `RCE_small_les` (solid) simulations (panel d; `RCE_small_les` simulations are averaged over days 25-50), and GCRMs (panel h).

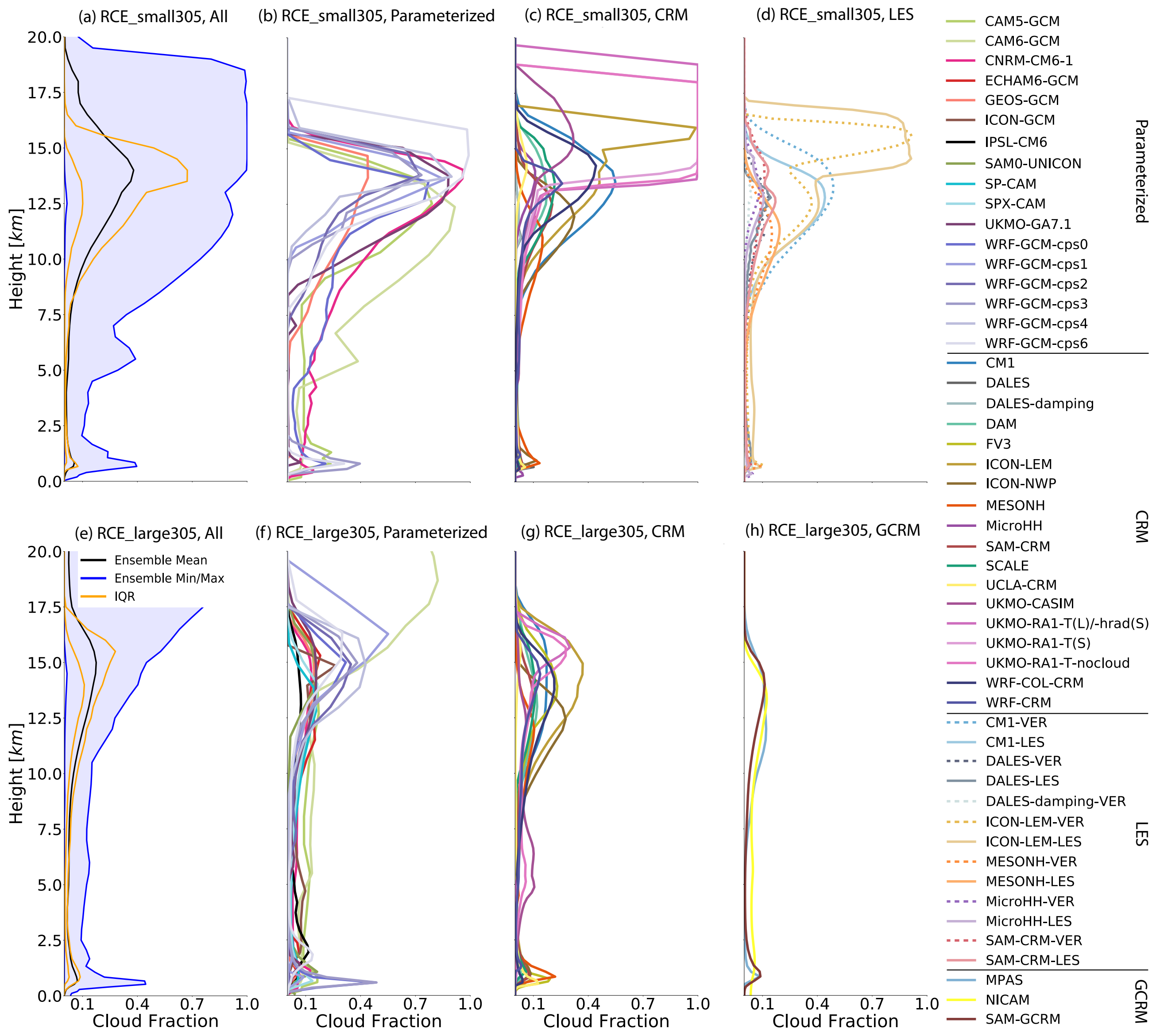

Figure S17. Domain-wide cloud fraction profile, averaged in time excluding the first 75 days of simulation of the `RCE_small` (top row, a-d) and `RCE_large` (bottom row, e-h) simulations at 305 K. The first column (a,e) includes all models that performed each type of simulation, where the black line is the ensemble mean, the blue shading shows the range across all models, and the orange lines indicate the interquartile range (IQR). The other columns display each sub-group of models: models with parameterized convection (second column; b,f), CRMs (third column; c,g), models that performed `RCE_small_vert` (dashed) and `RCE_small_les` (solid) simulations (panel d; `RCE_small_les` simulations are averaged over days 25-50), and GCRMs (panel h).

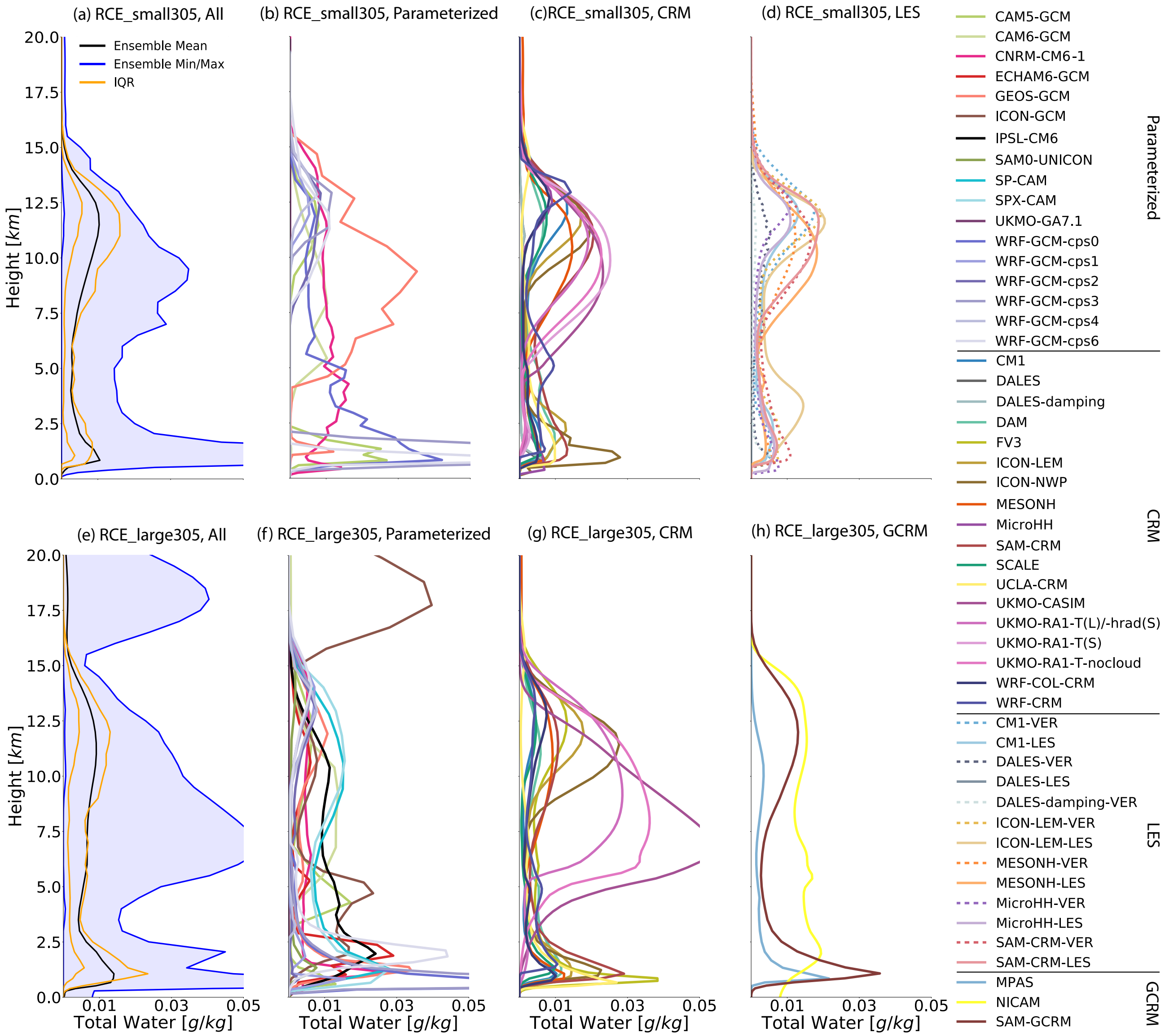

Figure S18. Horizontal-mean total cloud condensate profile, averaged in time excluding the first 75 days of simulation of the `RCE_small` (top row, a-d) and `RCE_large` (bottom row, e-h) simulations at 305 K. The first column (a,e) includes all models that performed each type of simulation, where the black line is the ensemble mean, the blue shading shows the range across all models, and the orange lines indicate the interquartile range (IQR). The other columns display each sub-group of models: models with parameterized convection (second column; b,f), CRMs (third column; c,g), models that performed `RCE_small_vert` (dashed) and `RCE_small_les` (solid) simulations (panel d; `RCE_small_les` simulations are averaged over days 25-50), and GCRMs (panel h).

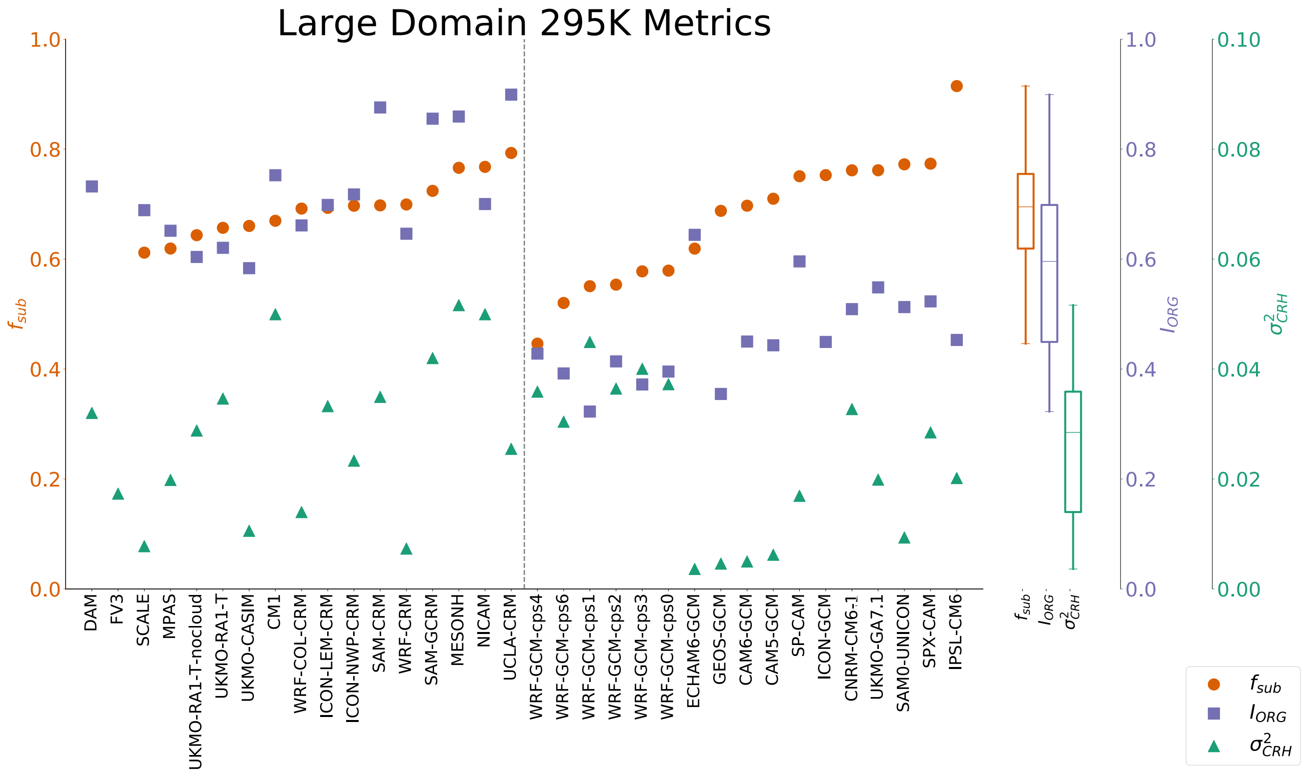

Figure S19. Degree of aggregation in `RCE_large295` based on subsidence fraction (red circles), I_{org} (blue squares), and spatial variance of column relative humidity (green triangles) in all models, averaged in time excluding the first 75 days of simulation. The models are ordered such that the models with explicit convection are to the left of the dashed line and models with parameterized convection are to the right of the dashed line. Within each group of models, they are ordered according to their values of subsidence fraction. The two models for which subsidence fraction could not be computed are listed first. Box plots indicate the spread of each metric across models.

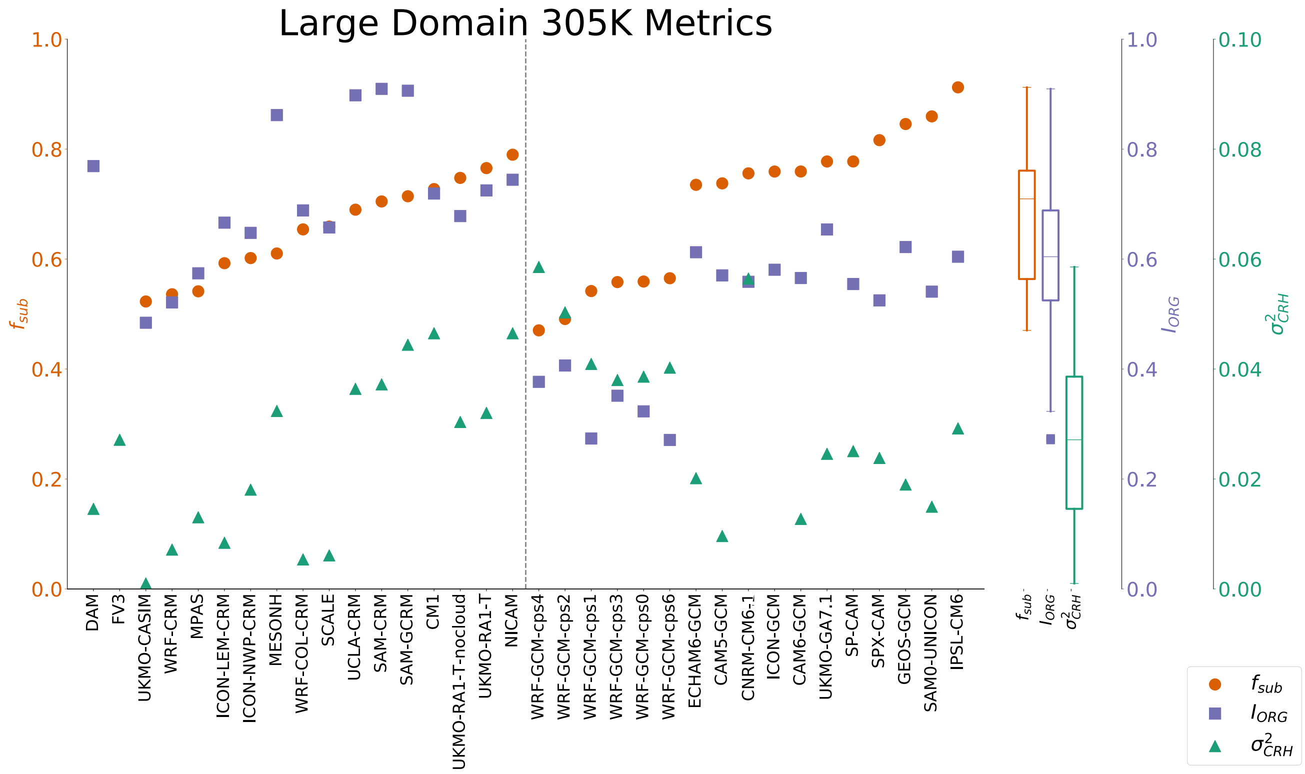

Figure S20. Degree of aggregation in `RCE_large305` based on subsidence fraction (red circles), I_{org} (blue squares), and spatial variance of column relative humidity (green triangles) in all models, averaged in time excluding the first 75 days of simulation. The models are ordered such that the models with explicit convection are to the left of the dashed line and models with parameterized convection are to the right of the dashed line. Within each group of models, they are ordered according to their values of subsidence fraction. The two models for which subsidence fraction could not be computed are listed first. Box plots indicate the spread of each metric across models.

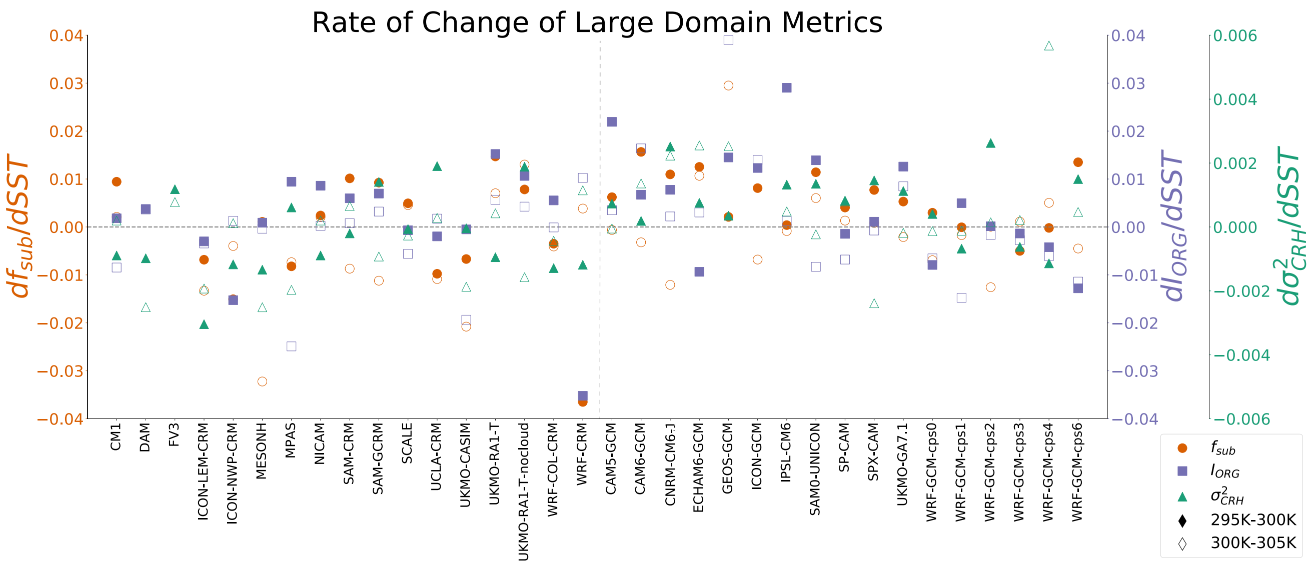

Figure S21. Rate of change of aggregation per degree K based on subsidence fraction (red circles), I_{org} (blue squares), and spatial variance of column relative humidity (green triangles) in all models, based on the rate of change from 295 K to 300 K (filled symbols) and 300 K to 305 K (open symbols). The models are ordered such that the models with explicit convection are to the left of the dashed line and models with parameterized convection are to the right of the dashed line.

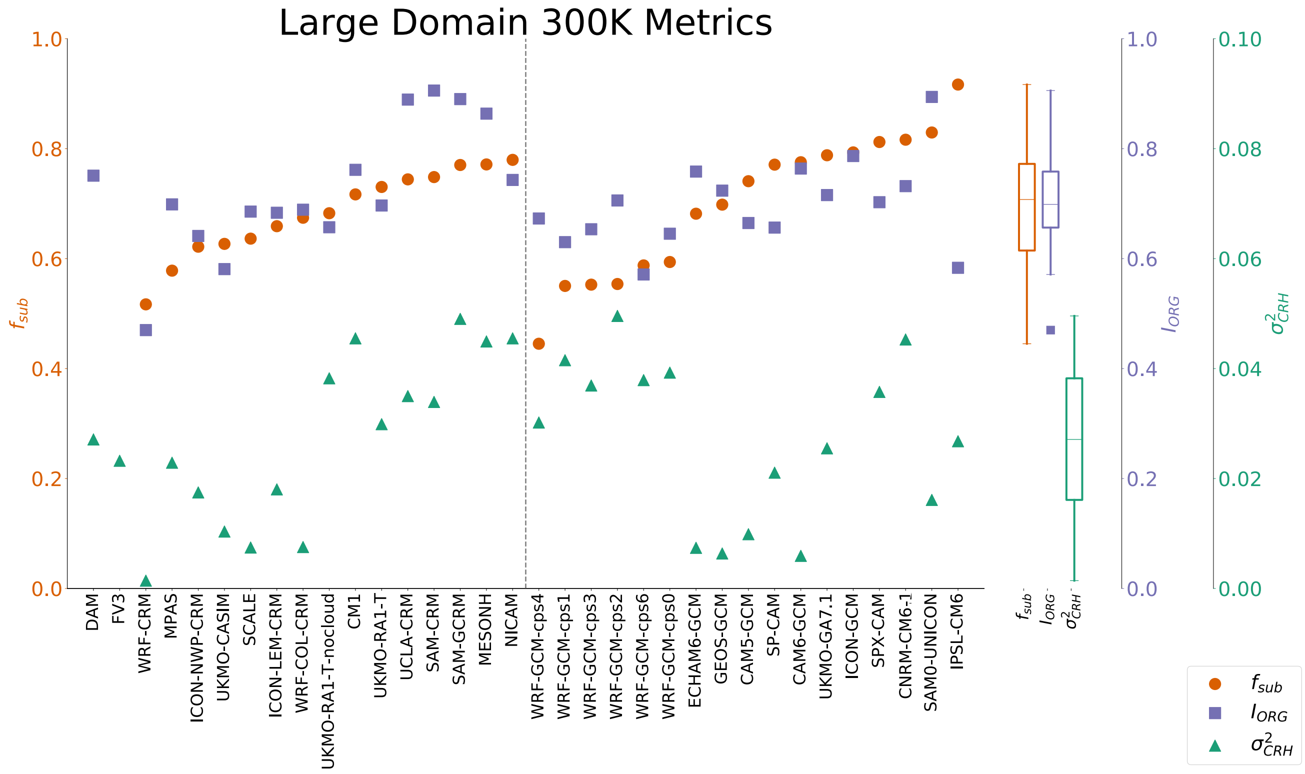

Figure S22. As in Figure 12 in the main text, but using zero connectivity to calculate I_{org} in the GCMs rather than four-point connectivity. Degree of aggregation in `RCE_large300` based on subsidence fraction (red circles), I_{org} (blue squares), and spatial variance of column relative humidity (green triangles) in all models, averaged in time excluding the first 75 days of simulation. The models are ordered such that the models with explicit convection are to the left of the dashed line and models with parameterized convection are to the right of the dashed line. Within each group of models, they are ordered according to their values of subsidence fraction. The two models for which subsidence fraction could not be computed are listed first. Box plots indicate the spread of each metric across models.

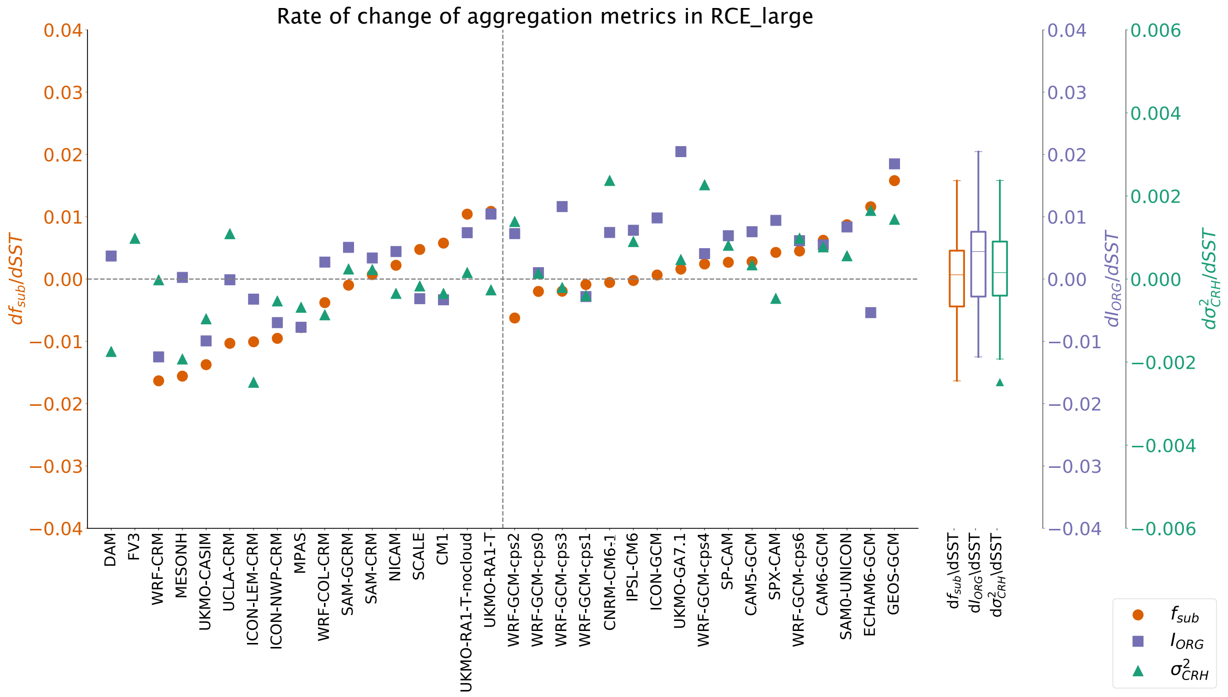

Figure S23. As in Figure 16 in the main text, but using zero connectivity to calculate I_{org} in the GCMs rather than four-point connectivity. Shows the rate of change of the aggregation metrics from 295 K to 3005 K per degree K based on I_{org} (blue squares), subsidence fraction (red circles), and spatial variance of column relative humidity (green triangles) in all models. The models are ordered such that the models with explicit convection are to the left of the dashed line and models with parameterized convection are to the right of the dashed line, each ordered according to their values of $df_{sub}/dSST$. Box plots indicate the spread of each metric's rate of change across models, with outlier indicated with symbols.

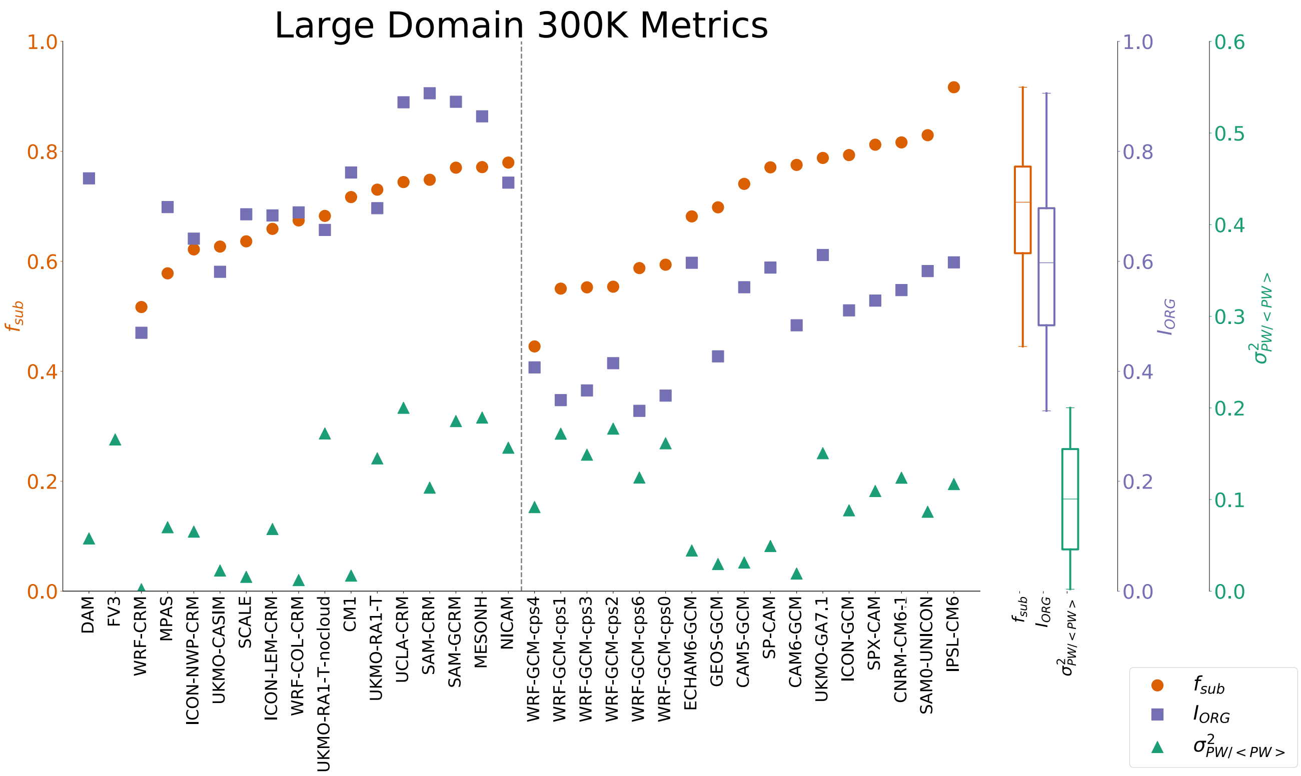

Figure S24. As in Figure 12 in the main text, but using the spatial variance of precipitable water (PW) scaled by its domain mean value (green triangles) rather than column relative humidity. Degree of aggregation in `RCE_large300` based on subsidence fraction (red circles), I_{org} (blue squares), and spatial variance of scaled PW (green triangles) in all models, averaged in time excluding the first 75 days of simulation. he models are ordered such that the models with explicit convection are to the left of the dashed line and models with parameterized convection are to the right of the dashed line. Within each group of models, they are ordered according to their values of subsidence fraction. The two models for which subsidence fraction could not be computed are listed first.

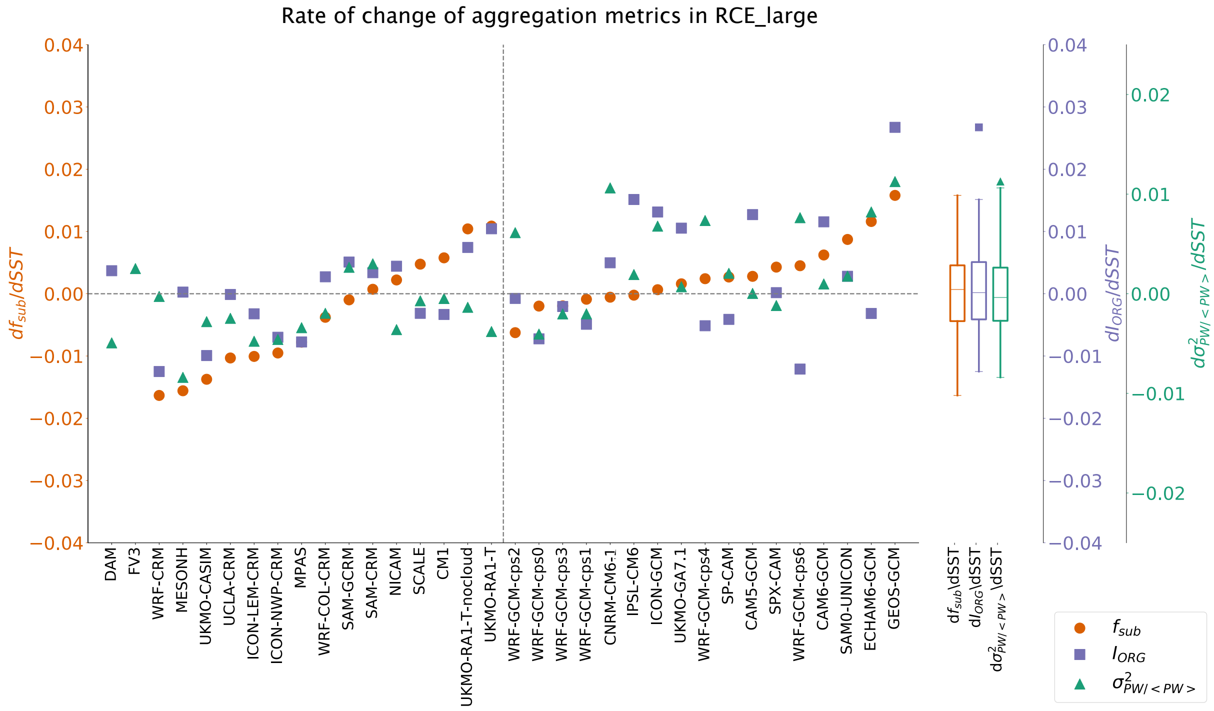

Figure S25. As in Figure 16 in the main text, but using the spatial variance of precipitable water (PW) scaled by its domain mean value (green triangles) rather than column relative humidity. Shows the rate of change of the aggregation metrics from 295 K to 3005 K per degree K based on I_{org} (blue squares), subsidence fraction (red circles), and spatial variance of scaled PW (green triangles) in all models. The models are ordered such that the models with explicit convection are to the left of the dashed line and models with parameterized convection are to the right of the dashed line, each ordered according to their values of $df_{sub}/dSST$. Box plots indicate the spread of each metric's rate of change across models, with outlier indicated with symbols.

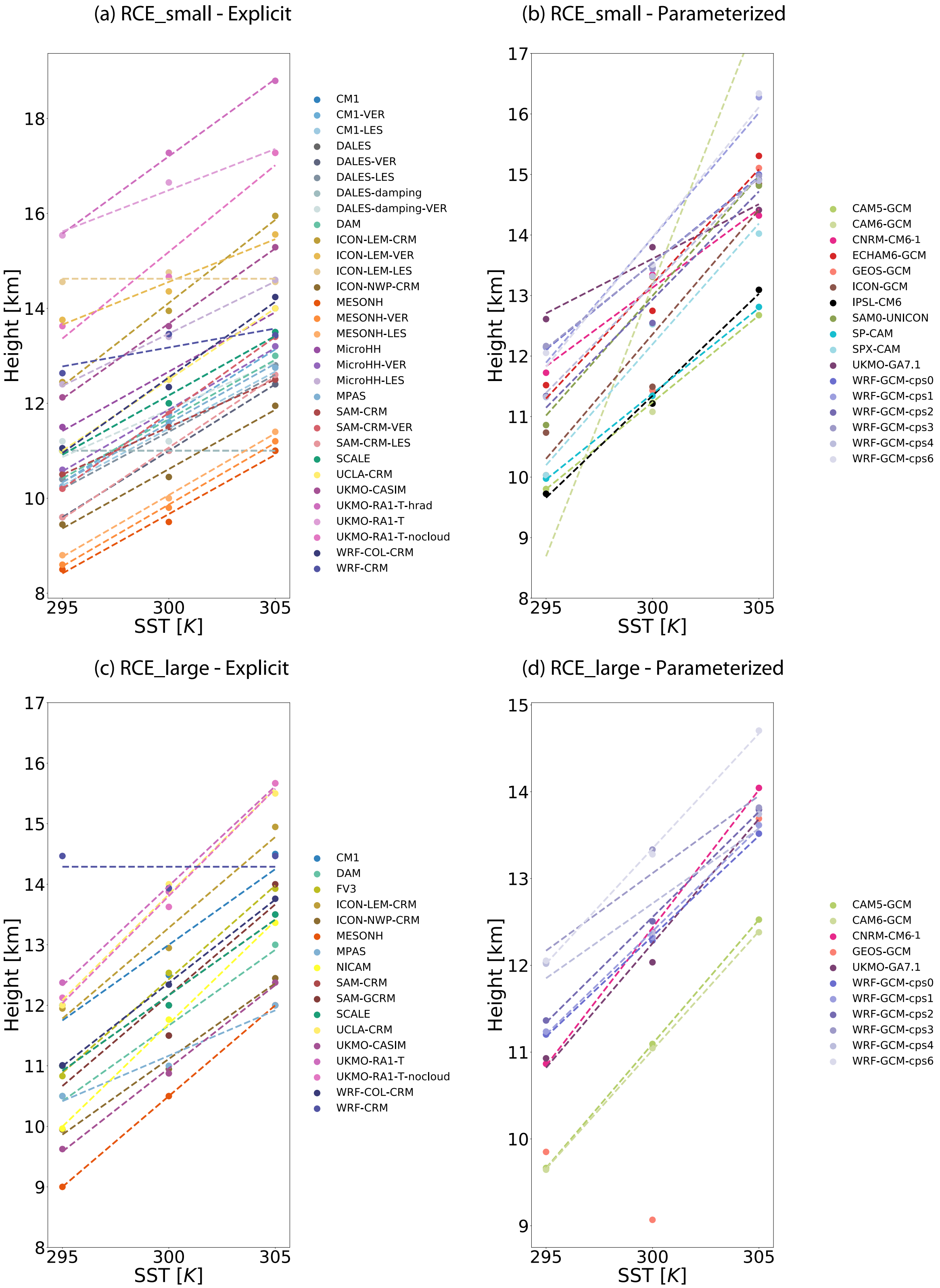

Figure S26. Horizontal- and time-mean height at the location of the domain-wide anvil cloud peak as a function of SST in the `RCE_small` simulations (a,b) and `RCE_large` simulations (c,d), for models with explicit (a,c) and parameterized (b,d) convection. The dashed lines are linear regression lines of best fit.

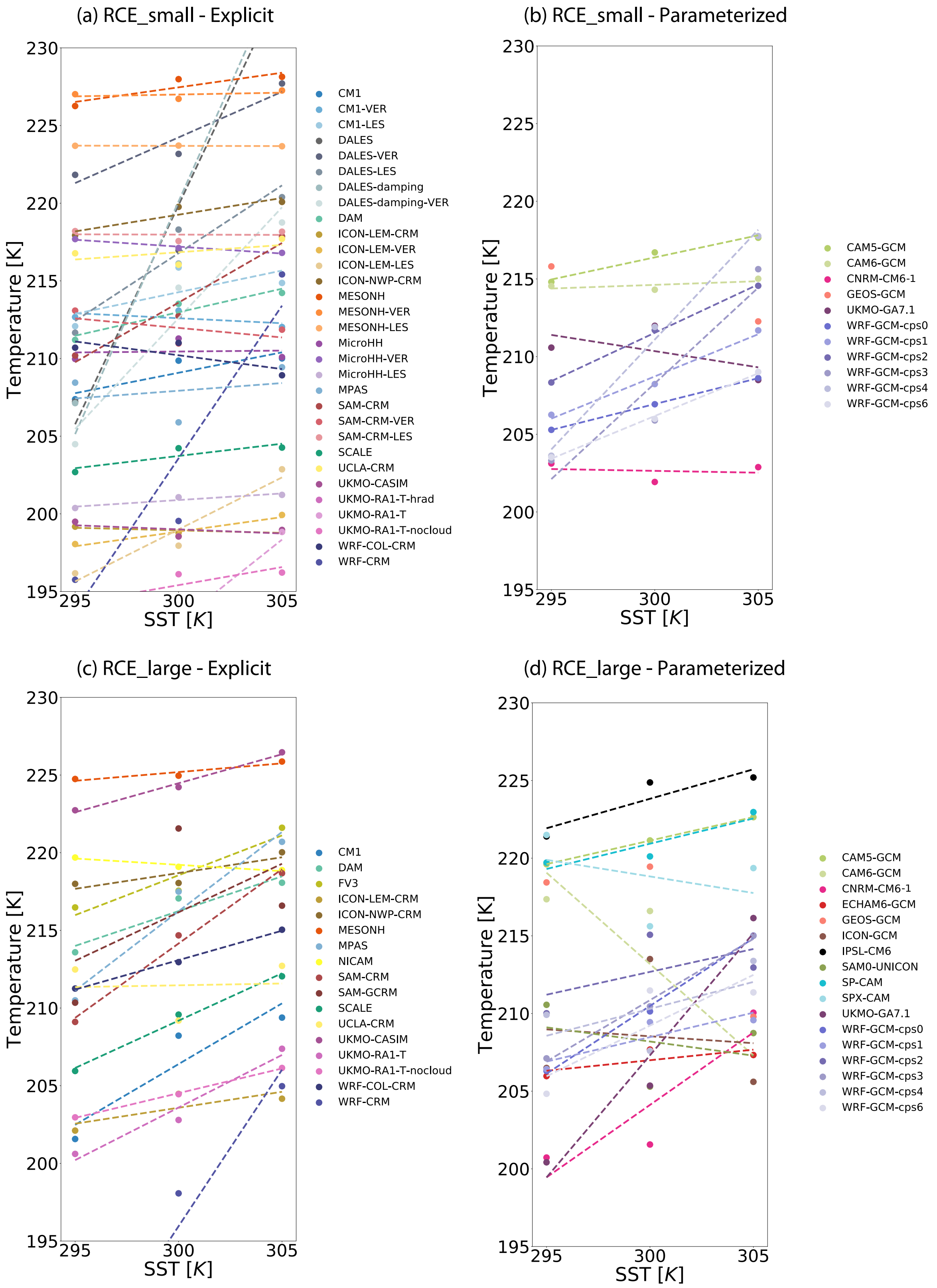

Figure S27. Horizontal- and time-mean temperature at the location of the domain-wide anvil cloud peak as a function of SST in the `RCE_small` simulations (a,b) and `RCE_large` simulations (c,d), for models with explicit (a,c) and parameterized (b,d) convection. The dashed lines are linear regression lines of best fit.

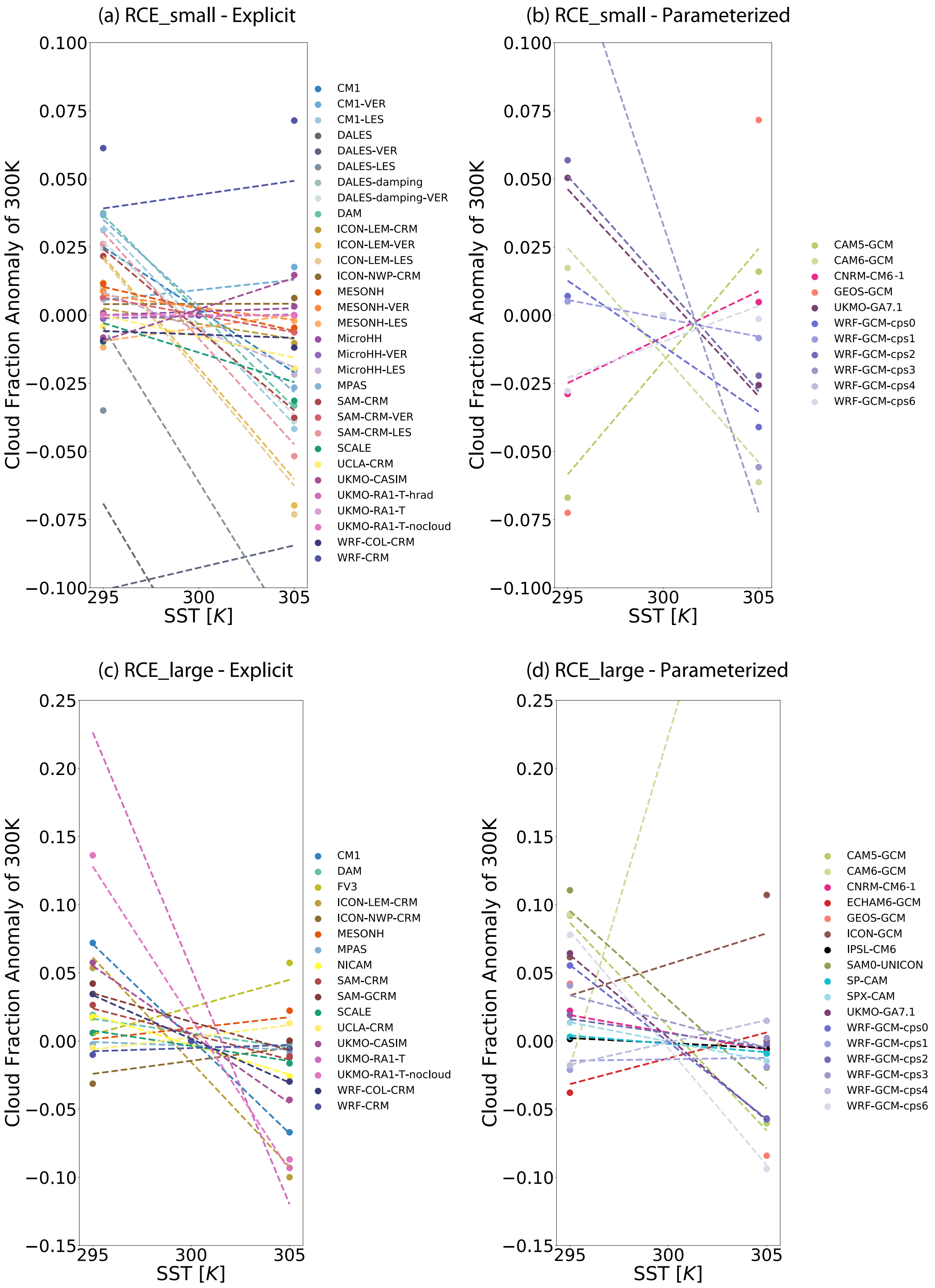

Figure S28. Domain-wide anvil cloud fraction as a function of SST in the `RCE_small` simulations (a,b) and `RCE_large` simulations (c,d), for models with explicit (a,c) and parameterized (b,d) convection. The anvil cloud fraction is shown as anomaly from its value in the simulation at 300 K. The dashed lines are linear regression lines of best fit.
